# Genomic and Proteomic Analysis of the Impact of Mitotic Quiescence on the Engraftment of Human CD34^+^ Cells

**DOI:** 10.1371/journal.pone.0017498

**Published:** 2011-03-07

**Authors:** Brahmananda Reddy Chitteti, Yunlong Liu, Edward F. Srour

**Affiliations:** 1 Department of Medicine, Indiana University School of Medicine, Indianapolis, Indiana, United States of America; 2 Department of Biostatistics, Indiana University School of Medicine, Indianapolis, Indiana, United States of America; 3 Department of Pediatrics, Indiana University School of Medicine, Indianapolis, Indiana, United States of America; 4 Department of Microbiology and Immunology, Indiana University School of Medicine, Indianapolis, Indiana, United States of America; University of Sao Paulo – USP, Brazil

## Abstract

It is well established that in adults, long-term repopulating hematopoietic stem cells (HSC) are mitotically quiescent cells that reside in specialized bone marrow (BM) niches that maintain the dormancy of HSC. Our laboratory demonstrated that the engraftment potential of human HSC (CD34^+^ cells) from BM and mobilized peripheral blood (MPB) is restricted to cells in the G0 phase of cell cycle but that in the case of umbilical cord blood (UCB) -derived CD34^+^ cells, cell cycle status is not a determining factor in the ability of these cells to engraft and sustain hematopoiesis. We used this distinct *in vivo* behavior of CD34^+^ cells from these tissues to identify genes associated with the engraftment potential of human HSC. CD34^+^ cells from BM, MPB, and UCB were fractionated into G0 and G1 phases of cell cycle and subjected in parallel to microarray and proteomic analyses. A total of 484 target genes were identified to be associated with engraftment potential of HSC. System biology modeling indicated that the top four signaling pathways associated with these genes are Integrin signaling, p53 signaling, cytotoxic T lymphocyte-mediated apoptosis, and Myc mediated apoptosis signaling. Our data suggest that a continuum of functions of hematopoietic cells directly associated with cell cycle progression may play a major role in governing the engraftment potential of stem cells. While proteomic analysis identified a total of 646 proteins in analyzed samples, a very limited overlap between genomic and proteomic data was observed. These data provide a new insight into the genetic control of engraftment of human HSC from distinct tissues and suggest that mitotic quiescence may not be the requisite characteristic of engrafting stem cells, but instead may be the physiologic status conducive to the expression of genetic elements favoring engraftment.

## Introduction

Life-long maintenance of the hematopoietic system is sustained by highly specialized hematopoietic stem cells (HSC) [1], [2], [3]. In steady state, HSC are highly dormant undergoing self-renewal divisions rather infrequently. However, the molecular mechanisms governing their mitotic quiescence are largely unknown. During mammalian development, stem cells first appear in the yolk sac, then migrate into the fetal liver, and finally migrate into bone marrow (BM). We previously completed [4], [5], [6] a survey of the potential of cycling and non-cycling HSC from human hematopoietic tissues through ontogeny to engraft in conditioned NOD/SCID recipients. When human mobilized peripheral blood (MPB) and BM CD34^+^ cells in G0 or G1 phase of cell cycle were examined, only those in G0 were capable of *in-vivo* long-term multilineage engraftment [4]. In contrast, both mitotically quiescent as well as cycling HSC from umbilical cord blood (UCB), fetal liver and human fetal bone marrow [5], [6] retained their ability to engraft in NOD/SCID mice. Collectively, these studies established that in adult tissues, a hierarchical order of hematopoietic potential can be assembled based on the mitotic status of HSC whereby only cells in G0 engraft. On the other hand, in the case of prenatal HSC (including UCB) such a hierarchy does not predominate and both cycling (cells in G1) and quiescent (cells in G0) cells retain their hematopoietic potential. It is therefore possible that genes mediating *in vivo* stem cell engraftment function may be differentially expressed in adult BM and MPB CD34^+^G0 cells, and UCB CD34^+^G0 and G1 cells, but not in adult MPB and BM CD34^+^ cells in G1. Alternatively, one has to consider that if any of the continuum models of stem cell function that have been proposed [7], [8], [9], [10] is operative, then a change in gene expression between cells in G0 versus those in G1, that may control the ability of cells to engraft, should still be detectable even if all cell cycle regulation genes were eliminated from further analysis. The availability of six groups of human CD34^+^ cells from three distinct tissues with previously established functional capabilities allowed us to carefully investigate the genetic control of pathways implicated in engraftment and to examine the degree of homogeneity or heterogeneity between functionally similar (all G0 groups of cells and G1 cells from UCB) but phenotypically different (G0 and G1 cells from UCB) groups of cells in the absence of the impact of cell cycle regulatory genes.

Although microarrays are informative in their ability to measure biological differences at the mRNA level [11], most functional processes are executed by proteins which become therefore the more relevant parameter for the assessment of operational mechanisms [12]. Recent advances in analyzing global protein expression profiles and in label-free quantification have demonstrated the potential for comparative proteomic studies. Although several studies demonstrated the presence of moderate to poor correlations between microarray and proteomic analyses [13], [14], [15], [16], implementing both methods may generate complementary and more informative data that cannot be obtained by either method alone. Recently, microarray and proteomic analyses of human and mouse stem cells generated insights into the molecular composition of stem cell profiles [17], [18], [19], [20], [21], [22], [23], [24], [25]. In the present study, we investigated the global gene and protein expression profiles of G0 and G1 cells from human BM, MPB, and UCB-derived CD34^+^ cells by whole genome microarrays and mass spectrometry based proteomic techniques, respectively. Our data provide a unique comparative evaluation of the genomic and proteomic profiles of well-characterized groups of human HSC and illustrate that these analyses may not necessarily generate complementary or compatible results. Furthermore, our data suggest that gene expression patterns in HSC may oscillate in a cell cycle related manner to confer engraftment potential on cells in one or more phases of cell cycle depending on the developmental stage and need for functional HSC.

## Materials and Methods

### Human CD34^+^ cells

BM and UCB samples were ficolled and mononuclear cells were collected followed by CD34^+^ selection using Miltenyi Magnetically Activated Cell Sorting (MACS) columns according to the manufacturer's directions (Miltenyi Biotec GmbH, Bergisch Gladbach, Germany). Mobilization was achieved by daily granulocyte colony-stimulating factor administration at 5 mg/kg (maximum of 480 mg/day) for 4 consecutive days. Apheresis was performed on day 5 and CD34^+^ cells were isolated by immunomagnetic selection on an Isolex 300i system (Nexell, Irvine, CA). To generate distinct and unique sets of data, we did not pool multiple samples from any tissue studied so that each sample or its replicate was from a single donor. Purity of selected CD34^+^ fractions was assessed by flow-cytometric analysis with an antibody recognizing a different CD34 epitope (BD Biosciences, San Jose, CA). The efficiency of the MACS columns was over 80% and the efficiency of the Isolex 300i system exceeded 95%. All samples of selected CD34^+^ cells were cryopreserved prior to their use.

### Ethics Statement

All studies described in this manuscript were approved by the Investigational Review Board of Indiana University School of Medicine. BM and MPB samples were collected from healthy adult volunteers after obtaining a written and signed informed consent according to the guidelines of our institutional IRB.

### Cell-cycle fractionation with Hoechst 33342 (Hst) and Pyronin Y (PY)

**Figure 1:**
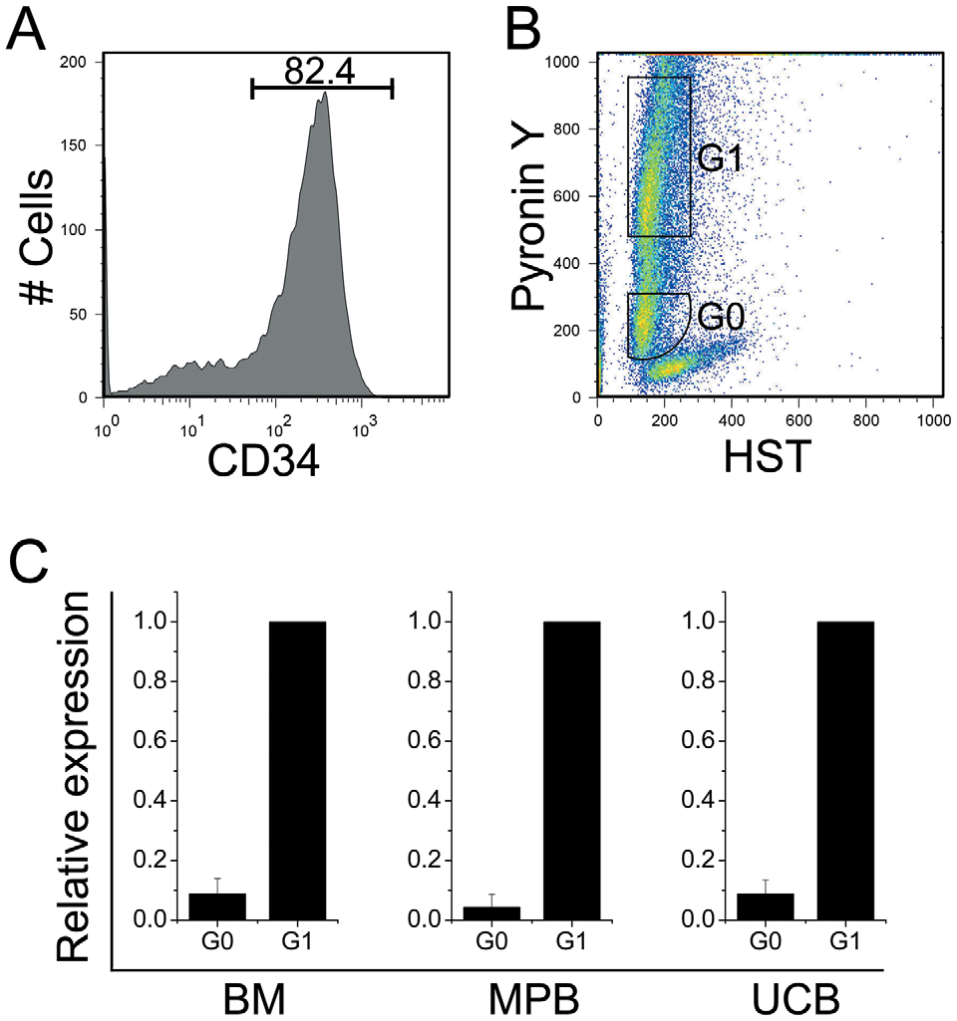
A representative figure showing typical flow cytometric cell sorting of BM, MPB, or UCB CD34^+^ cells into G0 and G1 phases of cell cycle. CD34^+^ cells selected on a Miltenyi MACS column were stained with APC conjugated CD34 antibody, Hst and PY. (**A**): CD34 positive cells were gated and analyzed for Hst and PY. (**B**): Quiescent cells residing in G0 phase have 2n DNA and minimal RNA content, whereas those in early or late G1 phase are PY bright owing to their higher RNA content. According to this definition, G0 and G1 cells were sorted based on their relative Hst and PY intensities. At least 150 fluorescence channels separated the 2 sort windows. (**C**): In order to confirm the purity of sorted cells, post sort analysis was carried out by measuring the relative expression of a cell cycle marker, Ki67 by qRT-PCR. About 10,000 cells from each sorted group were analyzed by qRT-PCR using a TaqMan probe and ABI 7800 real time PCR machine.

To distinguish cells in G0 or G1, which have the same DNA but different RNA content, simultaneous DNA/RNA staining with Hoechst 33342 (Molecular Probes, Eugene, OR) and Pyronin Y (Polysciences, Warrington, PA), respectively, was performed as previously described [26], [27]. At the end of the staining with Hst and PY, cells were washed once in ice-cold Hst buffer and incubated for 30 minutes with APC–conjugated CD34 at 4°C. Cells were washed again, resuspended in Hst buffer, and analyzed or sorted on FACS Aria (BDIS). Cells in G0 (G0CD34^+^) were identified by their 2n DNA and minimal RNA content; cells in G1 (G1CD34^+^) were defined as those with 2n DNA and high RNA staining [28]. Cell sorting criteria were identical to those previously described [4] allowing us to separate the upper limit of the G0 sort window from the lower limit of the G1 sort window by at least 150 fluorescence channels. Viability of sorted cells always exceeded 98%. A representative dot plot is shown in Figure 1A and 1B. From each sort window, two separate aliquots of 10^4^ and 2×10^5^ cells were sorted for genomic and proteomic analyses, respectively.

### Quantitative Real-Time PCR

To confirm the cell cycle status of sorted G0CD34^+^ and G1CD34^+^ cells, their relative expression of Ki67 was measured by qRT-PCR. cDNA was made on μMACS columns (Miltenyi Biotec, Auburn, CA) using μMACS one-step cDNA kit (Miltenyi Biotec, Cat # 130-091-902) following the manufacturer's instructions. qRT-PCR was performed using Taqman probe following the manufacturer's instructions (Applied Biosystems, Foster City, CA). PCR amplification was performed in a 20-µl final volume containing 10 µl 2× Taqman universal PCR master mix, 1 µl of 20× Ki67 assay mix, 9 µl template (cDNA diluted) at 50°C for 2 min, 95°C for 10 min, followed by 40 cycles at 95°C for 15 sec and 60°C for 1 min. Expression of GAPDH was used for normalization. Cells were acceptable for further analysis if the relative expression of Ki67 among G0CD34^+^ cells was 90–95% lower than that detected in G1CD34^+^ cells from all 3 tissues (Figure 1C).

### Microarray analysis

Triplicate samples of G0 and G1 cells from BM, MPB, and UCB (total of eighteen samples) were lysed in Miltenyi Super Amp lysis buffer and the downstream microarray analysis was carried out by Miltenyi Biotec. Briefly, mRNA was made using magnetic bead technology followed by cDNA preparation and amplification. cDNA samples were quantified using an ND-1000 Spectrophotometer (NanoDrop Technologies) and cDNA integrity was checked via the Agilent 2100 Bioanalyzer platform. 250 ng of the cDNA were labeled with Cy3 and hybridized overnight (17 hours, 65°C) to an Agilent Whole Human Genome Oligo Microarrays. Finally, the microarrays were washed and fluorescence signals of the hybridized Agilent Microarrays were detected using Agilent's Microarray Scanner System. The Agilent Feature Extraction Software (FES) was used to read out and process the microarray image files. For determination of differential gene expression, FES derived output data files were analyzed using the Rosetta Resolver gene expression data analysis system (Rosetta Biosoftware). Signal intensities from the single-experiment raw data lists were normalized by dividing the intensity values by their median. Standard deviation and p-values were calculated for each probe. Differentially expressed genes with at least two-fold change and p-value<0.01 were considered for further analysis. Gene clustering analysis was done using TM4 MultiExperiment Viewer (version 4.3) [29]. The metadata and raw data files of microarray experiments (all MIAME compliant) were deposited in a MIAME compliant database, Gene Expression Omnibus (Accession numbers GSM595960 to GSM595977, a total of 18 samples).

### Protein extraction and sample preparation for Proteomic analysis

Samples for mass spectrometry were prepared as previously described [30]. Briefly, 3 to 5 biological replicates of G0 and G1 cells from BM, MPB, and UCB were homogenized in hypotonic lysis buffer containing freshly made 8 M urea and 10 mM DTT solution. Proteins were reduced with 20 µL of 200 mM DTT in 100 mM Tris-Cl (pH 7.8) for 1 h at room temperature, alkylated with 20 µL of 200 mM iodoacetamide in 100 mM Tris-Cl (pH 7.8) in the dark for 1 h, and diluted to a final urea concentration of 0.6 M, a concentration at which trypsin retains its activity. Trypsin solution was added to a final ratio of enzyme to substrate of 1∶50. Digestion was carried out at 37°C and stopped 15 h later by adding 10 mL of 10 mM lysine. pH was then adjusted to below 6.0 and vacuum dried to a final volume of 25 mL. Peptide concentration was determined by the Bradford protein assay [31]. Peptide mixtures were subjected to LC/MS.

### Mass spectrometry and protein quantification

Mass spectrometry analysis was carried out as previously described [30]. Briefly, using a Surveyor HPLC system (Thermo-Finnigan), all tryptic peptides were injected onto a C18 microbore column (Zorbax 300SB-C18) in a random order. Peptides were eluted from the column by acetonitrile linear gradient from 5 to 45 developed over 120 min at a flow rate of 50 mL/min. Eluted peptides were directly electro-sprayed into an LTQ mass spectrometer (Thermo-Finnigan). Data were collected in the “Triple-Play” mode and acquired data were filtered and analyzed by a proprietary algorithm developed and described by Higgs *et al* [32]. Using X!Tandem and SEQUEST algorithms, we searched the database against the International Protein Index (IPI) human database and the nonredundant-homo sapiens database. Protein quantification and differential expression of proteins were done using a proprietary software licensed from Eli Lilly and Company [32].

### Gene Ontology and Pathway analysis

Candidate genes or proteins were analyzed for their gene ontology and pathway analysis using the Database for Annotation, Visualization and Integrated Discovery (DAVID), sixth version, the web based program [33], [34] and Pathways Analysis software 7.5 (IPA, Ingenuity Systems, Mountain View, California). The provided list of genes was mapped to Ingenuity Pathways Knowledgebase (IPKB) and the functional categorization and the significance of these genes in biological pathways was drawn. Fisher exact test was used to calculate P-values. P≤0.05 was used to select significant biological functions and pathways associated with candidate genes.

## Results

### Differential gene expression profile of G0 and G1 cells from BM, MPB, and UCB

**Figure 2:**
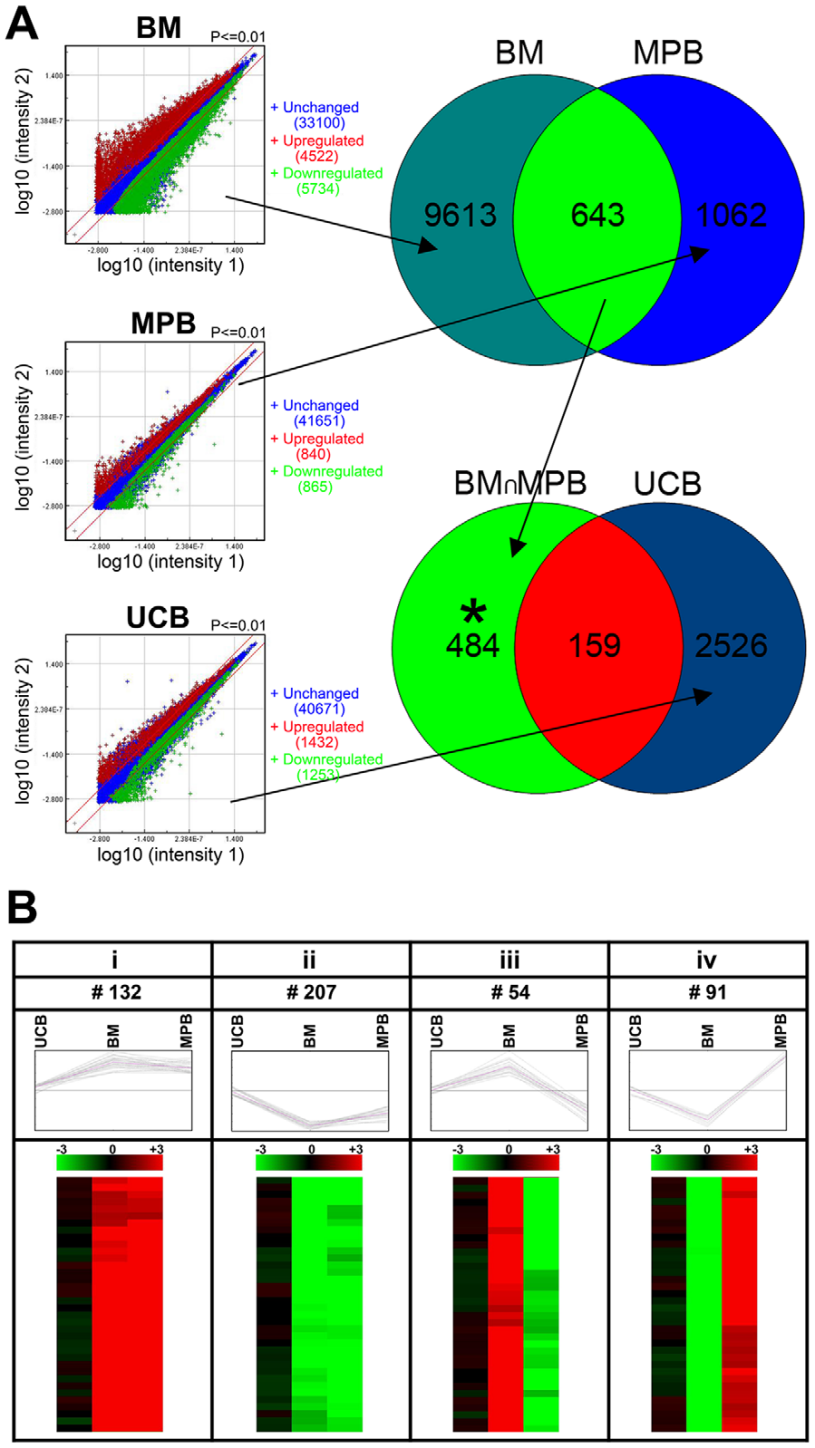
Genomic analysis of human BM, CB, and MPB CD34+ cells in different phases of cell cycle. (**A**) Microarray analyses of G0CD34^+^ and G1CD34^+^ cells from BM, MPB, and UCB (3 replicates per group, total of 18 samples) were carried out using Agilent whole human genome oligo chips. The signal intensities from the single-experiment raw data lists were normalized by dividing the intensity values by their median. Standard deviation and p-values were calculated for each probe. The differentially expressed genes with at least two fold change and p-value<0.01 were considered as differentially expressed genes. Among the 43,356 total analyzed genes, 10256, 1705, and 2685 genes were differentially expressed between G0 and G1 cells of BM, MPB, and UCB, respectively. In order to identify target genes related to engraftment, common differentially expressed genes between G0 and G1 from both BM and MPB were identified (643 genes). A total of 159 differentially genes between G0 and G1 cells of UCB were common with these 643 genes. Considering that these 159 genes were related to progression of cells from G0 to G1 and therefore not involved in engraftment, our analysis focused on the difference between these two sets of genes, namely, 484 genes (643−159 = 484). * = target genes. (**B**): Gene clustering analyses of 484 target genes were done using open source software TM4 MultiExperiment Viewer (version 4.3). Out of 484 differentially regulated genes, (i) 132 genes were upregulated in G0 cells of both BM and MPB (unchanged in UCB), and (ii) 207 genes were upregulated in G1 cells of both BM and MPB (unchanged in UCB). The remaining genes displayed as aberrant expression patterns. (iii) 54 genes were upregulated in BM G0 cells and MPB G1 cells, and (iv) 91 genes were upregulated in BM G1 and MPB G0 cells.

To identify genes differentially expressed between quiescent (G0) and cycling (G1) cells, we examined the global mRNA expression profiles of sorted and qRT-PCR verified G0 and G1 cells from BM, MPB, and UCB. Among the analyzed 43,356 total genes, 2685 genes were differentially expressed between G0 and G1 cells from UCB. Of these, 1432 genes were upregulated in G0 cells whereas 1253 genes were upregulated in G1 cells (Rather than using conventional terminology to describe these 1253 genes as “downregulated” in G0, we will instead refer to these as “upregulated” in G1 throughout the manuscript). In case of MPB, 1705 genes were differentially expressed; 840 genes were upregulated in G0 cells and 865 genes were upregulated in G1 cells. In contrast, BM derived CD34^+^ cells were very dynamic whereby 10,256 genes (23.6% of total genes), were differentially expressed between G0 and G1 cells (4522 upregulated in G0 cells and 5734 upregulated in G1 cells). Only 159 differentially expressed genes between G0 and G1 cells were common for all three tissues (Figure 2A).

### Rationale for the identification of engraftment related target genes

Differential engraftment of CD34^+^ cells from different sources gave us a unique opportunity to identify target genes responsible for engraftment by eliminating differentially expressed genes associated with the traverse of cells from G0 to G1. To proceed with our analysis, we made three assumptions. First, in the case of BM, since only G0 cells engrafted, we assumed that genes responsible for engraftment were differentially expressed between BM-G0 and BM-G1. Second, we assumed that since BM and MPB are similar in terms of their engraftment profile, candidate genes identified in BM should also be identified in MPB. On the other hand, since both UCB G0 and G1 cells can engraft, genes that are responsible for engraftment must be similarly (either up or down) expressed between G0 and G1. Our third assumption was that genes differentially expressed between UCB G0 and G1 are primarily cell cycle related and are most likely not involved directly with the engraftment potential of these cells. Therefore, we hypothesized that candidate genes responsible for engraftment may be identified by the subtraction of differentially expressed genes between G0 and G1 of UCB from those that are differentially expressed between G0 and G1 of BM and MPB. To corroborate the third assumption, we phenotypically compared markers of hematopoietic differentiation between UCB G0 and G1 cells. As can be seen in Figure S1, the expression patterns of 10 hematopoietic markers were identical between UCB G0 and G1 cells demonstrating that the phenotypic makeup of these two groups did not significantly impact the profiles obtained. For added comparisons, we also analyzed BM G0 and G1 cells and obtained the same profiles between both groups (Figure S1).

We identified 643 common differentially expressed genes between both BM and MPB G0 and G1 cells and 159 differentially expressed genes between UCB G0 and G1 cells were common with these 643 genes. Based on our proposed model, we were left with 484 (643−159 = 484) target genes that are most likely not cell cycle related but important for the control of engraftment of HSC from all three tissues. This rationale is depicted in Figure 2A and the list of the 484 target genes along with their relative expression is shown in Table S1.

### Cluster analysis

We carried out hierarchical cluster analysis of the target genes using open source software TM4-MultipleExperiment Viewer [29], [35]. We chose default Euclidean distance metric and average linkage clustering method to analyze the data. Out of 484 target genes, 132 were upregulated in G0 cells of both in BM and MPB (unchanged in UCB), 207 were upregulated in G1 cells of both BM and MPB, and unchanged in UCB. The remaining 145 genes showed aberrant expression patterns (Figure 2B). Therefore, further gene ontology analysis was carried out on the 339 (132+207 = 339) genes that showed the same expression pattern both in BM and MPB and remained unchanged in UCB.

### Gene Ontology (GO) and Pathway analyses

**Figure 3:**
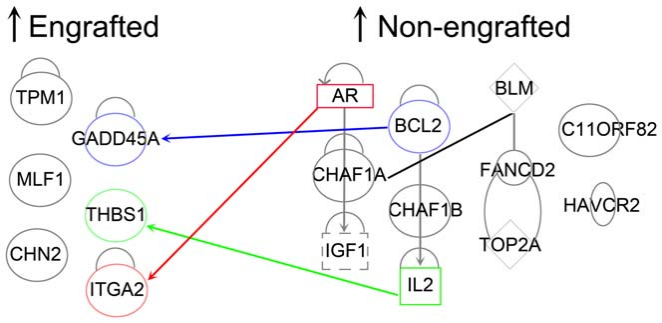
Candidate genes were analyzed by Ingenuity pathway analysis software. Upregulated genes in engrafted cells (G0 cells of BM, MPB; G0 and G1 cells of UCB) and upregulated genes in non-engrafted cells (G1 cells of BM and MPB) were put side by side and direct and indirect relations between the two sets were drawn. Several genes that are upregulated in non-engrafted cells have an inverse function on the genes that are upregulated in engrafted cells or vice versa. Genes represented with blue, green, and red arrow lines have opposite functions.

Microarray identified target genes were further classified following the GO rules based on: (i) the cellular component (CC) indicating where the gene product can be found; (ii) the biological process (BP) in which the gene product participates; (iii) the molecular function (MF) that describes the gene product activities. As shown in Table 1, the top molecular and cellular functions associated with genes upregulated in engrafted cells were cellular movement, antigen presentation, cell signaling, molecular transport, and nucleic acid metabolism. Whereas, genes upregulated in non engrafted cells were mostly associated with cellular growth and proliferation, cell cycle, cellular assembly and organization, and DNA replication. Interestingly, nine genes (ADAMTS1, THBS1, TIMP3, PTGS1, NCKAP1, EVI1, MFGE8, ITGA2, ENST00000353442, p = 2.62E-04 - 3.80E-02) with “embryonic development function” were also upregulated in G0 cells of BM and MPB but relatively unchanged in G0 and G1 of UCB (Table 2). The top canonical pathways associated with engrafted cells were: integrin signaling (e.g. DIRAS3, ITGA2, TSPAN6), P53 signaling (e.g. GADD45A, THBS1), oncostatin M signaling (e,g. TIMP3), T helper cell differentiation (e.g. IL18R1) whereas the top canonical pathways associated with non-engrafted cells were: Interleukin signaling (e.g. IL2, IL8, IL15), myc mediated apoptosis signaling (e.g. IGF1, BCL2), and cytotoxic T lymphocyte mediated apoptosis. Interestingly, several genes upregulated in engrafted cells had an inverse function on genes upregulated in non-engrafted cells and vice versa (Figure 3). These inverse relationships will be discussed in some detail further below.

**Table 1:** 

(A): Top molecular and cellular functions of differentially upregulated genes in engrafted G0 cells.			
Name	p-value	#	Molecules
Cellular movement	6.90E-04 - 4.94E-02	12	CHN2, CXCL2, CXCL3, GADD45A, IL18R1, ITGA2, NCKAP1, SHC4, SORT1, THBS1, TIMP3, TPM1
Antigen presentation	8.69E-04 - 4.02E-02	8	CXCL2, CXCL3, EVII, FCER1A, IL18R1, MFGE8, PTGS1, THBS1
Cell signaling	9.25E-04 - 4.50E-02	10	ABCC4, CXCL3, DIRAS3, FCER1A, GADD45A, ITGA2, LPAR4, P2RY12, PTGS1, THBS1
Molecular transport	9.25E-04 - 4.94E-02	15	ABCC4, ARG2, CXCL3, DIRAS3, FCER1A, ITGA2, LPAR4, P2RY12, PTGS1, RCRC1, SLC16A6, SLC25A21, SLC40A1, SORT1, THBSI
Nucleic acid metabolism	9.25E-04 - 4.45E-02	6	ABCC4, CXCL3, ITGA2, LPAR4, P2RY12, THBS1

(B): Top molecular and cellular functions of differentially upregulated genes in non-engrafted G1 cells.			
Name	p-value	#	Molecules
Cellular growth and proliferation	3.68E-05 - 1.31E-02	18	AR, BCL2, BLM, DOCK4, ELF3, FANCD2, FGF13, FYB, GNRHR, HAVCR2, HMMR, IGF1, IL2, IRX3, MYCL1, SLC12A2, THBS2, TUBB3
Cell cycle	1.27E-04 - 1.31E-02	11	AR, BCL2, BLM, C11ORF82, CHAF1A, CHAF1B, FANCD2, HAVCR2, IGF1, IL2, TOP2A,
Cellular assembly and organization	1.44E-04 - 1.31E-02	11	BCL2, BLM, CD48, CHF1A, CHF1B, CHL1, IGF1, IL2, MTMR2, NEURL, THBS2
DNA replication, recombination, and repair	1.44E-04 - 6.56E-03	8	AR, BLM, CHAF1A, CHAF1B, IGF1, IL2, THBS2, TOP2A
Gene expression	2.50E-04 - 1.31E-02	7	AR, BCL2, ELF3, GNRHR, IGF1, IL2, SRCAP

**Table 2:** Nine genes with “embryonic development function” that were upregulated in G0 cells.

		Relative Expression G0/G1		
GENE	Gene Name	UCB	BM	MPB
ADAMTS1	ADAM metallopeptidase with thrombospondin type 1 motif	1.78	5.2	9.18
THBS1	Thrombospondin 1	1.64	2.1	2.69
TIMP3	TIMP metallopeptidase inhibitor 3	1.34	2.78	2.16
PTGS1	Prostaglandin-endoperoxide synthase 1, transcript variant 1	1.39	5.6	4.53
NCKAP1	NCK-associated protein, transcript variant 2	1.0	2.67	4.38
EVI1	Ecotropic viral integration site 1	1.0	20.83	2.72
MFGE8	Milk fat globule-EGF factor 8 protein	1.79	3.0	2.1
ITGA2	Integrin, alpha 2 (CD49B, alpha 2 subunit of VLA-2 receptor)	1.5	17.76	4.12
ENST00000353442	Limb region 1 homolog (LMBR1)	1.68	3.6	2.58

### Comparison of target genes with other published data

We compared our present data to those previously reported by Ivanova et al [25] in which a molecular signature of stem cells was described. Among the 484 target genes identified in our studies, the annotation and function of 341 genes are known. Compared to published database [25], 57 of the 341 genes were identified as common HSC specific genes (Table S2A). These 57 genes were described among many of the categories of hematopoietic cells described by Ivanova et al [25] with 26 of the 57 genes expressed in the 3 categories of HSC, long term-HSC and short term-HSC. Interestingly, our analysis identified 14 genes that Ivanova et al [25] reported as genes expressed in stem cells from multiple tissues including HSC, ESC and NSC (Table S2B).

### Proteomic analysis

Mass spectrometry analyses of the same 6 groups of cells yielded 646 protein identities present in all samples. Based on the definition of Higgs et al [32] all the quantified proteins were given priorities 1, 2, 3, or 4 based on the quality of protein identification (see Table S3 for further details). The gene ontology analysis of these proteins is shown in Table 3. The major cellular components (corresponding number of identified proteins in brackets) were membrane-bound organelle (269), nucleus (194), cytosol (74), cytoskeleton (63), and mitochondrion (48). Biological processes with the largest number of identified proteins were nucleic acid metabolism (161), gene expression (154), development (114), transport (97), and cell differentiation (72). This analysis revealed proteins that do not represent the major pathways and cellular compartments identified by microarray analysis.

**Table 3:** Total identified proteins were grouped on the basis of cellular localization, biological process, and molecular function.

Cellular Component	#	%	Biological process	#	%	Molecular function	#	%
Membrane-bound organelle	269	50.6	Nucleic acid metabolic process	161	30.3	Protein binding	298	56
Nucleus	194	36.5	Gene expression	154	29	DNA binding	92	17.3
Cytosol	74	13.9	Protein metabolic process	138	25.9	RNA binding	89	16.7
Cytoskeleton	63	11.8	Cellular macromolecule metabolic process	134	25.2	Structural molecule activity	77	14.5
Mitochondrion	48	9	Developmental process	114	21.4	ATP binding	56	10.5
Chromosome	46	8.7	Transport	97	18.2	Oxidoreductase activity	42	7.9
Ribosome	42	7.9	Cell differentiation	72	13.5	Hydrolase activity	41	7.7
Chromatin	36	6.8	Macromolecule biosynthetic process	64	12	Pyrophosphatase activity	41	7.7
Organelle envelope	31	5.8	DNA metabolic process	60	11.3	ATPase activity	23	4.3
Actin cytoskeleton	26	4.9	Translation	57	10.7	Unfolded protein binding	22	4.1
Nucleosome	24	4.5	RNA processing	43	8.1	Actin binding	22	4.1
Cytoplasmic vesicle	23	4.3	Programmed cell death	40	7.5	GTP binding	20	3.8
Spliceosome	21	4	Cell cycle	40	7.5	GTPase activity	16	3
Microtubule	13	2.4	Chromosome organization	39	7.3	Enzyme inhibitor activity	14	2.6
Cell surface	12	2.3	mRNA processing	34	6.4	Isomerase activity	12	2.3
Nuclear envelope	12	2.3	RNA splicing	32	6	Structural constituent of cytoskeleton	12	2.3
Nucleolus	12	2.3	Chromatin assembly or disassembly	30	5.6	Protein domain specific binding	11	2.1
Contractile fiber	11	2.1	Anti-apoptosis	18	3.4	Lyase activity	11	2.1
Myosin complex	7	1.3	DNA repair	15	2.8	Antioxidant activity	10	1.9
Hetero Chromatin	5	0.9	Coenzyme metabolic process	15	2.8	Chromatin binding	8	1.5

### Differential expression of proteins between G0 and G1

To gain insight into the mechanism of engraftment at the protein level, we examined protein differential expression in G0 and G1 cells from all three tissues. Differential expression of proteins was measured from the largest to the smallest protein intensity between groups. A significant fold change was based on controlling the false discovery rate (FDR) at less than 5. The relative expression of all proteins with individual standard error charts are shown in Table S4. When the threshold was set to 1.5, 25 proteins ranked in priorities 1 and 2 from BM were differentially expressed between G0 and G1. For the same ranking, 12 proteins from MPB, and 22 proteins from UCB were differentially expressed between G0 and G1 cells. Table S5 displays the list of differentially expressed proteins along with their annotation, the sequence used to identify the protein, and fold change. There were only 7 differentially expressed proteins common in all three tissues. To identify target proteins associated with engraftment, analysis similar to that carried out for the genomic data was performed. Using the same assumptions discussed above, we identified 11 common proteins differentially expressed by BM and MPB. Only 4 proteins however, were commonly differentially expressed between BM and MPB, but not UCB (Table S5).

## Discussion

In adults, the quiescent status of HSC is believed to be a critical determinant in the ability of these cells to retain their full hematopoietic potential [36], [37]. We previously hypothesized [6] that in the developing fetus, and in order to meet the extensive demand for the production of hematopoietic cells, all CD34^+^ cells, regardless of their position in the cell cycle, can sustain and reinitiate blood cell production as hematopoiesis moves from one site to the other during fetal development. Using a series of transplantation studies [4], [5], [6] we demonstrated that only G0 CD34^+^ cells from adult human BM or MPB engrafted successfully in conditioned NOD/SCID mice and as predicted, both G0CD34^+^ and G1CD34^+^ cells from UCB, fetal liver, and fetal BM engrafted effectively [4], [5], [6]. While these studies revealed the role of cell cycle status in the engraftment of CD34^+^ cells during ontogeny, the molecular basis behind these observations remains unknown. Furthermore, these studies suggested that perhaps genes differentially expressed between UCB G0CD34^+^ and G1CD34^+^ cells, especially those involved in cell cycle control may not be critical for conferring engraftment capabilities. In this study, we relied on previously published findings and the rational of differential gene expression between G0CD34^+^ and G1CD34^+^ cells from different tissues to derive a genetic and protein fingerprint that may be associated with the engraftment potential of human stem cells and to examine whether our data can explain the engraftment of cells in G0 based on their coordinated and position in a continuum rather than a property that is strictly cell cycle associated mitotically and genetically.

In our analysis, genes with at least two fold change and p-value<0.01 were considered differentially expressed. Only 159 differentially expressed genes were common to all three tissues. Regardless of engraftment potential, several genes undergo differential expression when cells migrate from mitotic quiescence (G0) to active phases of cell cycle (G1). Since we used CD34^+^ cells from 3 different tissues with distinct engraftment potential, we were able to subtract genes that were differentially expressed merely due to cell cycle progression and focus on engraftment related genes only. Nine genes, ADAMTS1, THBS1, TIMP3, PTGS1, NCKAP1, EVI1, MFGE8, ITGA2, ENST00000353442, with embryonic development function were upregulated in engrafted cells. A number of these genes have an already identified role in maintaining hematopoietic stem cells directly (EVI1) or indirectly (ENST00000353442) by altering the expression of genes implicated in the maintenance of stem cell function such as sonic hedgehog [38]. Many of these genes play critical roles in embryonic differentiation, implantation, and tissue homeostasis (PTSG1) [39], [40], in embryonic body morphogenesis and gastrulation (NCKAP1) [41], and in organ morphogenesis (ITGA2) [42] and limb patterning (ENST00000353442) [38]. How these genes collectively participate in controlling hematopoietic stem cell engraftment remains to be fully elucidated.

Interestingly, we found that the expression of several target genes upregulated in engrafted cells can be inversely affected by the expression of genes that were upregulated in non-engrafted cells (Figure 3). For instance, growth arrest and DNA-damage-inducible, alpha (GADD45A), an essential component of many metabolic pathways that control proliferating cancer cells [43] had relatively high expression levels in engrafted cells. B-cell CLL/lymphoma 2 (BCL2) protein which was highly expressed in non-engrafted cells has been previously shown to suppress the expression of human GADD45A protein [44]. Whether over expression of BCL2 in non-engrafted cells negatively regulates the expression of GADD45A thereby promoting a loss of engraftment potential requires closer examination. Similarly, expression of thrombospondin1 (THBS1) which has a role in the activation of MAPK [45], anti-apoptosis [46], and cell cycle arrest [47] was upregulated in engrafted cells. THBS1 protein decreases the secretion of IL2 [48], which, as noted above and in Figure 3, is associated with non-engrafting cells. Integrin, alpha 2 (ITGA2 or CD49B) which is involved in cell adhesion and cell-surface mediated signaling [49] was upregulated in engrafted cells while the androgen receptor (AR or dihydrotestosterone receptor) was overexpressed in non-engrafting cells. Interestingly, 5alpha-dihydrotestosterone has been previously shown to decrease the expression of ITGA2 [50]. It would have been very interesting and informative if we could have extended these analyses to cells in S/G+M phases of cell cycle. Unfortunately, only a very small percentage of UCB and MPB-derived CD34^+^ cells are in S/G2+M [4], [6], making the isolation of sufficient numbers of these cells extremely difficult.

These observations suggest that maintenance and loss of engraftment potential may be controlled by an orchestrated sequence of gene expression profiles that oscillate HSC between engrafting and non-engrafting potential. It would be interesting to closely examine whether these gene expression profiles are modulated by the progression of cells through different phases of cell cycle. Such associations between cell cycle status and a genetic fingerprint that promotes engraftment may explain why position of HSC in cell cycle is an important parameter in determining their engraftment potential. Alternating status of engraftment potential of HSC has been previously reported by Quesenberry and colleagues [7], [8], [51], [52], [53]. It is important to stress here that restriction of engraftment to cells in G0 may not be applicable to unseparated BM cells. However, the necessity to use purified cells for genomic and proteomic analyses, preclude the use of unseparated cells for these studies.

We compared our 484 target genes identified by microarray analysis to the published stem cell database where Ivanova et al. [25] mapped mRNA expression profiles of hematopoietic stem cells of various phenotypically defined hierarchical levels or clusters including LT-HSC, ST-HSC, and early-intermediate-late progenitors. Among the functionally annotated 341 genes identified in our set, 57, which mapped to all clusters of hematopoietic cells examined, were present in the database of Ivanova et al. Hypothetical expression patterns used by Ivanova et al., [25] for cluster assignment may be one of the reasons for our target list matching to all clusters. It is important to note that when we compared our target gene list to common genes expressed by different types of stem cells (HSC, ESC, and NSC as per the definition of Ivanova et al), 14 genes were common to both lists and interestingly, all of them mapped to higher clusters of HSC developmental hierarchy.

Using mass spectrometry based proteomic analysis we successfully identified 646 proteins from the same 18 groups of cells that were subjected in parallel to microarray analysis. Analysis strategies similar to those used with our microarray data revealed that only 4 common proteins were differentially expressed in both BM and MPB, and not changed in UCB. We consider this a huge constraint of our proteomic analysis since compared to data from microarrays. We were limited to the identification of a rather small number of expressed proteins using currently available techniques. Given the logistical difficulties involving the flow cytometric cell sorting of highly purified phenotypically defined groups of cells, and the decision not to mix samples, we were only able to use a rather small number of sorted cells (2×10^5^ cells) for the proteomic analysis of each sample. This may have contributed significantly to why only few proteins were identified in our analysis. It is also important to note that another possible reason for our inability to identify a larger number of proteins is that cells in our samples are mostly metabolically inactive thus limiting the proteome abundance. The use of independent pooled samples with significantly higher numbers of cells may have generated a more robust set of proteomic data and showed a higher synergy between the transcriptome and microarray analyses of these cells.

We found a poor correlation between microarray and proteomic results. For instance, among the 62 differentially expressed proteins between BM G0 and G1 cells (Table S5), only 10 matched to differentially expressed genes by ID matching (ignoring hypothetical- putative proteins, that could not be matched to microarray gene ID). Such discrepancies between proteomic and microarray data were previously reported [13], [14], [15], [16]. For example, Gygi et al. detected a 20 fold-increase in protein expression for some genes that were unaltered at the mRNA level by microarray analysis [16], [54]. Clearly, a twofold differential expression of genes may not necessarily result in a twofold change in protein expression [55]. In addition, several mRNA molecules are not translated and may act as transcription regulators or may decay before protein is synthesized [56], [57] leading to a differential expression of genes, but not proteins. Therefore, thresholds applied to identify differential expression of genes and proteins may not reflect the *in vivo* status of transcription and translation.

Our studies confirm what several investigators in this field have suspected for a long time, namely that it is not the mitotic quiescence of HSC per se that is responsible for the superior functional capabilities of these cells but instead, the genomic status associated with or resulting from their position in cell cycle. We purposefully adopted an analysis strategy that stripped away differences between UCB-derived G0 and G1 cells thus eliminating any genomic difference that may be attributed to the cell cycle status of these functionally similar groups of cells. This approach revealed the presence of non cell cycle related genes contributing to the engrafting potential of putative HSC that matched genes identified by other microarray analysis strategies [25]. Obviously, our results cannot rule out that cell cycle related genes do play a role in conferring functional prowess to mitotically inactive cells. Another intriguing finding in these studies is the detection of genes in one of the two fractions analyzed from each tissue capable of modulating the expression of other genes expressed in the opposing fraction. These data suggest that a Yin and Yang system of gene expression patterns in HSC may exist allowing for the oscillation of cells, perhaps in a cell cycle related fashion, between engrafting and non-engrafting status thus generating a continuum of functions from within what would otherwise appear as a heterogeneous population of cells. This model of cell cycle related stem cell function and the oscillation between possessing and losing functional properties within the same cell population was previously described experimentally [51] and proposed conceptually by several groups for adult [7], [8], [9], [10] and embryonic cells [58]. Interestingly, this model of stem cell flexibility allows for more than one group of cells within a given hematopoietic tissue to possess basic and essential stem cell properties when needed. Such would be the case for fetal hematopoietic cells which engraft regardless of their position in cell cycle probably because of the high requirement for hematopoietic cells during development and the continued migration of hematopoiesis from one hematopoietic site to another during ontogeny.

## Supporting Information

Figure S1 **Phenotypic analysis of UCB and BM cell isolated in G0 or G1 phases of cell cycle.** Each group of sorted cells was stained with the 10 hematopoietic markers listed next to each row of histograms (FITC-conjugated) and analyzed separately. Grey histogram denotes isotype control and green histogram denotes test sample.
(TIF)

Table S1 **The list of target genes identified by microarray analysis.**
(XLS)

Table S2 **Comparison of target genes with published database.** (**A**): Common genes found between microarray identified target genes and published hematopoietic stem cell database [25]. (**B**): Common genes found between the microarray identified target genes and the stem cell genes that are conserved and found common among HSC, ESC, and NSC.
(DOC)

Table S3 **The list of total proteins identified by liquid chromatography - mass spectrometry (LC/MS).**
(XLS)

Table S4 **Variability charts of identified total proteins differential expression, including unchanged proteins expression.** For each protein, a plot of the group mean protein intensity levels on the log base 2 scale plus or minus the standard error is shown. The standard error is computed from the statistical model and is a measure of the precision of the mean.
(DOC)

Table S5 **Differentially expressed proteins between G0 and G1 cells of BM, MPB, and UCB.** (**A**): Differentially expressed proteins between G0 and G1 cells of BM. (**B**): Differentially expressed proteins between G0 and G1 cells of MPB. (**C**): Differentially expressed proteins between G0 and G1 cells of UCB.
(DOC)
