# Supplementary figures and images for: Genomic and Proteomic Analysis of the Impact of Mitotic Quiescence on the Engraftment of Human CD34+ Cells
Source: PLoS One. 2011 Mar 7;6(3):e17498. doi: 10.1371/journal.pone.0017498 (PMC3049784; doi:10.1371/journal.pone.0017498)

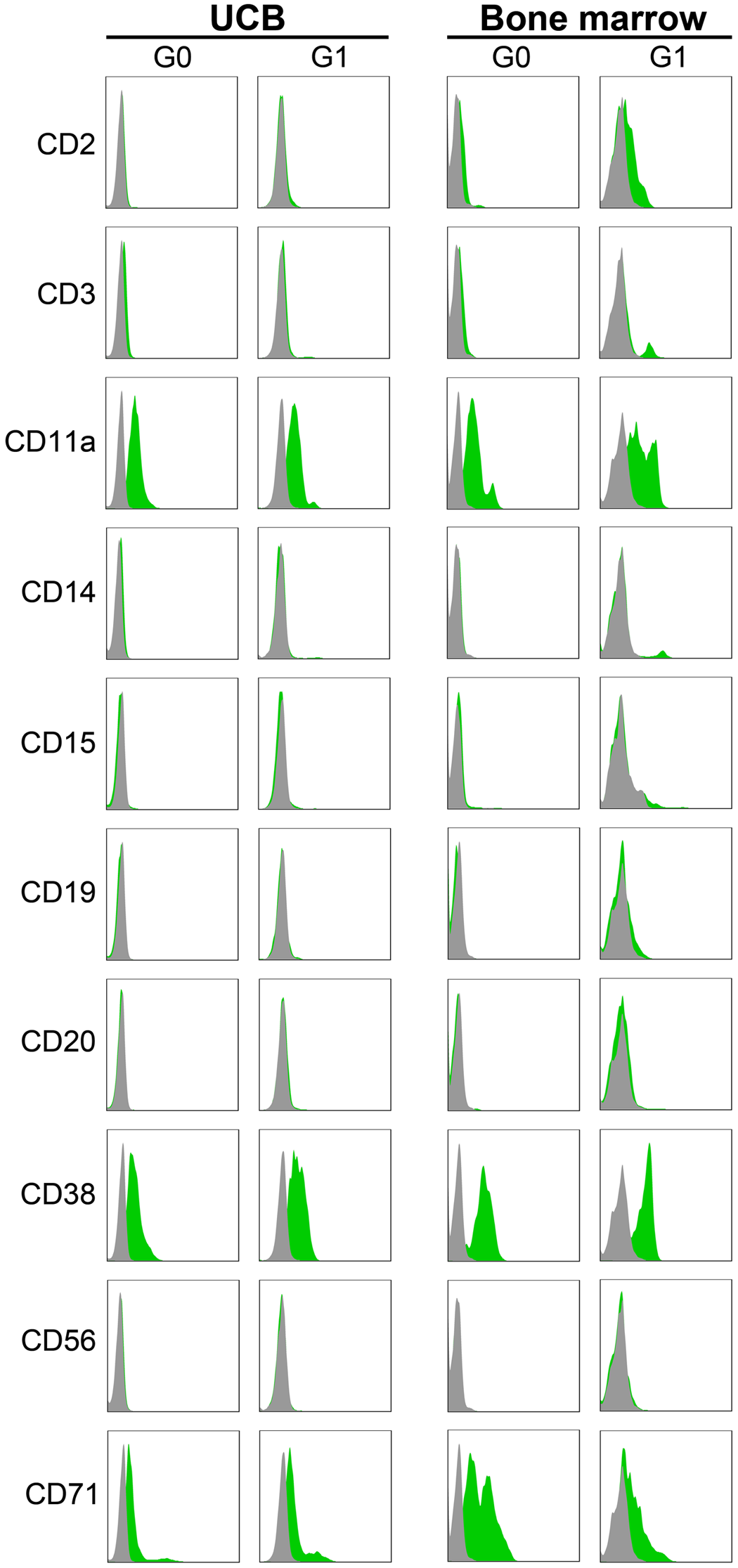

Supplement: Figure S1 — Phenotypic analysis of UCB and BM cell isolated in G0 or G1 phases of cell cycle. Each group of sorted cells was stained with the 10 hematopoietic markers listed next to each row of histograms (FITC-conjugated) and analyzed separately. Grey histogram denotes isotype control and green histogram denotes test sample. (TIF) [file pone.0017498.s001.tif]
