# Supplementary material for: Genomic and Proteomic Analysis of the Impact of Mitotic Quiescence on the Engraftment of Human CD34+ Cells
Source: PLoS One. 2011 Mar 7;6(3):e17498. doi: 10.1371/journal.pone.0017498 (PMC3049784; doi:10.1371/journal.pone.0017498)
Supplement: Table S2 — Comparison of target genes with published database. (A): Common genes found between microarray identified target genes and published hematopoietic stem cell database [25]. (B): Common genes found between the microarray identified target genes and the stem cell genes that are conserved and found common among HSC, ESC, and NSC. (DOC) [file pone.0017498.s003.doc]

**Table S2.** Comparison of target genes with published database.

**(A):** Common genes found between microarray identified target genes and published hematopoietic stem cell database.

| **SN** | **Gene Name** | **Human GenBank ID**  **(Our MA data)** | **Mouse Affimetrix ID**  **(Ivanova et al.)** | **Gene annotation** | **Expression cluster** |
| --- | --- | --- | --- | --- | --- |
| 1 | ABCC4 | NM_005845 | 111137_AT | ATP-binding cassette, sub-family C (CFTR/MRP), member 4 | Early progenitors |
| 2 | ACOT2 | CR606809 | 163689_AT | Acyl-coA thioesterase 2 | HSC and progenitors |
| 3 | ADARB1 | AY082339 | 161436_S_AT | Adenosine deaminase, RNA-specific, B1 (RED1 homolog) | LT-HSC |
| 4 | ANXA3 | NM_005139 | 101393_AT | Annexin A3 | Late progenitors |
| 5 | CHN2 | NM_004067 | 163407_AT | Chimerin (chimaerin) 2 | Early progenitors |
| 6 | FCER1A | NM_002001 | 101209_AT | Fc fragment of IGE, high affinity I, receptor for; alpha polypeptide | Late progenitors |
| 7 | GADD45A | NM_001924 | 102292_AT | Growth arrest and DNA-damage-inducible, alpha | MBC |
| 8 | GPR23 | NM_005296 | 114749_AT | G protein-coupled receptor 23 | HSC |
| 9 | KIF21A | NM_017641 | 166472_I_AT | Kinesin family member 21a | Early progenitors |
| 10 | PBX1 | AA594975 | 105387_AT | Pre-B-cell leukemia transcription factor 1 | MBC |
| 11 | PRDM16 | NM_199454 | 167461_R_AT | PR domain containing 16 | LT-HSC |
| 12 | RPS6 | NM_001010 | 169270_AT | Ribosomal protein S6 | HSC and progenitors |
| 13 | RSRC1 | NM_016625 | 105533_AT | Arginine/serine-rich coiled-coil 1 | Early progenitors |
| 14 | SLC25A21 | AK057476 | 116760_AT | Oxodicarboxylate carrier | Late progenitors |
| 15 | SLC40A1 | NM_014585 | 128795_AT | Solute carrier family 40 (iron-regulated transporter), member 1 | MBC |
| 16 | SORT1 | NM_002959 | 163108_AT | Sortilin 1 | MBC |
| 17 | SYTL4 | NM_080737 | 161026_S_AT | Synaptotagmin-like 4 (granuphilin-A) | HSC and progenitors |
| 18 | TACC1 | NM_006283 | 112375_AT | Transforming, acidic coiled-coil containing protein 1 | Late progenitors |
| 19 | TEX2 | NM_018469 | 108108_AT | Testis expressed sequence 2 | Intermediate progenitors |
| 20 | THBS1 | NM_003246 | 160469_AT | Thrombospondin 1 | MBC |
| 21 | TSPAN6 | NM_003270 | 92555_AT | Tetraspanin 6 | HSC |
| 22 | B3GNT1 | NM_006876 | 171549_AT | UDP-GLCNAC:betagal beta-1,3-N-acetylglucosaminyltransferase 6 | LT-HSC |
| 23 | BCL2 | NM_000633 | 98869_G_AT | B-cell CLL/lymphoma 2 | HSC and progenitors |
| 24 | CHL1 | NM_006614 | 169984_I_AT | Cell adhesion molecule with homology to L1CAM (close homolog of l1) | Late progenitors |

**Table S2.** Continued.

| **SN** | **Gene Name** | **Human GenBank ID**  **(Our MA data)** | **Mouse Affimetrix ID**  **(Ivanova et al.)** | **Gene annotation** | **Expression cluster** |
| --- | --- | --- | --- | --- | --- |
| 25 | COMMD8 | NM_017845 | 110702_AT | COMM domain containing 8 | MBC |
| 26 | CST7 | NM_003650 | 102638_AT | Cystatin F (leukocystatin) | Early progenitors |
| 27 | DNTT | NM_004088 | 103961_S_AT | Deoxynucleotidyltransferase, terminal | ST-HSC |
| 28 | FYB | NM_001465 | 110738_AT | FYN binding protein (FYB-120/130) | MBC |
| 29 | HERPUD2 | BC049371 | 167651_S_AT | Hypothetical protein FLJ22313 | MBC |
| 30 | IFT81 | NM_031473 | 102215_AT | Intraflagellar transport 81 homolog (chlamydomonas) | HSC |
| 31 | MYCL1 | NM_005376 | 102235_AT | V-myc myelocytomatosis viral oncogene homolog 1, lung carcinoma derived | LT-HSC |
| 32 | NEURL | NM_004210 | 134135_R_AT | Neuralized-like (Drosophila) | Late progenitors |
| 33 | OLFML2A | NM_182487 | 116470_AT | Olfactomedin-like 2a | LT-HSC |
| 34 | PTPN14 | NM_005401 | 98385_AT | Protein tyrosine phosphatase, non-receptor type 14 | LT-HSC |
| 35 | RAB32 | NM_006834 | 113750_AT | Rab32, member ras oncogene family | Late progenitors |
| 36 | SDC1 | BI058698 | 161370_F_AT | Syndecan 1 | HSC |
| 37 | SLC12A2 | NM_001046 | 99500_AT | Solute carrier family 12 (sodium/potassium/chloride transporters), member 2 | LT-HSC |
| 38 | SLC29A1 | NM_004955 | 95733_AT | Solute carrier family 29 (nucleoside transporters), member 1 | Late progenitors |
| 39 | SLC2A4 | NM_001042 | 162385_I_AT | Solute carrier family 2 (facilitated glucose transporter), member 4 | MBC |
| 40 | SLC7A8 | NM_182728 | 104214_AT | Solute carrier family 7 (cationic amino acid transporter, y+ system), member 8 | MBC |
| 41 | STEAP1 | NM_012449 | 164240_AT | Six transmembrane epithelial antigen of the prostate 1 | LT-HSC |
| 42 | SVOP | NM_018711 | 112505_AT | SV2 related protein homolog | MBC |
| 43 | CD93 | NM_012072 | 93454_AT | CD93 antigen | HSC and progenitors |
| 44 | MTFR1 | NM_014637 | 162793_AT | Mitochondrial fission regulator 1 | Late progenitors |
| 45 | ODF2L | AB033055 | 160984_R_AT | Outer dense fiber of sperm tails 2-like | Early progenitors |

**Table S2.** Continued.

| **SN** | **Gene Name** | **Human GenBank ID**  **(Our MA data)** | **Mouse Affimetrix ID**  **(Ivanova et al.)** | **Gene annotation** | **Expression cluster** |
| --- | --- | --- | --- | --- | --- |
| 46 | RASAL2 | NM_170692 | 116809_AT | Ras protein activator like 2 | LT-HSC |
| 47 | SLC25A37 | NM_016612 | 163354_AT | Solute carrier family 25, member 37 | MBC |
| 48 | YIPF5 | BM983766 | 164815_I_AT | YIP1 domain family, member 5 | HSC and progenitors |
| 49 | AYTL2 | AK090444 | 116361_AT | Acyltransferase like 2 | MBC |
| 50 | KIF5A | NM_004984 | 162987_AT | Kinesin family member 5a | HSC |
| 51 | PPIC | NM_000943 | 100089_AT | Peptidylprolyl isomerase C (cyclophilin C) | HSC and progenitors |
| 52 | PXT1 | NM_152990 | 168576_I_AT | Peroxisomal, testis specific 1 | LT-HSC |
| 53 | RPS3 | NM_001005 | 169419_I_AT | Ribosomal protein s3 | LT-HSC |
| 54 | SCIN | NM_033128 | 103715_AT | Scinderin | Intermediate progenitors |
| 55 | SEC24D | AK000709 | 109669_AT | SEC24 related gene family, member D | Early progenitors |
| 56 | SPTY2D1 | AK097654 | 109388_R_AT | Spt2, suppressor of TY, domain containing 1 | HSC |
| 57 | WWOX | AK125176 | 170782_R_AT | Putative oxidoreductase | HSC and progenitors |

**(B):** Common genes found between the microarray identified target genes and the stem cell genes that are conserved and found common among HSC, ESC, and NSC.

| **SN** | **Gene Name** | **Human GenBank ID**  **(Our MA data)** | **Mouse Affimetrix ID**  **(Ivanova et al.)** | **Gene annotation** | **Expression cluster** |
| --- | --- | --- | --- | --- | --- |
| 1 | WWOX | AK125176 | 170782_R_AT | Putative oxidoreductase | HSC and progenitors |
| 2 | ACOT2 | CR606809 | 163689_AT | Acyl-coA thioesterase 2 | HSC and progenitors |
| 3 | PPIC | NM_000943 | 100089_AT | Peptidylprolyl isomerase C (cyclophilin C) | HSC and progenitors |
| 4 | YIPF5 | BM983766 | 164815_I_AT | YIP1 domain family, member 5 | HSC and progenitors |
| 5 | GPR23 | NM_005296 | 114749_AT | G protein-coupled receptor 23 | HSC |
| 6 | KIF5A | NM_004984 | 162987_AT | Kinesin family member 5a | HSC |
| 7 | SDC1 | BI058698 | 161370_F_AT | Syndecan 1 | HSC |
| 8 | TSPAN6 | NM_003270 | 92555_AT | Tetraspanin 6 | HSC |
| 9 | MYCL1 | NM_005376 | 102235_AT | V-myc myelocytomatosis viral oncogene homolog 1, lung carcinoma derived | LT-HSC |
| 10 | STEAP1 | NM_012449 | 164240_AT | Six transmembrane epithelial antigen of the prostate 1 | LT-HSC |
| 11 | ADARB1 | AY082339 | 161436_S_AT | Adenosine deaminase, RNA-specific, B1 (RED1 homolog rat) | LT-HSC |
| 12 | RASAL2 | NM_170692 | 116809_AT | Ras protein activator like 2 | LT-HSC |
| 13 | RPS3 | NM_001005 | 169419_I_AT | Ribosomal protein s3 | LT-HSC |
| 14 | CHN2 | NM_004067 | 163407_AT | Chimerin (chimaerin) 2 | ST-HSC |
