# Supplementary material for: Genomic and Proteomic Analysis of the Impact of Mitotic Quiescence on the Engraftment of Human CD34+ Cells
Source: PLoS One. 2011 Mar 7;6(3):e17498. doi: 10.1371/journal.pone.0017498 (PMC3049784; doi:10.1371/journal.pone.0017498)
Supplement: Table S4 — Variability charts of identified total proteins differential expression, including unchanged proteins expression. For each protein, a plot of the group mean protein intensity levels on the log base 2 scale plus or minus the standard error is shown. The standard error is computed from the statistical model and is a measure of the precision of the mean. (DOC) [file pone.0017498.s005.doc]

**Table S4.** Variability charts of identified total proteins differential expression, including unchanged proteins expression. For each protein, a plot of the group mean protein intensity levels on the log base 2 scale plus or minus the standard error is shown. The standard error is computed from the statistical model and is a measure of the precision of the mean.

**Variability Gauge Rank=1, protein_id=48734733, Annotation=Eukaryotic_translation_elongation_factor_1_alpha_1_[Homo_sapiens]**

**Variability Chart for Mean Log2(Intensity) +/- StdErr**

**Variability Gauge Rank=2, protein_id=IPI00025447.6, Annotation=EEF1A1_protein**

**Variability Chart for Mean Log2(Intensity) +/- StdErr**

**Variability Gauge Rank=3, protein_id=927065, Annotation=eukaryotic_translation_elongation_factor_1_alpha_1-like_14_[Homo_sapiens]**

**Variability Chart for Mean Log2(Intensity) +/- StdErr**

**Variability Gauge Rank=4, protein_id=IPI00007244.1, Annotation=Isoform_H17_of_Myeloperoxidase_precursor**

**Variability Chart for Mean Log2(Intensity) +/- StdErr**

**Variability Gauge Rank=5, protein_id=IPI00218918.4, Annotation=Annexin_A1**

**Variability Chart for Mean Log2(Intensity) +/- StdErr**

**Variability Gauge Rank=6, protein_id=IPI00006690.1, Annotation=Eosinophil_peroxidase_precursor**

**Variability Chart for Mean Log2(Intensity) +/- StdErr**

**Variability Gauge Rank=7, protein_id=188590, Annotation=myosin_light_chain_3**

**Variability Chart for Mean Log2(Intensity) +/- StdErr**

**Variability Gauge Rank=8, protein_id=IPI00014424.1, Annotation=Elongation_factor_1-alpha_2**

**Variability Chart for Mean Log2(Intensity) +/- StdErr**

**Variability Gauge Rank=9, protein_id=IPI00335168.8, Annotation=myosin,_light_polypeptide_6,_alkali,_smooth_muscle_and_non-muscle_isoform_1**

**Variability Chart for Mean Log2(Intensity) +/- StdErr**

**Variability Gauge Rank=10, protein_id=IPI00418471.5, Annotation=Vimentin**

**Variability Chart for Mean Log2(Intensity) +/- StdErr**

**Variability Gauge Rank=11, protein_id=IPI00025849.1, Annotation=Acidic_leucine-rich_nuclear_phosphoprotein_32_family_member_A**

**Variability Chart for Mean Log2(Intensity) +/- StdErr**

**Variability Gauge Rank=12, protein_id=IPI00020599.1, Annotation=Calreticulin_precursor**

**Variability Chart for Mean Log2(Intensity) +/- StdErr**

**Variability Gauge Rank=13, protein_id=IPI00382470.2, Annotation=Heat_shock_protein_HSP_90-alpha_2**

**Variability Chart for Mean Log2(Intensity) +/- StdErr**

**Variability Gauge Rank=14, protein_id=IPI00219156.6, Annotation=60S_ribosomal_protein_L30**

**Variability Chart for Mean Log2(Intensity) +/- StdErr**

**Variability Gauge Rank=15, protein_id=IPI00465028.6, Annotation=Triosephosphate_isomerase**

**Variability Chart for Mean Log2(Intensity) +/- StdErr**

**Variability Gauge Rank=16, protein_id=5822065, Annotation=A_Chain_A,_Superoxide_Dismutase_Mutant_With_Lys_136_Replaced_By_Glu,_Cys_6_Replaced_By_Ala_And_Cys_111_Replaced_By_Ser_(K136e,_C6a,_C111s)**

**Variability Chart for Mean Log2(Intensity) +/- StdErr**

**Variability Gauge Rank=17, protein_id=IPI00013475.1, Annotation=tubulin,_beta_2**

**Variability Chart for Mean Log2(Intensity) +/- StdErr**

**Variability Gauge Rank=18, protein_id=3659957, Annotation=_Chain_,_The_Solution_Structure_Of_Reduced_Monomeric_Superoxide_Dismutase,_Nmr,_36_Structures**

**Variability Chart for Mean Log2(Intensity) +/- StdErr**

**Variability Gauge Rank=19, protein_id=IPI00016610.2, Annotation=Poly(rC)-binding_protein_1**

**Variability Chart for Mean Log2(Intensity) +/- StdErr**

**Variability Gauge Rank=20, protein_id=IPI00217466.2, Annotation=Histone_H1.3**

**Variability Chart for Mean Log2(Intensity) +/- StdErr**

**Variability Gauge Rank=21, protein_id=IPI00784295.1, Annotation=Heat_shock_protein_HSP_90-alpha**

**Variability Chart for Mean Log2(Intensity) +/- StdErr**

**Variability Gauge Rank=22, protein_id=IPI00239077.4, Annotation=Histidine_triad_nucleotide-binding_protein_1**

**Variability Chart for Mean Log2(Intensity) +/- StdErr**

**Variability Gauge Rank=23, protein_id=IPI00011654.2, Annotation=Tubulin_beta-2_chain**

**Variability Chart for Mean Log2(Intensity) +/- StdErr**

**Variability Gauge Rank=24, protein_id=IPI00746213.1, Annotation=7_kDa_protein**

**Variability Chart for Mean Log2(Intensity) +/- StdErr**

**Variability Gauge Rank=25, protein_id=IPI00217465.4, Annotation=Histone_H1.2**

**Variability Chart for Mean Log2(Intensity) +/- StdErr**

**Variability Gauge Rank=26, protein_id=IPI00220834.7, Annotation=ATP-dependent_DNA_helicase_2_subunit_2**

**Variability Chart for Mean Log2(Intensity) +/- StdErr**

**Variability Gauge Rank=27, protein_id=IPI00003935.5, Annotation=Histone_H2B_type_2-E**

**Variability Chart for Mean Log2(Intensity) +/- StdErr**

**Variability Gauge Rank=28, protein_id=IPI00018534.3, Annotation=Histone_H2B_type_1-L**

**Variability Chart for Mean Log2(Intensity) +/- StdErr**

**Variability Gauge Rank=29, protein_id=IPI00554798.1, Annotation=Histone_H2B_type_1-M**

**Variability Chart for Mean Log2(Intensity) +/- StdErr**

**Variability Gauge Rank=30, protein_id=IPI00025491.1, Annotation=Eukaryotic_initiation_factor_4A-I**

**Variability Chart for Mean Log2(Intensity) +/- StdErr**

**Variability Gauge Rank=31, protein_id=IPI00739539.2, Annotation=PREDICTED:_similar_to_Prostate,_ovary,_testis_expressed_protein_on_chromosome_2**

**Variability Chart for Mean Log2(Intensity) +/- StdErr**

**Variability Gauge Rank=32, protein_id=IPI00026138.4, Annotation=25_kDa_protein**

**Variability Chart for Mean Log2(Intensity) +/- StdErr**

**Variability Gauge Rank=33, protein_id=IPI00419833.6, Annotation=Histone_H2B_type_1-K**

**Variability Chart for Mean Log2(Intensity) +/- StdErr**

**Variability Gauge Rank=34, protein_id=IPI00027409.1, Annotation=Myeloblastin_precursor**

**Variability Chart for Mean Log2(Intensity) +/- StdErr**

**Variability Gauge Rank=35, protein_id=IPI00382804.1, Annotation=EEF1A_protein_(Fragment)**

**Variability Chart for Mean Log2(Intensity) +/- StdErr**

**Variability Gauge Rank=36, protein_id=IPI00550363.2, Annotation=Transgelin-2**

**Variability Chart for Mean Log2(Intensity) +/- StdErr**

**Variability Gauge Rank=37, protein_id=IPI00293434.1, Annotation=Signal_recognition_particle_14_kDa_protein**

**Variability Chart for Mean Log2(Intensity) +/- StdErr**

**Variability Gauge Rank=38, protein_id=IPI00007750.1, Annotation=Tubulin_alpha-1_chain**

**Variability Chart for Mean Log2(Intensity) +/- StdErr**

**Variability Gauge Rank=39, protein_id=IPI00440493.2, Annotation=ATP_synthase_subunit_alpha,_mitochondrial_precursor**

**Variability Chart for Mean Log2(Intensity) +/- StdErr**

**Variability Gauge Rank=40, protein_id=IPI00646779.2, Annotation=TUBB6_protein**

**Variability Chart for Mean Log2(Intensity) +/- StdErr**

**Variability Gauge Rank=41, protein_id=IPI00007752.1, Annotation=Tubulin_beta-2C_chain**

**Variability Chart for Mean Log2(Intensity) +/- StdErr**

**Variability Gauge Rank=42, protein_id=IPI00021440.1, Annotation=Actin,_cytoplasmic_2**

**Variability Chart for Mean Log2(Intensity) +/- StdErr**

**Variability Gauge Rank=43, protein_id=IPI00166768.2, Annotation=TUBA6_protein**

**Variability Chart for Mean Log2(Intensity) +/- StdErr**

**Variability Gauge Rank=44, protein_id=IPI00011107.2, Annotation=Isocitrate_dehydrogenase_[NADP],_mitochondrial_precursor**

**Variability Chart for Mean Log2(Intensity) +/- StdErr**

**Variability Gauge Rank=45, protein_id=49456715, Annotation=ALDOA_[Homo_sapiens]**

**Variability Chart for Mean Log2(Intensity) +/- StdErr**

**Variability Gauge Rank=46, protein_id=IPI00217467.2, Annotation=Histone_H1.4**

**Variability Chart for Mean Log2(Intensity) +/- StdErr**

**Variability Gauge Rank=47, protein_id=IPI00021439.1, Annotation=Actin,_cytoplasmic_1**

**Variability Chart for Mean Log2(Intensity) +/- StdErr**

**Variability Gauge Rank=48, protein_id=49457412, Annotation=RPLP1_[Homo_sapiens]**

**Variability Chart for Mean Log2(Intensity) +/- StdErr**

**Variability Gauge Rank=49, protein_id=IPI00008527.3, Annotation=60S_acidic_ribosomal_protein_P1**

**Variability Chart for Mean Log2(Intensity) +/- StdErr**

**Variability Gauge Rank=50, protein_id=IPI00012090.1, Annotation=Cyritestin_2_(Fragment)**

**Variability Chart for Mean Log2(Intensity) +/- StdErr**

**Variability Gauge Rank=51, protein_id=IPI00027255.1, Annotation=Myosin_light_polypeptide_6B**

**Variability Chart for Mean Log2(Intensity) +/- StdErr**

**Variability Gauge Rank=52, protein_id=IPI00020407.1, Annotation=Alpha-1,6-mannosylglycoprotein_6-beta-N-acetylglucosaminyltransferase_V**

**Variability Chart for Mean Log2(Intensity) +/- StdErr**

**Variability Gauge Rank=53, protein_id=IPI00007765.5, Annotation=Stress-70_protein,_mitochondrial_precursor**

**Variability Chart for Mean Log2(Intensity) +/- StdErr**

**Variability Gauge Rank=54, protein_id=IPI00442305.1, Annotation=CDNA_FLJ16182_fis,_clone_BRTHA2002133**

**Variability Chart for Mean Log2(Intensity) +/- StdErr**

**Variability Gauge Rank=55, protein_id=IPI00438271.1, Annotation=Uromodulin-like_1_protein_variant_7_(Fragment)**

**Variability Chart for Mean Log2(Intensity) +/- StdErr**

**Variability Gauge Rank=56, protein_id=IPI00102575.2, Annotation=ATP(GTP)-binding_protein**

**Variability Chart for Mean Log2(Intensity) +/- StdErr**

**Variability Gauge Rank=57, protein_id=IPI00009943.2, Annotation=Tumor_protein,_translationally-controlled_1**

**Variability Chart for Mean Log2(Intensity) +/- StdErr**

**Variability Gauge Rank=58, protein_id=IPI00014830.1, Annotation=Programmed_cell_death_protein_1_precursor**

**Variability Chart for Mean Log2(Intensity) +/- StdErr**

**Variability Gauge Rank=59, protein_id=IPI00008711.3, Annotation=Wolframin**

**Variability Chart for Mean Log2(Intensity) +/- StdErr**

**Variability Gauge Rank=60, protein_id=IPI00025683.2, Annotation=Isoform_1_of_Transforming_acidic_coiled-coil-containing_protein_1**

**Variability Chart for Mean Log2(Intensity) +/- StdErr**

**Variability Gauge Rank=61, protein_id=IPI00061078.5, Annotation=CDNA_FLJ30638_fis,_clone_CTONG2002721,_weakly_similar_to_VACUOLAR_PROTEIN_SORTING-ASSOCIATED_PROTEIN_VPS13.**

**Variability Chart for Mean Log2(Intensity) +/- StdErr**

**Variability Gauge Rank=62, protein_id=IPI00012682.3, Annotation=hypothetical_protein_LOC92196**

**Variability Chart for Mean Log2(Intensity) +/- StdErr**

**Variability Gauge Rank=63, protein_id=IPI00012386.1, Annotation=Cochlin_precursor**

**Variability Chart for Mean Log2(Intensity) +/- StdErr**

**Variability Gauge Rank=64, protein_id=29603474, Annotation=CDC20-like_protein_form_2_[Homo_sapiens]**

**Variability Chart for Mean Log2(Intensity) +/- StdErr**

**Variability Gauge Rank=65, protein_id=IPI00024568.1, Annotation=Glioma_tumor_suppressor_candidate_region_gene_1_protein**

**Variability Chart for Mean Log2(Intensity) +/- StdErr**

**Variability Gauge Rank=66, protein_id=IPI00022043.1, Annotation=Hamartin**

**Variability Chart for Mean Log2(Intensity) +/- StdErr**

**Variability Gauge Rank=67, protein_id=IPI00013297.1, Annotation=28_kDa_heat-_and_acid-stable_phosphoprotein**

**Variability Chart for Mean Log2(Intensity) +/- StdErr**

**Variability Gauge Rank=68, protein_id=IPI00103940.4, Annotation=Tetracycline_transporter-like_protein**

**Variability Chart for Mean Log2(Intensity) +/- StdErr**

**Variability Gauge Rank=69, protein_id=IPI00290272.2, Annotation=DNA_polymerase_subunit_alpha_B**

**Variability Chart for Mean Log2(Intensity) +/- StdErr**

**Variability Gauge Rank=70, protein_id=IPI00383825.2, Annotation=Hypothetical_protein_W80**

**Variability Chart for Mean Log2(Intensity) +/- StdErr**

**Variability Gauge Rank=71, protein_id=27368062, Annotation=class_IVb_beta_tubulin_[Homo_sapiens]**

**Variability Chart for Mean Log2(Intensity) +/- StdErr**

**Variability Gauge Rank=72, protein_id=IPI00399212.2, Annotation=PREDICTED:_similar_to_Ran-specific_GTPase-activating_protein**

**Variability Chart for Mean Log2(Intensity) +/- StdErr**

**Variability Gauge Rank=73, protein_id=IPI00010779.3, Annotation=Tropomyosin_4**

**Variability Chart for Mean Log2(Intensity) +/- StdErr**

**Variability Gauge Rank=74, protein_id=IPI00472102.3, Annotation=Heat_shock_protein_60**

**Variability Chart for Mean Log2(Intensity) +/- StdErr**

**Variability Gauge Rank=75, protein_id=13195586, Annotation=AF351127_1_hemoglobin_alpha_1_globin_chain_[Homo_sapiens]**

**Variability Chart for Mean Log2(Intensity) +/- StdErr**

**Variability Gauge Rank=76, protein_id=IPI00410714.4, Annotation=Hemoglobin_subunit_alpha**

**Variability Chart for Mean Log2(Intensity) +/- StdErr**

**Variability Gauge Rank=77, protein_id=IPI00012066.1, Annotation=poly(rC)-binding_protein_2_isoform_b**

**Variability Chart for Mean Log2(Intensity) +/- StdErr**

**Variability Gauge Rank=78, protein_id=IPI00220827.4, Annotation=Thymosin_beta-10**

**Variability Chart for Mean Log2(Intensity) +/- StdErr**

**Variability Gauge Rank=79, protein_id=IPI00018511.1, Annotation=Tubulin_beta-4q_chain**

**Variability Chart for Mean Log2(Intensity) +/- StdErr**

**Variability Gauge Rank=80, protein_id=223632, Annotation=0904262A_dismutase,Cu/Zn_superoxide**

**Variability Chart for Mean Log2(Intensity) +/- StdErr**

**Variability Gauge Rank=81, protein_id=IPI00019502.2, Annotation=Myosin-9**

**Variability Chart for Mean Log2(Intensity) +/- StdErr**

**Variability Gauge Rank=82, protein_id=IPI00334775.5, Annotation=Hypothetical_protein_DKFZp761K0511**

**Variability Chart for Mean Log2(Intensity) +/- StdErr**

**Variability Gauge Rank=83, protein_id=IPI00059366.3, Annotation=H2A_histone_family,_member_Y_isoform_2**

**Variability Chart for Mean Log2(Intensity) +/- StdErr**

**Variability Gauge Rank=84, protein_id=IPI00219018.6, Annotation=Glyceraldehyde-3-phosphate_dehydrogenase**

**Variability Chart for Mean Log2(Intensity) +/- StdErr**

**Variability Gauge Rank=85, protein_id=126608, Annotation=LYSOZYME_Spiked_Standard_(HEN)**

**Variability Chart for Mean Log2(Intensity) +/- StdErr**

**Variability Gauge Rank=86, protein_id=IPI00007702.1, Annotation=Heat_shock-related_70_kDa_protein_2**

**Variability Chart for Mean Log2(Intensity) +/- StdErr**

**Variability Gauge Rank=87, protein_id=IPI00217468.2, Annotation=Histone_H1.5**

**Variability Chart for Mean Log2(Intensity) +/- StdErr**

**Variability Gauge Rank=88, protein_id=IPI00003865.1, Annotation=Isoform_1_of_Heat_shock_cognate_71_kDa_protein**

**Variability Chart for Mean Log2(Intensity) +/- StdErr**

**Variability Gauge Rank=89, protein_id=IPI00219330.2, Annotation=Isoform_5_of_Interleukin_enhancer-binding_factor_3**

**Variability Chart for Mean Log2(Intensity) +/- StdErr**

**Variability Gauge Rank=90, protein_id=13477319, Annotation=RPL14_protein_[Homo_sapiens]**

**Variability Chart for Mean Log2(Intensity) +/- StdErr**

**Variability Gauge Rank=91, protein_id=IPI00002821.3, Annotation=60S_ribosomal_protein_L14**

**Variability Chart for Mean Log2(Intensity) +/- StdErr**

**Variability Gauge Rank=92, protein_id=IPI00419880.5, Annotation=40S_ribosomal_protein_S3a**

**Variability Chart for Mean Log2(Intensity) +/- StdErr**

**Variability Gauge Rank=93, protein_id=31645, Annotation=glyceraldehyde-3-phosphate_dehydrogenase_[Homo_sapiens]**

**Variability Chart for Mean Log2(Intensity) +/- StdErr**

**Variability Gauge Rank=94, protein_id=IPI00028414.3, Annotation=Glia_maturation_factor_gamma**

**Variability Chart for Mean Log2(Intensity) +/- StdErr**

**Variability Gauge Rank=95, protein_id=IPI00334587.1, Annotation=Isoform_2_of_Heterogeneous_nuclear_ribonucleoprotein_A/B**

**Variability Chart for Mean Log2(Intensity) +/- StdErr**

**Variability Gauge Rank=96, protein_id=IPI00465436.3, Annotation=Catalase**

**Variability Chart for Mean Log2(Intensity) +/- StdErr**

**Variability Gauge Rank=97, protein_id=178045, Annotation=gamma-actin**

**Variability Chart for Mean Log2(Intensity) +/- StdErr**

**Variability Gauge Rank=98, protein_id=IPI00219757.12, Annotation=Glutathione_S-transferase_P**

**Variability Chart for Mean Log2(Intensity) +/- StdErr**

**Variability Gauge Rank=99, protein_id=IPI00374732.4, Annotation=PREDICTED:_similar_to_peptidylprolyl_isomerase_A_isoform_1**

**Variability Chart for Mean Log2(Intensity) +/- StdErr**

**Variability Gauge Rank=100, protein_id=IPI00296291.2, Annotation=HP1-BP74**

**Variability Chart for Mean Log2(Intensity) +/- StdErr**

**Variability Gauge Rank=101, protein_id=IPI00014165.1, Annotation=Unknown_protein**

**Variability Chart for Mean Log2(Intensity) +/- StdErr**

**Variability Gauge Rank=102, protein_id=IPI00003817.2, Annotation=Rho_GDP-dissociation_inhibitor_2**

**Variability Chart for Mean Log2(Intensity) +/- StdErr**

**Variability Gauge Rank=103, protein_id=IPI00329389.6, Annotation=DNA-binding_protein_TAXREB107**

**Variability Chart for Mean Log2(Intensity) +/- StdErr**

**Variability Gauge Rank=104, protein_id=IPI00465439.4, Annotation=Fructose-bisphosphate_aldolase_A**

**Variability Chart for Mean Log2(Intensity) +/- StdErr**

**Variability Gauge Rank=105, protein_id=IPI00418262.3, Annotation=Fructose-bisphosphate_aldolase_C**

**Variability Chart for Mean Log2(Intensity) +/- StdErr**

**Variability Gauge Rank=106, protein_id=IPI00179330.6, Annotation=ubiquitin_and_ribosomal_protein_S27a_precursor**

**Variability Chart for Mean Log2(Intensity) +/- StdErr**

**Variability Gauge Rank=107, protein_id=IPI00216049.1, Annotation=Isoform_1_of_Heterogeneous_nuclear_ribonucleoprotein_K**

**Variability Chart for Mean Log2(Intensity) +/- StdErr**

**Variability Gauge Rank=108, protein_id=IPI00643920.2, Annotation=Transketolase**

**Variability Chart for Mean Log2(Intensity) +/- StdErr**

**Variability Gauge Rank=109, protein_id=IPI00216592.2, Annotation=Isoform_C1_of_Heterogeneous_nuclear_ribonucleoproteins_C1/C2**

**Variability Chart for Mean Log2(Intensity) +/- StdErr**

**Variability Gauge Rank=110, protein_id=IPI00297579.3, Annotation=Chromobox_protein_homolog_3**

**Variability Chart for Mean Log2(Intensity) +/- StdErr**

**Variability Gauge Rank=111, protein_id=IPI00304925.3, Annotation=Heat_shock_70_kDa_protein_1**

**Variability Chart for Mean Log2(Intensity) +/- StdErr**

**Variability Gauge Rank=112, protein_id=IPI00028888.1, Annotation=Isoform_1_of_Heterogeneous_nuclear_ribonucleoprotein_D0**

**Variability Chart for Mean Log2(Intensity) +/- StdErr**

**Variability Gauge Rank=113, protein_id=IPI00298547.3, Annotation=Protein_DJ-1**

**Variability Chart for Mean Log2(Intensity) +/- StdErr**

**Variability Gauge Rank=114, protein_id=IPI00218733.5, Annotation=16_kDa_protein**

**Variability Chart for Mean Log2(Intensity) +/- StdErr**

**Variability Gauge Rank=115, protein_id=IPI00179964.5, Annotation=Isoform_1_of_Polypyrimidine_tract-binding_protein_1**

**Variability Chart for Mean Log2(Intensity) +/- StdErr**

**Variability Gauge Rank=116, protein_id=IPI00465248.5, Annotation=enolase_1**

**Variability Chart for Mean Log2(Intensity) +/- StdErr**

**Variability Gauge Rank=117, protein_id=IPI00010740.1, Annotation=Isoform_Long_of_Splicing_factor,_proline-_and_glutamine-rich**

**Variability Chart for Mean Log2(Intensity) +/- StdErr**

**Variability Gauge Rank=118, protein_id=IPI00645078.1, Annotation=Ubiquitin-activating_enzyme_E1**

**Variability Chart for Mean Log2(Intensity) +/- StdErr**

**Variability Gauge Rank=119, protein_id=IPI00186290.5, Annotation=Elongation_factor_2**

**Variability Chart for Mean Log2(Intensity) +/- StdErr**

**Variability Gauge Rank=120, protein_id=IPI00000816.1, Annotation=14-3-3_protein_epsilon**

**Variability Chart for Mean Log2(Intensity) +/- StdErr**

**Variability Gauge Rank=121, protein_id=IPI00219446.4, Annotation=Phosphatidylethanolamine-binding_protein_1**

**Variability Chart for Mean Log2(Intensity) +/- StdErr**

**Variability Gauge Rank=122, protein_id=IPI00465430.5, Annotation=70_kDa_protein**

**Variability Chart for Mean Log2(Intensity) +/- StdErr**

**Variability Gauge Rank=123, protein_id=IPI00014177.3, Annotation=Septin-2**

**Variability Chart for Mean Log2(Intensity) +/- StdErr**

**Variability Gauge Rank=124, protein_id=IPI00293276.9, Annotation=Macrophage_migration_inhibitory_factor**

**Variability Chart for Mean Log2(Intensity) +/- StdErr**

**Variability Gauge Rank=125, protein_id=IPI00000875.5, Annotation=Elongation_factor_1-gamma**

**Variability Chart for Mean Log2(Intensity) +/- StdErr**

**Variability Gauge Rank=126, protein_id=IPI00045109.2, Annotation=Histone_H2A_type_1-A**

**Variability Chart for Mean Log2(Intensity) +/- StdErr**

**Variability Gauge Rank=127, protein_id=5822569, Annotation=A_Chain_A,_Crystal_Structure_Of_Hgstp1-1[v104]_Complexed_With_The_Gsh_Conjugate_Of_(+)-Anti-Bpde**

**Variability Chart for Mean Log2(Intensity) +/- StdErr**

**Variability Gauge Rank=128, protein_id=IPI00220642.6, Annotation=14-3-3_protein_gamma**

**Variability Chart for Mean Log2(Intensity) +/- StdErr**

**Variability Gauge Rank=129, protein_id=IPI00081836.2, Annotation=Histone_H2A_type_1-H**

**Variability Chart for Mean Log2(Intensity) +/- StdErr**

**Variability Gauge Rank=130, protein_id=IPI00027834.3, Annotation=heterogeneous_nuclear_ribonucleoprotein_L_isoform_a**

**Variability Chart for Mean Log2(Intensity) +/- StdErr**

**Variability Gauge Rank=131, protein_id=IPI00419373.1, Annotation=Isoform_1_of_Heterogeneous_nuclear_ribonucleoprotein_A3**

**Variability Chart for Mean Log2(Intensity) +/- StdErr**

**Variability Gauge Rank=132, protein_id=IPI00027444.1, Annotation=Leukocyte_elastase_inhibitor**

**Variability Chart for Mean Log2(Intensity) +/- StdErr**

**Variability Gauge Rank=133, protein_id=IPI00027230.3, Annotation=Endoplasmin_precursor**

**Variability Chart for Mean Log2(Intensity) +/- StdErr**

**Variability Gauge Rank=134, protein_id=IPI00220740.1, Annotation=Isoform_2_of_Nucleophosmin**

**Variability Chart for Mean Log2(Intensity) +/- StdErr**

**Variability Gauge Rank=135, protein_id=IPI00549248.4, Annotation=Isoform_1_of_Nucleophosmin**

**Variability Chart for Mean Log2(Intensity) +/- StdErr**

**Variability Gauge Rank=136, protein_id=18314408, Annotation=Nucleophosmin_(nucleolar_phosphoprotein_B23,_numatrin)_[Homo_sapiens]**

**Variability Chart for Mean Log2(Intensity) +/- StdErr**

**Variability Gauge Rank=137, protein_id=IPI00216457.6, Annotation=Histone_H2A_type_2-A**

**Variability Chart for Mean Log2(Intensity) +/- StdErr**

**Variability Gauge Rank=138, protein_id=IPI00398958.2, Annotation=Ribosomal_protein_SA**

**Variability Chart for Mean Log2(Intensity) +/- StdErr**

**Variability Gauge Rank=139, protein_id=IPI00176692.7, Annotation=PREDICTED:_similar_to_Heterogeneous_nuclear_ribonucleoprotein_A1**

**Variability Chart for Mean Log2(Intensity) +/- StdErr**

**Variability Gauge Rank=140, protein_id=IPI00455423.3, Annotation=PREDICTED:_similar_to_nucleophosmin_1_isoform_1**

**Variability Chart for Mean Log2(Intensity) +/- StdErr**

**Variability Gauge Rank=141, protein_id=IPI00017297.1, Annotation=Matrin-3**

**Variability Chart for Mean Log2(Intensity) +/- StdErr**

**Variability Gauge Rank=142, protein_id=IPI00216730.2, Annotation=Histone_H2A_type_2-B**

**Variability Chart for Mean Log2(Intensity) +/- StdErr**

**Variability Gauge Rank=143, protein_id=IPI00419585.8, Annotation=Peptidyl-prolyl_cis-trans_isomerase_A**

**Variability Chart for Mean Log2(Intensity) +/- StdErr**

**Variability Gauge Rank=144, protein_id=IPI00303476.1, Annotation=ATP_synthase_subunit_beta,_mitochondrial_precursor**

**Variability Chart for Mean Log2(Intensity) +/- StdErr**

**Variability Gauge Rank=145, protein_id=IPI00221089.4, Annotation=40S_ribosomal_protein_S13**

**Variability Chart for Mean Log2(Intensity) +/- StdErr**

**Variability Gauge Rank=146, protein_id=IPI00022774.2, Annotation=Transitional_endoplasmic_reticulum_ATPase**

**Variability Chart for Mean Log2(Intensity) +/- StdErr**

**Variability Gauge Rank=147, protein_id=IPI00555874.1, Annotation=Actin-like_protein_(Fragment)**

**Variability Chart for Mean Log2(Intensity) +/- StdErr**

**Variability Gauge Rank=148, protein_id=IPI00386854.5, Annotation=HNRPA2B1_protein**

**Variability Chart for Mean Log2(Intensity) +/- StdErr**

**Variability Gauge Rank=149, protein_id=IPI00550488.2, Annotation=TALDO1_protein**

**Variability Chart for Mean Log2(Intensity) +/- StdErr**

**Variability Gauge Rank=150, protein_id=IPI00479186.4, Annotation=pyruvate_kinase_3_isoform_1**

**Variability Chart for Mean Log2(Intensity) +/- StdErr**

**Variability Gauge Rank=151, protein_id=IPI00025091.2, Annotation=40S_ribosomal_protein_S11**

**Variability Chart for Mean Log2(Intensity) +/- StdErr**

**Variability Gauge Rank=152, protein_id=IPI00032313.1, Annotation=Protein_S100-A4**

**Variability Chart for Mean Log2(Intensity) +/- StdErr**

**Variability Gauge Rank=153, protein_id=IPI00008603.1, Annotation=Actin,_aortic_smooth_muscle**

**Variability Chart for Mean Log2(Intensity) +/- StdErr**

**Variability Gauge Rank=154, protein_id=IPI00010471.4, Annotation=Plastin-2**

**Variability Chart for Mean Log2(Intensity) +/- StdErr**

**Variability Gauge Rank=155, protein_id=IPI00171611.6, Annotation=Histone_H3.2**

**Variability Chart for Mean Log2(Intensity) +/- StdErr**

**Variability Gauge Rank=156, protein_id=IPI00219038.8, Annotation=Histone_H3.3**

**Variability Chart for Mean Log2(Intensity) +/- StdErr**

**Variability Gauge Rank=157, protein_id=IPI00000760.1, Annotation=NG,NG-dimethylarginine_dimethylaminohydrolase_2**

**Variability Chart for Mean Log2(Intensity) +/- StdErr**

**Variability Gauge Rank=158, protein_id=IPI00179953.2, Annotation=Isoform_1_of_Nuclear_autoantigenic_sperm_protein**

**Variability Chart for Mean Log2(Intensity) +/- StdErr**

**Variability Gauge Rank=159, protein_id=IPI00451401.2, Annotation=Isoform_2_of_Triosephosphate_isomerase**

**Variability Chart for Mean Log2(Intensity) +/- StdErr**

**Variability Gauge Rank=160, protein_id=IPI00021840.1, Annotation=40S_ribosomal_protein_S6**

**Variability Chart for Mean Log2(Intensity) +/- StdErr**

**Variability Gauge Rank=161, protein_id=IPI00396378.3, Annotation=Isoform_B1_of_Heterogeneous_nuclear_ribonucleoproteins_A2/B1**

**Variability Chart for Mean Log2(Intensity) +/- StdErr**

**Variability Gauge Rank=162, protein_id=IPI00003881.5, Annotation=heterogeneous_nuclear_ribonucleoprotein_F**

**Variability Chart for Mean Log2(Intensity) +/- StdErr**

**Variability Gauge Rank=163, protein_id=IPI00412259.2, Annotation=PREDICTED:_similar_to_ATP-dependent_DNA_helicase_2_subunit_1_(ATP-dependent_DNA_helicase_II_70_kDa_subunit)_(Lupus_Ku_autoantigen_protein_p70)_(Ku70)_(70_kDa_subunit_of_Ku_antigen)_(Thyroid-lupus_autoantigen)_(TLAA)_(CTC_box-binding_factor_75_kDa_subunit)_(CT..._isoform_1**

**Variability Chart for Mean Log2(Intensity) +/- StdErr**

**Variability Gauge Rank=164, protein_id=IPI00449049.4, Annotation=Poly_[ADP-ribose]_polymerase_1**

**Variability Chart for Mean Log2(Intensity) +/- StdErr**

**Variability Gauge Rank=165, protein_id=IPI00010204.1, Annotation=Splicing_factor,_arginine/serine-rich_3**

**Variability Chart for Mean Log2(Intensity) +/- StdErr**

**Variability Gauge Rank=166, protein_id=IPI00018146.1, Annotation=14-3-3_protein_theta**

**Variability Chart for Mean Log2(Intensity) +/- StdErr**

**Variability Gauge Rank=167, protein_id=IPI00550239.3, Annotation=Histone_H1.0**

**Variability Chart for Mean Log2(Intensity) +/- StdErr**

**Variability Gauge Rank=168, protein_id=IPI00012011.5, Annotation=Cofilin-1**

**Variability Chart for Mean Log2(Intensity) +/- StdErr**

**Variability Gauge Rank=169, protein_id=IPI00018206.3, Annotation=Aspartate_aminotransferase,_mitochondrial_precursor**

**Variability Chart for Mean Log2(Intensity) +/- StdErr**

**Variability Gauge Rank=170, protein_id=IPI00169383.2, Annotation=Phosphoglycerate_kinase_1**

**Variability Chart for Mean Log2(Intensity) +/- StdErr**

**Variability Gauge Rank=171, protein_id=IPI00031523.3, Annotation=Heat_shock_protein_86_(Fragment)**

**Variability Chart for Mean Log2(Intensity) +/- StdErr**

**Variability Gauge Rank=172, protein_id=31615803, Annotation=A_Chain_A,_Synthetic_Ubiquitin_With_Fluoro-Leu_At_50_And_67**

**Variability Chart for Mean Log2(Intensity) +/- StdErr**

**Variability Gauge Rank=173, protein_id=2627129, Annotation=polyubiquitin_[Homo_sapiens]**

**Variability Chart for Mean Log2(Intensity) +/- StdErr**

**Variability Gauge Rank=174, protein_id=5821952, Annotation=A_Chain_A,_Rotamer_Strain_As_A_Determinant_Of_Protein_Structural_Specificity**

**Variability Chart for Mean Log2(Intensity) +/- StdErr**

**Variability Gauge Rank=175, protein_id=IPI00020956.1, Annotation=Hepatoma-derived_growth_factor**

**Variability Chart for Mean Log2(Intensity) +/- StdErr**

**Variability Gauge Rank=176, protein_id=IPI00027107.5, Annotation=Tu_translation_elongation_factor,_mitochondrial**

**Variability Chart for Mean Log2(Intensity) +/- StdErr**

**Variability Gauge Rank=177, protein_id=IPI00550021.3, Annotation=60S_ribosomal_protein_L3**

**Variability Chart for Mean Log2(Intensity) +/- StdErr**

**Variability Gauge Rank=178, protein_id=IPI00479997.3, Annotation=Stathmin**

**Variability Chart for Mean Log2(Intensity) +/- StdErr**

**Variability Gauge Rank=179, protein_id=IPI00744692.1, Annotation=Transaldolase**

**Variability Chart for Mean Log2(Intensity) +/- StdErr**

**Variability Gauge Rank=180, protein_id=IPI00171903.1, Annotation=heterogeneous_nuclear_ribonucleoprotein_M_isoform_a**

**Variability Chart for Mean Log2(Intensity) +/- StdErr**

**Variability Gauge Rank=181, protein_id=IPI00554676.1, Annotation=Hemoglobin_subunit_gamma-2**

**Variability Chart for Mean Log2(Intensity) +/- StdErr**

**Variability Gauge Rank=182, protein_id=IPI00013415.1, Annotation=40S_ribosomal_protein_S7**

**Variability Chart for Mean Log2(Intensity) +/- StdErr**

**Variability Gauge Rank=183, protein_id=IPI00216171.2, Annotation=Gamma-enolase**

**Variability Chart for Mean Log2(Intensity) +/- StdErr**

**Variability Gauge Rank=184, protein_id=IPI00383071.1, Annotation=RcTPI1_(Fragment)**

**Variability Chart for Mean Log2(Intensity) +/- StdErr**

**Variability Gauge Rank=185, protein_id=229149, Annotation=610523A_hemoglobin_beta**

**Variability Chart for Mean Log2(Intensity) +/- StdErr**

**Variability Gauge Rank=186, protein_id=IPI00219217.2, Annotation=L-lactate_dehydrogenase_B_chain**

**Variability Chart for Mean Log2(Intensity) +/- StdErr**

**Variability Gauge Rank=187, protein_id=IPI00003362.2, Annotation=Hypothetical_protein**

**Variability Chart for Mean Log2(Intensity) +/- StdErr**

**Variability Gauge Rank=188, protein_id=180663, Annotation=c-myc_binding_protein_[Homo_sapiens]**

**Variability Chart for Mean Log2(Intensity) +/- StdErr**

**Variability Gauge Rank=189, protein_id=IPI00219568.3, Annotation=Phosphoglycerate_kinase,_testis_specific**

**Variability Chart for Mean Log2(Intensity) +/- StdErr**

**Variability Gauge Rank=190, protein_id=IPI00012074.3, Annotation=Heterogeneous_nuclear_ribonucleoprotein_R**

**Variability Chart for Mean Log2(Intensity) +/- StdErr**

**Variability Gauge Rank=191, protein_id=IPI00183526.5, Annotation=NCL_protein**

**Variability Chart for Mean Log2(Intensity) +/- StdErr**

**Variability Gauge Rank=192, protein_id=IPI00026272.1, Annotation=Histone_H2A_type_1-B**

**Variability Chart for Mean Log2(Intensity) +/- StdErr**

**Variability Gauge Rank=193, protein_id=IPI00005978.7, Annotation=Splicing_factor,_arginine/serine-rich_2**

**Variability Chart for Mean Log2(Intensity) +/- StdErr**

**Variability Gauge Rank=194, protein_id=IPI00010402.2, Annotation=Hypothetical_protein**

**Variability Chart for Mean Log2(Intensity) +/- StdErr**

**Variability Gauge Rank=195, protein_id=IPI00386491.5, Annotation=Isoform_Short_of_Heterogeneous_nuclear_ribonucleoprotein_U**

**Variability Chart for Mean Log2(Intensity) +/- StdErr**

**Variability Gauge Rank=196, protein_id=IPI00291006.1, Annotation=Malate_dehydrogenase,_mitochondrial_precursor**

**Variability Chart for Mean Log2(Intensity) +/- StdErr**

**Variability Gauge Rank=197, protein_id=IPI00654755.2, Annotation=Hemoglobin_subunit_beta**

**Variability Chart for Mean Log2(Intensity) +/- StdErr**

**Variability Gauge Rank=198, protein_id=IPI00220644.7, Annotation=Isoform_M1_of_Pyruvate_kinase_isozymes_M1/M2**

**Variability Chart for Mean Log2(Intensity) +/- StdErr**

**Variability Gauge Rank=199, protein_id=IPI00411706.1, Annotation=S-formylglutathione_hydrolase**

**Variability Chart for Mean Log2(Intensity) +/- StdErr**

**Variability Gauge Rank=200, protein_id=IPI00215901.1, Annotation=adenylate_kinase_2_isoform_a**

**Variability Chart for Mean Log2(Intensity) +/- StdErr**

**Variability Gauge Rank=201, protein_id=178067, Annotation=actin_prepeptide**

**Variability Chart for Mean Log2(Intensity) +/- StdErr**

**Variability Gauge Rank=202, protein_id=IPI00018349.5, Annotation=DNA_replication_licensing_factor_MCM4**

**Variability Chart for Mean Log2(Intensity) +/- StdErr**

**Variability Gauge Rank=203, protein_id=IPI00075248.10, Annotation=Calmodulin**

**Variability Chart for Mean Log2(Intensity) +/- StdErr**

**Variability Gauge Rank=204, protein_id=IPI00018278.2, Annotation=Histone_H2AV**

**Variability Chart for Mean Log2(Intensity) +/- StdErr**

**Variability Gauge Rank=205, protein_id=IPI00220706.9, Annotation=Hemoglobin_subunit_gamma-1**

**Variability Chart for Mean Log2(Intensity) +/- StdErr**

**Variability Gauge Rank=206, protein_id=IPI00014581.1, Annotation=Isoform_1_of_Tropomyosin_1_alpha_chain**

**Variability Chart for Mean Log2(Intensity) +/- StdErr**

**Variability Gauge Rank=207, protein_id=IPI00219806.5, Annotation=Protein_S100-A7**

**Variability Chart for Mean Log2(Intensity) +/- StdErr**

**Variability Gauge Rank=208, protein_id=IPI00218474.4, Annotation=Beta-enolase**

**Variability Chart for Mean Log2(Intensity) +/- StdErr**

**Variability Gauge Rank=209, protein_id=IPI00021428.1, Annotation=Actin,_alpha_skeletal_muscle**

**Variability Chart for Mean Log2(Intensity) +/- StdErr**

**Variability Gauge Rank=210, protein_id=IPI00215965.1, Annotation=heterogeneous_nuclear_ribonucleoprotein_A1_isoform_b**

**Variability Chart for Mean Log2(Intensity) +/- StdErr**

**Variability Gauge Rank=211, protein_id=IPI00007797.2, Annotation=Fatty_acid-binding_protein,_epidermal**

**Variability Chart for Mean Log2(Intensity) +/- StdErr**

**Variability Gauge Rank=212, protein_id=IPI00216691.4, Annotation=Profilin-1**

**Variability Chart for Mean Log2(Intensity) +/- StdErr**

**Variability Gauge Rank=213, protein_id=IPI00018755.1, Annotation=High_mobility_group_protein_1-like_10**

**Variability Chart for Mean Log2(Intensity) +/- StdErr**

**Variability Gauge Rank=214, protein_id=IPI00003269.1, Annotation=hypothetical_protein_LOC345651**

**Variability Chart for Mean Log2(Intensity) +/- StdErr**

**Variability Gauge Rank=215, protein_id=IPI00383556.1, Annotation=Heterogeneous_nuclear_ribonucleoprotein_UP2_(Fragment)**

**Variability Chart for Mean Log2(Intensity) +/- StdErr**

**Variability Gauge Rank=216, protein_id=IPI00384051.4, Annotation=Proteasome_activator_complex_subunit_2**

**Variability Chart for Mean Log2(Intensity) +/- StdErr**

**Variability Gauge Rank=217, protein_id=IPI00003815.2, Annotation=Rho_GDP-dissociation_inhibitor_1**

**Variability Chart for Mean Log2(Intensity) +/- StdErr**

**Variability Gauge Rank=218, protein_id=IPI00419258.3, Annotation=High_mobility_group_protein_B1**

**Variability Chart for Mean Log2(Intensity) +/- StdErr**

**Variability Gauge Rank=219, protein_id=IPI00215884.3, Annotation=splicing_factor,_arginine/serine-rich_1**

**Variability Chart for Mean Log2(Intensity) +/- StdErr**

**Variability Gauge Rank=220, protein_id=IPI00465070.6, Annotation=Histone_H3.1**

**Variability Chart for Mean Log2(Intensity) +/- StdErr**

**Variability Gauge Rank=221, protein_id=IPI00018140.3, Annotation=Isoform_1_of_Heterogeneous_nuclear_ribonucleoprotein_Q**

**Variability Chart for Mean Log2(Intensity) +/- StdErr**

**Variability Gauge Rank=222, protein_id=IPI00010133.2, Annotation=Coronin-1A**

**Variability Chart for Mean Log2(Intensity) +/- StdErr**

**Variability Gauge Rank=223, protein_id=IPI00221093.6, Annotation=40S_ribosomal_protein_S17**

**Variability Chart for Mean Log2(Intensity) +/- StdErr**

**Variability Gauge Rank=224, protein_id=IPI00217975.3, Annotation=Lamin-B1**

**Variability Chart for Mean Log2(Intensity) +/- StdErr**

**Variability Gauge Rank=225, protein_id=IPI00072377.1, Annotation=Isoform_1_of_Protein_SET**

**Variability Chart for Mean Log2(Intensity) +/- StdErr**

**Variability Gauge Rank=226, protein_id=223582, Annotation=0901261A_histone_H4**

**Variability Chart for Mean Log2(Intensity) +/- StdErr**

**Variability Gauge Rank=227, protein_id=IPI00027569.1, Annotation=Heterogeneous_nuclear_ribonucleoprotein_C-like_1**

**Variability Chart for Mean Log2(Intensity) +/- StdErr**

**Variability Gauge Rank=228, protein_id=IPI00453473.5, Annotation=Histone_H4**

**Variability Chart for Mean Log2(Intensity) +/- StdErr**

**Variability Gauge Rank=229, protein_id=IPI00163782.2, Annotation=Isoform_2_of_Far_upstream_element-binding_protein_1**

**Variability Chart for Mean Log2(Intensity) +/- StdErr**

**Variability Gauge Rank=230, protein_id=IPI00304596.3, Annotation=Non-POU_domain-containing_octamer-binding_protein**

**Variability Chart for Mean Log2(Intensity) +/- StdErr**

**Variability Gauge Rank=231, protein_id=IPI00298994.5, Annotation=271_kDa_protein**

**Variability Chart for Mean Log2(Intensity) +/- StdErr**

**Variability Gauge Rank=232, protein_id=IPI00402104.5, Annotation=15_kDa_protein**

**Variability Chart for Mean Log2(Intensity) +/- StdErr**

**Variability Gauge Rank=233, protein_id=IPI00217966.6, Annotation=lactate_dehydrogenase_A**

**Variability Chart for Mean Log2(Intensity) +/- StdErr**

**Variability Gauge Rank=234, protein_id=37654352, Annotation=H3L-like_histone_[Homo_sapiens]**

**Variability Chart for Mean Log2(Intensity) +/- StdErr**

**Variability Gauge Rank=235, protein_id=IPI00000874.1, Annotation=Peroxiredoxin-1**

**Variability Chart for Mean Log2(Intensity) +/- StdErr**

**Variability Gauge Rank=236, protein_id=IPI00219097.3, Annotation=High_mobility_group_protein_B2**

**Variability Chart for Mean Log2(Intensity) +/- StdErr**

**Variability Gauge Rank=237, protein_id=IPI00172460.3, Annotation=Isoform_3_of_Adenylate_kinase_isoenzyme_2,_mitochondrial**

**Variability Chart for Mean Log2(Intensity) +/- StdErr**

**Variability Gauge Rank=238, protein_id=IPI00444262.1, Annotation=CDNA_FLJ45706_fis,_clone_FEBRA2028457,_highly_similar_to_Nucleolin**

**Variability Chart for Mean Log2(Intensity) +/- StdErr**

**Variability Gauge Rank=239, protein_id=IPI00335132.2, Annotation=22_kDa_protein**

**Variability Chart for Mean Log2(Intensity) +/- StdErr**

**Variability Gauge Rank=240, protein_id=IPI00221354.1, Annotation=Isoform_Short_of_RNA-binding_protein_FUS**

**Variability Chart for Mean Log2(Intensity) +/- StdErr**

**Variability Gauge Rank=241, protein_id=IPI00013881.5, Annotation=heterogeneous_nuclear_ribonucleoprotein_H1**

**Variability Chart for Mean Log2(Intensity) +/- StdErr**

**Variability Gauge Rank=242, protein_id=IPI00017367.5, Annotation=Radixin**

**Variability Chart for Mean Log2(Intensity) +/- StdErr**

**Variability Gauge Rank=243, protein_id=IPI00384282.1, Annotation=Cytovillin_2_(Fragment)**

**Variability Chart for Mean Log2(Intensity) +/- StdErr**

**Variability Gauge Rank=244, protein_id=IPI00030154.1, Annotation=Proteasome_activator_complex_subunit_1**

**Variability Chart for Mean Log2(Intensity) +/- StdErr**

**Variability Gauge Rank=245, protein_id=IPI00299571.4, Annotation=CDNA_FLJ45525_fis,_clone_BRTHA2026311,_highly_similar_to_Protein_disulfide_isomerase_A6**

**Variability Chart for Mean Log2(Intensity) +/- StdErr**

**Variability Gauge Rank=246, protein_id=IPI00219365.2, Annotation=Moesin**

**Variability Chart for Mean Log2(Intensity) +/- StdErr**

**Variability Gauge Rank=247, protein_id=IPI00023785.5, Annotation=Isoform_1_of_Probable_ATP-dependent_RNA_helicase_DDX17**

**Variability Chart for Mean Log2(Intensity) +/- StdErr**

**Variability Gauge Rank=248, protein_id=IPI00215780.4, Annotation=40S_ribosomal_protein_S19**

**Variability Chart for Mean Log2(Intensity) +/- StdErr**

**Variability Gauge Rank=249, protein_id=IPI00787316.1, Annotation=PREDICTED:_similar_to_peptidylprolyl_isomerase_A_isoform_1**

**Variability Chart for Mean Log2(Intensity) +/- StdErr**

**Variability Gauge Rank=250, protein_id=230867, Annotation=R_Chain_R,_Twinning_In_Crystals_Of_Human_Skeletal_Muscle_D-_Glyceraldehyde-3-Phosphate_Dehydrogenase**

**Variability Chart for Mean Log2(Intensity) +/- StdErr**

**Variability Gauge Rank=251, protein_id=37403, Annotation=unnamed_protein_product_[Homo_sapiens]**

**Variability Chart for Mean Log2(Intensity) +/- StdErr**

**Variability Gauge Rank=252, protein_id=IPI00377005.1, Annotation=RcTPM3_(Fragment)**

**Variability Chart for Mean Log2(Intensity) +/- StdErr**

**Variability Gauge Rank=253, protein_id=IPI00025252.1, Annotation=Protein_disulfide-isomerase_A3_precursor**

**Variability Chart for Mean Log2(Intensity) +/- StdErr**

**Variability Gauge Rank=254, protein_id=IPI00298860.5, Annotation=Growth-inhibiting_protein_12**

**Variability Chart for Mean Log2(Intensity) +/- StdErr**

**Variability Gauge Rank=255, protein_id=IPI00395772.4, Annotation=Hypothetical_protein_DKFZp451J0218**

**Variability Chart for Mean Log2(Intensity) +/- StdErr**

**Variability Gauge Rank=256, protein_id=IPI00024933.3, Annotation=60S_ribosomal_protein_L12**

**Variability Chart for Mean Log2(Intensity) +/- StdErr**

**Variability Gauge Rank=257, protein_id=IPI00021263.3, Annotation=14-3-3_protein_zeta/delta**

**Variability Chart for Mean Log2(Intensity) +/- StdErr**

**Variability Gauge Rank=258, protein_id=IPI00022796.1, Annotation=OTTHUMP00000031372**

**Variability Chart for Mean Log2(Intensity) +/- StdErr**

**Variability Gauge Rank=259, protein_id=IPI00027462.1, Annotation=Protein_S100-A9**

**Variability Chart for Mean Log2(Intensity) +/- StdErr**

**Variability Gauge Rank=260, protein_id=1585552, Annotation=2201353A_glucose-regulated_protein_ERp57/GRP58**

**Variability Chart for Mean Log2(Intensity) +/- StdErr**

**Variability Gauge Rank=261, protein_id=IPI00013877.2, Annotation=Isoform_1_of_Heterogeneous_nuclear_ribonucleoprotein_H3**

**Variability Chart for Mean Log2(Intensity) +/- StdErr**

**Variability Gauge Rank=262, protein_id=IPI00000494.5, Annotation=60S_ribosomal_protein_L5**

**Variability Chart for Mean Log2(Intensity) +/- StdErr**

**Variability Gauge Rank=263, protein_id=IPI00003377.1, Annotation=Isoform_1_of_Splicing_factor,_arginine/serine-rich_7**

**Variability Chart for Mean Log2(Intensity) +/- StdErr**

**Variability Gauge Rank=264, protein_id=IPI00027626.2, Annotation=T-complex_protein_1_subunit_zeta**

**Variability Chart for Mean Log2(Intensity) +/- StdErr**

**Variability Gauge Rank=265, protein_id=IPI00011200.4, Annotation=D-3-phosphoglycerate_dehydrogenase**

**Variability Chart for Mean Log2(Intensity) +/- StdErr**

**Variability Gauge Rank=266, protein_id=IPI00178083.2, Annotation=29_kDa_protein**

**Variability Chart for Mean Log2(Intensity) +/- StdErr**

**Variability Gauge Rank=267, protein_id=IPI00005613.2, Annotation=Splicing_factor_U2AF_35_kDa_subunit**

**Variability Chart for Mean Log2(Intensity) +/- StdErr**

**Variability Gauge Rank=268, protein_id=IPI00024114.3, Annotation=Putative_GTP-binding_protein_5**

**Variability Chart for Mean Log2(Intensity) +/- StdErr**

**Variability Gauge Rank=269, protein_id=IPI00219798.1, Annotation=Isoform_1_of_Roundabout_homolog_1_precursor**

**Variability Chart for Mean Log2(Intensity) +/- StdErr**

**Variability Gauge Rank=270, protein_id=IPI00013894.1, Annotation=Stress-induced-phosphoprotein_1**

**Variability Chart for Mean Log2(Intensity) +/- StdErr**

**Variability Gauge Rank=271, protein_id=IPI00000230.5, Annotation=tropomyosin_1_alpha_chain_isoform_2**

**Variability Chart for Mean Log2(Intensity) +/- StdErr**

**Variability Gauge Rank=272, protein_id=IPI00328301.4, Annotation=CDNA_FLJ46103_fis,_clone_TESTI2023903,_weakly_similar_to_Homo_sapiens_ubiquilin_1**

**Variability Chart for Mean Log2(Intensity) +/- StdErr**

**Variability Gauge Rank=273, protein_id=IPI00027252.6, Annotation=Prohibitin-2**

**Variability Chart for Mean Log2(Intensity) +/- StdErr**

**Variability Gauge Rank=274, protein_id=IPI00027434.1, Annotation=Rho-related_GTP-binding_protein_RhoC_precursor**

**Variability Chart for Mean Log2(Intensity) +/- StdErr**

**Variability Gauge Rank=275, protein_id=IPI00030985.1, Annotation=Protein_FAM96A**

**Variability Chart for Mean Log2(Intensity) +/- StdErr**

**Variability Gauge Rank=276, protein_id=IPI00219525.9, Annotation=6-phosphogluconate_dehydrogenase,_decarboxylating**

**Variability Chart for Mean Log2(Intensity) +/- StdErr**

**Variability Gauge Rank=277, protein_id=IPI00739464.1, Annotation=PREDICTED:_similar_to_cytoplasmic_beta-actin**

**Variability Chart for Mean Log2(Intensity) +/- StdErr**

**Variability Gauge Rank=278, protein_id=3411134, Annotation=mutant_desmin_[Homo_sapiens]**

**Variability Chart for Mean Log2(Intensity) +/- StdErr**

**Variability Gauge Rank=279, protein_id=IPI00304692.1, Annotation=Heterogeneous_nuclear_ribonucleoprotein_G**

**Variability Chart for Mean Log2(Intensity) +/- StdErr**

**Variability Gauge Rank=280, protein_id=IPI00034319.2, Annotation=Isoform_A_of_Protein_CutA_precursor**

**Variability Chart for Mean Log2(Intensity) +/- StdErr**

**Variability Gauge Rank=281, protein_id=IPI00001453.2, Annotation=Alpha-internexin**

**Variability Chart for Mean Log2(Intensity) +/- StdErr**

**Variability Gauge Rank=282, protein_id=IPI00743010.1, Annotation=38_kDa_protein**

**Variability Chart for Mean Log2(Intensity) +/- StdErr**

**Variability Gauge Rank=283, protein_id=IPI00743972.1, Annotation=Conserved_hypothetical_protein**

**Variability Chart for Mean Log2(Intensity) +/- StdErr**

**Variability Gauge Rank=284, protein_id=IPI00787038.1, Annotation=PREDICTED:_family_with_sequence_similarity_92,_member_A3**

**Variability Chart for Mean Log2(Intensity) +/- StdErr**

**Variability Gauge Rank=285, protein_id=IPI00305978.4, Annotation=Aflatoxin_B1_aldehyde_reductase_member_2**

**Variability Chart for Mean Log2(Intensity) +/- StdErr**

**Variability Gauge Rank=286, protein_id=IPI00027410.1, Annotation=Platelet_glycoprotein_V_precursor**

**Variability Chart for Mean Log2(Intensity) +/- StdErr**

**Variability Gauge Rank=287, protein_id=IPI00292470.2, Annotation=OTTHUMP00000017658**

**Variability Chart for Mean Log2(Intensity) +/- StdErr**

**Variability Gauge Rank=288, protein_id=IPI00479962.3, Annotation=Myosin-5B**

**Variability Chart for Mean Log2(Intensity) +/- StdErr**

**Variability Gauge Rank=289, protein_id=IPI00000606.4, Annotation=Tetratricopeptide_repeat_protein_4**

**Variability Chart for Mean Log2(Intensity) +/- StdErr**

**Variability Gauge Rank=290, protein_id=IPI00005721.1, Annotation=Neutrophil_defensin_1_precursor**

**Variability Chart for Mean Log2(Intensity) +/- StdErr**

**Variability Gauge Rank=291, protein_id=IPI00008575.3, Annotation=Isoform_1_of_KH_domain-containing,_RNA-binding,_signal_transduction-associated_protein_1**

**Variability Chart for Mean Log2(Intensity) +/- StdErr**

**Variability Gauge Rank=292, protein_id=IPI00024046.1, Annotation=Cadherin-13_precursor**

**Variability Chart for Mean Log2(Intensity) +/- StdErr**

**Variability Gauge Rank=293, protein_id=IPI00215790.5, Annotation=60S_ribosomal_protein_L38**

**Variability Chart for Mean Log2(Intensity) +/- StdErr**

**Variability Gauge Rank=294, protein_id=IPI00291005.7, Annotation=Malate_dehydrogenase,_cytoplasmic**

**Variability Chart for Mean Log2(Intensity) +/- StdErr**

**Variability Gauge Rank=295, protein_id=IPI00007858.1, Annotation=Myosin-13**

**Variability Chart for Mean Log2(Intensity) +/- StdErr**

**Variability Gauge Rank=296, protein_id=IPI00014230.1, Annotation=Complement_component_1_Q_subcomponent-binding_protein,_mitochondrial_precursor**

**Variability Chart for Mean Log2(Intensity) +/- StdErr**

**Variability Gauge Rank=297, protein_id=IPI00014263.1, Annotation=eukaryotic_translation_initiation_factor_4H_isoform_1**

**Variability Chart for Mean Log2(Intensity) +/- StdErr**

**Variability Gauge Rank=298, protein_id=IPI00005715.5, Annotation=Isoform_1_of_Ubiquitin_conjugation_factor_E4_B**

**Variability Chart for Mean Log2(Intensity) +/- StdErr**

**Variability Gauge Rank=299, protein_id=IPI00374260.4, Annotation=PREDICTED:_similar_to_ribosomal_protein_L10_isoform_1**

**Variability Chart for Mean Log2(Intensity) +/- StdErr**

**Variability Gauge Rank=300, protein_id=IPI00012320.1, Annotation=High_affinity_interleukin-8_receptor_B**

**Variability Chart for Mean Log2(Intensity) +/- StdErr**

**Variability Gauge Rank=301, protein_id=IPI00011229.1, Annotation=Cathepsin_D_precursor**

**Variability Chart for Mean Log2(Intensity) +/- StdErr**

**Variability Gauge Rank=302, protein_id=IPI00006935.2, Annotation=Eukaryotic_translation_initiation_factor_5A-2**

**Variability Chart for Mean Log2(Intensity) +/- StdErr**

**Variability Gauge Rank=303, protein_id=IPI00302850.4, Annotation=Small_nuclear_ribonucleoprotein_Sm_D1**

**Variability Chart for Mean Log2(Intensity) +/- StdErr**

**Variability Gauge Rank=304, protein_id=IPI00397611.1, Annotation=PREDICTED:_similar_to_ribosomal_protein_L13_isoform_1**

**Variability Chart for Mean Log2(Intensity) +/- StdErr**

**Variability Gauge Rank=305, protein_id=7020000, Annotation=unnamed_protein_product_[Homo_sapiens]**

**Variability Chart for Mean Log2(Intensity) +/- StdErr**

**Variability Gauge Rank=306, protein_id=IPI00025512.2, Annotation=Heat-shock_protein_beta-1**

**Variability Chart for Mean Log2(Intensity) +/- StdErr**

**Variability Gauge Rank=307, protein_id=IPI00003438.2, Annotation=DnaJ_homolog_subfamily_C_member_8**

**Variability Chart for Mean Log2(Intensity) +/- StdErr**

**Variability Gauge Rank=308, protein_id=IPI00013164.3, Annotation=Peripherin**

**Variability Chart for Mean Log2(Intensity) +/- StdErr**

**Variability Gauge Rank=309, protein_id=7661920, Annotation=DEAD_(Asp-Glu-Ala-Asp)_box_polypeptide_48_[Homo_sapiens]**

**Variability Chart for Mean Log2(Intensity) +/- StdErr**

**Variability Gauge Rank=310, protein_id=IPI00009328.3, Annotation=Probable_ATP-dependent_RNA_helicase_DDX48**

**Variability Chart for Mean Log2(Intensity) +/- StdErr**

**Variability Gauge Rank=311, protein_id=IPI00021766.4, Annotation=Isoform_1_of_Reticulon-4**

**Variability Chart for Mean Log2(Intensity) +/- StdErr**

**Variability Gauge Rank=312, protein_id=IPI00002135.1, Annotation=Transforming_acidic_coiled-coil-containing_protein_3**

**Variability Chart for Mean Log2(Intensity) +/- StdErr**

**Variability Gauge Rank=313, protein_id=IPI00013679.1, Annotation=Isoform_DUT-M_of_Deoxyuridine_5'-triphosphate_nucleotidohydrolase,_mitochondrial_precursor**

**Variability Chart for Mean Log2(Intensity) +/- StdErr**

**Variability Gauge Rank=314, protein_id=IPI00026087.1, Annotation=Barrier-to-autointegration_factor**

**Variability Chart for Mean Log2(Intensity) +/- StdErr**

**Variability Gauge Rank=315, protein_id=IPI00012966.1, Annotation=Transcription_factor_12**

**Variability Chart for Mean Log2(Intensity) +/- StdErr**

**Variability Gauge Rank=316, protein_id=IPI00183110.7, Annotation=Isoform_1_of_Paternally_expressed_gene_3_protein**

**Variability Chart for Mean Log2(Intensity) +/- StdErr**

**Variability Gauge Rank=317, protein_id=IPI00012493.1, Annotation=40S_ribosomal_protein_S20**

**Variability Chart for Mean Log2(Intensity) +/- StdErr**

**Variability Gauge Rank=318, protein_id=IPI00742127.2, Annotation=Pseudogene_candidate**

**Variability Chart for Mean Log2(Intensity) +/- StdErr**

**Variability Gauge Rank=319, protein_id=IPI00010896.2, Annotation=Chloride_intracellular_channel_protein_1**

**Variability Chart for Mean Log2(Intensity) +/- StdErr**

**Variability Gauge Rank=320, protein_id=IPI00071059.3, Annotation=BCL2-associated_X_protein_isoform_epsilon**

**Variability Chart for Mean Log2(Intensity) +/- StdErr**

**Variability Gauge Rank=321, protein_id=IPI00744669.2, Annotation=hypothetical_protein_LOC153643**

**Variability Chart for Mean Log2(Intensity) +/- StdErr**

**Variability Gauge Rank=322, protein_id=IPI00030275.5, Annotation=Heat_shock_protein_75_kDa,_mitochondrial_precursor**

**Variability Chart for Mean Log2(Intensity) +/- StdErr**

**Variability Gauge Rank=323, protein_id=IPI00103655.1, Annotation=Isoform_Long_of_Autism_susceptibility_gene_2_protein**

**Variability Chart for Mean Log2(Intensity) +/- StdErr**

**Variability Gauge Rank=324, protein_id=IPI00029631.1, Annotation=Enhancer_of_rudimentary_homolog**

**Variability Chart for Mean Log2(Intensity) +/- StdErr**

**Variability Gauge Rank=325, protein_id=IPI00217236.3, Annotation=Tubulin-specific_chaperone_A**

**Variability Chart for Mean Log2(Intensity) +/- StdErr**

**Variability Gauge Rank=326, protein_id=IPI00022445.1, Annotation=Platelet_basic_protein_precursor**

**Variability Chart for Mean Log2(Intensity) +/- StdErr**

**Variability Gauge Rank=327, protein_id=IPI00157890.3, Annotation=10_kDa_protein**

**Variability Chart for Mean Log2(Intensity) +/- StdErr**

**Variability Gauge Rank=328, protein_id=IPI00167706.4, Annotation=CDNA_FLJ37464_fis,_clone_BRAWH2011795,_weakly_similar_to_LIVER_CARBOXYLESTERASE**

**Variability Chart for Mean Log2(Intensity) +/- StdErr**

**Variability Gauge Rank=329, protein_id=IPI00411985.2, Annotation=Carboxylesterase_Hlo**

**Variability Chart for Mean Log2(Intensity) +/- StdErr**

**Variability Gauge Rank=330, protein_id=IPI00023048.3, Annotation=Elongation_factor_1-delta**

**Variability Chart for Mean Log2(Intensity) +/- StdErr**

**Variability Gauge Rank=331, protein_id=IPI00011400.1, Annotation=T-lymphoma_invasion_and_metastasis-inducing_protein_1**

**Variability Chart for Mean Log2(Intensity) +/- StdErr**

**Variability Gauge Rank=332, protein_id=IPI00017855.1, Annotation=Aconitate_hydratase,_mitochondrial_precursor**

**Variability Chart for Mean Log2(Intensity) +/- StdErr**

**Variability Gauge Rank=333, protein_id=IPI00216318.5, Annotation=tyrosine_3-monooxygenase/tryptophan_5-monooxygenase_activation_protein,_beta_polypeptide**

**Variability Chart for Mean Log2(Intensity) +/- StdErr**

**Variability Gauge Rank=334, protein_id=IPI00332656.3, Annotation=29_kDa_protein**

**Variability Chart for Mean Log2(Intensity) +/- StdErr**

**Variability Gauge Rank=335, protein_id=IPI00183913.2, Annotation=Bone_specific_CMF608**

**Variability Chart for Mean Log2(Intensity) +/- StdErr**

**Variability Gauge Rank=336, protein_id=IPI00006024.4, Annotation=Isoform_2_of_Dedicator_of_cytokinesis_protein_4**

**Variability Chart for Mean Log2(Intensity) +/- StdErr**

**Variability Gauge Rank=337, protein_id=IPI00029744.1, Annotation=Single-stranded_DNA-binding_protein,_mitochondrial_precursor**

**Variability Chart for Mean Log2(Intensity) +/- StdErr**

**Variability Gauge Rank=338, protein_id=IPI00022314.1, Annotation=Superoxide_dismutase_[Mn],_mitochondrial_precursor**

**Variability Chart for Mean Log2(Intensity) +/- StdErr**

**Variability Gauge Rank=339, protein_id=IPI00026119.6, Annotation=Ubiquitin-activating_enzyme_E1**

**Variability Chart for Mean Log2(Intensity) +/- StdErr**

**Variability Gauge Rank=340, protein_id=IPI00291510.3, Annotation=Inosine-5'-monophosphate_dehydrogenase_2**

**Variability Chart for Mean Log2(Intensity) +/- StdErr**

**Variability Gauge Rank=341, protein_id=IPI00021107.4, Annotation=xylulokinase_homolog**

**Variability Chart for Mean Log2(Intensity) +/- StdErr**

**Variability Gauge Rank=342, protein_id=28590, Annotation=unnamed_protein_product_[Homo_sapiens]**

**Variability Chart for Mean Log2(Intensity) +/- StdErr**

**Variability Gauge Rank=343, protein_id=IPI00140827.3, Annotation=PREDICTED:_similar_to_SMT3_suppressor_of_mif_two_3_homolog_2**

**Variability Chart for Mean Log2(Intensity) +/- StdErr**

**Variability Gauge Rank=344, protein_id=IPI00027202.2, Annotation=Zinc_finger_Y-chromosomal_protein**

**Variability Chart for Mean Log2(Intensity) +/- StdErr**

**Variability Gauge Rank=345, protein_id=IPI00179589.2, Annotation=Myotrophin**

**Variability Chart for Mean Log2(Intensity) +/- StdErr**

**Variability Gauge Rank=346, protein_id=IPI00024662.1, Annotation=Chromobox_protein_homolog_5**

**Variability Chart for Mean Log2(Intensity) +/- StdErr**

**Variability Gauge Rank=347, protein_id=IPI00013214.1, Annotation=DNA_replication_licensing_factor_MCM3**

**Variability Chart for Mean Log2(Intensity) +/- StdErr**

**Variability Gauge Rank=348, protein_id=IPI00220362.4, Annotation=10_kDa_heat_shock_protein,_mitochondrial**

**Variability Chart for Mean Log2(Intensity) +/- StdErr**

**Variability Gauge Rank=349, protein_id=IPI00218407.5, Annotation=Fructose-bisphosphate_aldolase_B**

**Variability Chart for Mean Log2(Intensity) +/- StdErr**

**Variability Gauge Rank=350, protein_id=IPI00021700.3, Annotation=Proliferating_cell_nuclear_antigen**

**Variability Chart for Mean Log2(Intensity) +/- StdErr**

**Variability Gauge Rank=351, protein_id=IPI00294627.2, Annotation=Isoform_2_of_Splicing_factor_1**

**Variability Chart for Mean Log2(Intensity) +/- StdErr**

**Variability Gauge Rank=352, protein_id=IPI00007176.4, Annotation=Isoform_2_of_Roundabout_homolog_3_precursor**

**Variability Chart for Mean Log2(Intensity) +/- StdErr**

**Variability Gauge Rank=353, protein_id=IPI00010154.3, Annotation=Rab_GDP_dissociation_inhibitor_alpha**

**Variability Chart for Mean Log2(Intensity) +/- StdErr**

**Variability Gauge Rank=354, protein_id=IPI00031461.1, Annotation=Rab_GDP_dissociation_inhibitor_beta**

**Variability Chart for Mean Log2(Intensity) +/- StdErr**

**Variability Gauge Rank=355, protein_id=IPI00010796.1, Annotation=Protein_disulfide-isomerase_precursor**

**Variability Chart for Mean Log2(Intensity) +/- StdErr**

**Variability Gauge Rank=356, protein_id=IPI00140250.2, Annotation=Probable_G-protein_coupled_receptor_61**

**Variability Chart for Mean Log2(Intensity) +/- StdErr**

**Variability Gauge Rank=357, protein_id=IPI00305383.1, Annotation=Ubiquinol-cytochrome-c_reductase_complex_core_protein_2,_mitochondrial_precursor**

**Variability Chart for Mean Log2(Intensity) +/- StdErr**

**Variability Gauge Rank=358, protein_id=IPI00218570.5, Annotation=Phosphoglycerate_mutase_2**

**Variability Chart for Mean Log2(Intensity) +/- StdErr**

**Variability Gauge Rank=359, protein_id=IPI00180240.2, Annotation=thymosin-like_3**

**Variability Chart for Mean Log2(Intensity) +/- StdErr**

**Variability Gauge Rank=360, protein_id=IPI00106509.2, Annotation=Isoform_4_of_Heterogeneous_nuclear_ribonucleoprotein_A/B**

**Variability Chart for Mean Log2(Intensity) +/- StdErr**

**Variability Gauge Rank=361, protein_id=IPI00376798.3, Annotation=ribosomal_protein_L11**

**Variability Chart for Mean Log2(Intensity) +/- StdErr**

**Variability Gauge Rank=362, protein_id=IPI00010720.1, Annotation=T-complex_protein_1_subunit_epsilon**

**Variability Chart for Mean Log2(Intensity) +/- StdErr**

**Variability Gauge Rank=363, protein_id=IPI00009841.4, Annotation=CDNA_FLJ31747_fis,_clone_NT2RI2007377,_highly_similar_to_RNA-BINDING_PROTEIN_EWS**

**Variability Chart for Mean Log2(Intensity) +/- StdErr**

**Variability Gauge Rank=364, protein_id=IPI00007423.1, Annotation=Isoform_1_of_Acidic_leucine-rich_nuclear_phosphoprotein_32_family_member_B**

**Variability Chart for Mean Log2(Intensity) +/- StdErr**

**Variability Gauge Rank=365, protein_id=IPI00220487.3, Annotation=ATP_synthase,_H+_transporting,_mitochondrial_F0_complex,_subunit_d_isoform_a**

**Variability Chart for Mean Log2(Intensity) +/- StdErr**

**Variability Gauge Rank=366, protein_id=IPI00010153.5, Annotation=60S_ribosomal_protein_L23**

**Variability Chart for Mean Log2(Intensity) +/- StdErr**

**Variability Gauge Rank=367, protein_id=IPI00021785.2, Annotation=Cytochrome_c_oxidase_polypeptide_Vb,_mitochondrial_precursor**

**Variability Chart for Mean Log2(Intensity) +/- StdErr**

**Variability Gauge Rank=368, protein_id=IPI00293817.3, Annotation=Gamma-soluble_NSF_attachment_protein**

**Variability Chart for Mean Log2(Intensity) +/- StdErr**

**Variability Gauge Rank=369, protein_id=IPI00003925.5, Annotation=Isoform_1_of_Pyruvate_dehydrogenase_E1_component_subunit_beta,_mitochondrial_precursor**

**Variability Chart for Mean Log2(Intensity) +/- StdErr**

**Variability Gauge Rank=370, protein_id=IPI00329801.11, Annotation=Annexin_A5**

**Variability Chart for Mean Log2(Intensity) +/- StdErr**

**Variability Gauge Rank=371, protein_id=IPI00033494.3, Annotation=Myosin_regulatory_light_chain**

**Variability Chart for Mean Log2(Intensity) +/- StdErr**

**Variability Gauge Rank=372, protein_id=IPI00176662.1, Annotation=9_kDa_protein**

**Variability Chart for Mean Log2(Intensity) +/- StdErr**

**Variability Gauge Rank=373, protein_id=IPI00008438.1, Annotation=40S_ribosomal_protein_S10**

**Variability Chart for Mean Log2(Intensity) +/- StdErr**

**Variability Gauge Rank=374, protein_id=IPI00412579.5, Annotation=60S_ribosomal_protein_L10a**

**Variability Chart for Mean Log2(Intensity) +/- StdErr**

**Variability Gauge Rank=375, protein_id=IPI00016801.1, Annotation=Glutamate_dehydrogenase_1,_mitochondrial_precursor**

**Variability Chart for Mean Log2(Intensity) +/- StdErr**

**Variability Gauge Rank=376, protein_id=IPI00413728.3, Annotation=Isoform_1_of_Spectrin_alpha_chain,_brain**

**Variability Chart for Mean Log2(Intensity) +/- StdErr**

**Variability Gauge Rank=377, protein_id=280813, Annotation=B36203_iron-responsive_element-binding_protein_(clone_10.1)_-_human**

**Variability Chart for Mean Log2(Intensity) +/- StdErr**

**Variability Gauge Rank=378, protein_id=IPI00015550.6, Annotation=Prothymosin_alpha**

**Variability Chart for Mean Log2(Intensity) +/- StdErr**

**Variability Gauge Rank=379, protein_id=IPI00176678.9, Annotation=PREDICTED:_similar_to_Alcohol_dehydrogenase_class_3_chi_chain_(Alcohol_dehydrogenase_class_III_chi_chain)_(S-(hydroxymethyl)glutathione_dehydrogenase)_(Glutathione-dependent_formaldehyde_dehydrogenase)_(FDH)_isoform_1**

**Variability Chart for Mean Log2(Intensity) +/- StdErr**

**Variability Gauge Rank=380, protein_id=IPI00005159.3, Annotation=Actin-like_protein_2**

**Variability Chart for Mean Log2(Intensity) +/- StdErr**

**Variability Gauge Rank=381, protein_id=IPI00216587.8, Annotation=40S_ribosomal_protein_S8**

**Variability Chart for Mean Log2(Intensity) +/- StdErr**

**Variability Gauge Rank=382, protein_id=IPI00027497.4, Annotation=Glucose-6-phosphate_isomerase**

**Variability Chart for Mean Log2(Intensity) +/- StdErr**

**Variability Gauge Rank=383, protein_id=IPI00017334.1, Annotation=Prohibitin**

**Variability Chart for Mean Log2(Intensity) +/- StdErr**

**Variability Gauge Rank=384, protein_id=IPI00011416.2, Annotation=Delta3,5-delta2,4-dienoyl-CoA_isomerase,_mitochondrial_precursor**

**Variability Chart for Mean Log2(Intensity) +/- StdErr**

**Variability Gauge Rank=385, protein_id=34303898, Annotation=KIAA0357_[Homo_sapiens]**

**Variability Chart for Mean Log2(Intensity) +/- StdErr**

**Variability Gauge Rank=386, protein_id=IPI00218667.2, Annotation=Stathmin-2**

**Variability Chart for Mean Log2(Intensity) +/- StdErr**

**Variability Gauge Rank=387, protein_id=IPI00217471.2, Annotation=Hemoglobin_subunit_epsilon**

**Variability Chart for Mean Log2(Intensity) +/- StdErr**

**Variability Gauge Rank=388, protein_id=IPI00473011.2, Annotation=Hemoglobin_subunit_delta**

**Variability Chart for Mean Log2(Intensity) +/- StdErr**

**Variability Gauge Rank=389, protein_id=IPI00012345.2, Annotation=Isoform_SRP55-1_of_Splicing_factor,_arginine/serine-rich_6**

**Variability Chart for Mean Log2(Intensity) +/- StdErr**

**Variability Gauge Rank=390, protein_id=IPI00002459.4, Annotation=annexin_VI_isoform_2**

**Variability Chart for Mean Log2(Intensity) +/- StdErr**

**Variability Gauge Rank=391, protein_id=IPI00031288.1, Annotation=Claudin-14**

**Variability Chart for Mean Log2(Intensity) +/- StdErr**

**Variability Gauge Rank=392, protein_id=IPI00397828.2, Annotation=20_kDa_protein**

**Variability Chart for Mean Log2(Intensity) +/- StdErr**

**Variability Gauge Rank=393, protein_id=IPI00248321.8, Annotation=PREDICTED:_similar_to_peptidylprolyl_isomerase_A_isoform_1**

**Variability Chart for Mean Log2(Intensity) +/- StdErr**

**Variability Gauge Rank=394, protein_id=IPI00299584.3, Annotation=Isoform_1_of_Tripartite_motif-containing_protein_15**

**Variability Chart for Mean Log2(Intensity) +/- StdErr**

**Variability Gauge Rank=395, protein_id=IPI00016387.4, Annotation=pre-mRNA_cleavage_complex_II_protein_Pcf11**

**Variability Chart for Mean Log2(Intensity) +/- StdErr**

**Variability Gauge Rank=396, protein_id=IPI00011268.2, Annotation=RNA_binding_protein_(Fragment)**

**Variability Chart for Mean Log2(Intensity) +/- StdErr**

**Variability Gauge Rank=397, protein_id=IPI00021836.1, Annotation=Isoform_1_of_Prorelaxin_H2_precursor**

**Variability Chart for Mean Log2(Intensity) +/- StdErr**

**Variability Gauge Rank=398, protein_id=IPI00008529.1, Annotation=60S_acidic_ribosomal_protein_P2**

**Variability Chart for Mean Log2(Intensity) +/- StdErr**

**Variability Gauge Rank=399, protein_id=IPI00219155.4, Annotation=60S_ribosomal_protein_L27**

**Variability Chart for Mean Log2(Intensity) +/- StdErr**

**Variability Gauge Rank=400, protein_id=IPI00008433.3, Annotation=40S_ribosomal_protein_S5**

**Variability Chart for Mean Log2(Intensity) +/- StdErr**

**Variability Gauge Rank=401, protein_id=IPI00011913.1, Annotation=Heterogeneous_nuclear_ribonucleoprotein_A0**

**Variability Chart for Mean Log2(Intensity) +/- StdErr**

**Variability Gauge Rank=402, protein_id=IPI00031522.2, Annotation=Trifunctional_enzyme_subunit_alpha,_mitochondrial_precursor**

**Variability Chart for Mean Log2(Intensity) +/- StdErr**

**Variability Gauge Rank=403, protein_id=IPI00148061.3, Annotation=L-lactate_dehydrogenase_A-like_6A**

**Variability Chart for Mean Log2(Intensity) +/- StdErr**

**Variability Gauge Rank=404, protein_id=IPI00013495.1, Annotation=Isoform_2_of_ATP-binding_cassette_sub-family_F_member_1**

**Variability Chart for Mean Log2(Intensity) +/- StdErr**

**Variability Gauge Rank=405, protein_id=IPI00642673.1, Annotation=ATP-binding_cassette,_sub-family_F_(GCN20),_member_1**

**Variability Chart for Mean Log2(Intensity) +/- StdErr**

**Variability Gauge Rank=406, protein_id=16974825, Annotation=A_Chain_A,_Solution_Structure_Of_Calcium-Calmodulin_N-Terminal_Domain**

**Variability Chart for Mean Log2(Intensity) +/- StdErr**

**Variability Gauge Rank=407, protein_id=IPI00012918.5, Annotation=AT_rich_interactive_domain_5A_isoform_1**

**Variability Chart for Mean Log2(Intensity) +/- StdErr**

**Variability Gauge Rank=408, protein_id=IPI00288940.5, Annotation=Isoform_1_of_Obscurin**

**Variability Chart for Mean Log2(Intensity) +/- StdErr**

**Variability Gauge Rank=409, protein_id=IPI00328587.4, Annotation=ENO1P_protein**

**Variability Chart for Mean Log2(Intensity) +/- StdErr**

**Variability Gauge Rank=410, protein_id=IPI00021924.1, Annotation=Histone_H1x**

**Variability Chart for Mean Log2(Intensity) +/- StdErr**

**Variability Gauge Rank=411, protein_id=IPI00024919.3, Annotation=Thioredoxin-dependent_peroxide_reductase,_mitochondrial_precursor**

**Variability Chart for Mean Log2(Intensity) +/- StdErr**

**Variability Gauge Rank=412, protein_id=IPI00007385.5, Annotation=Uncharacterized_protein_C16orf7**

**Variability Chart for Mean Log2(Intensity) +/- StdErr**

**Variability Gauge Rank=413, protein_id=IPI00024320.1, Annotation=Putative_RNA-binding_protein_3**

**Variability Chart for Mean Log2(Intensity) +/- StdErr**

**Variability Gauge Rank=414, protein_id=IPI00413778.7, Annotation=FKBP1A_protein**

**Variability Chart for Mean Log2(Intensity) +/- StdErr**

**Variability Gauge Rank=415, protein_id=IPI00009992.1, Annotation=Centaurin-alpha_1**

**Variability Chart for Mean Log2(Intensity) +/- StdErr**

**Variability Gauge Rank=416, protein_id=IPI00554769.1, Annotation=Mutant_GSTP1**

**Variability Chart for Mean Log2(Intensity) +/- StdErr**

**Variability Gauge Rank=417, protein_id=IPI00003419.1, Annotation=Small_acidic_protein**

**Variability Chart for Mean Log2(Intensity) +/- StdErr**

**Variability Gauge Rank=418, protein_id=IPI00783538.1, Annotation=Zinc-finger_homeodomain_protein_4**

**Variability Chart for Mean Log2(Intensity) +/- StdErr**

**Variability Gauge Rank=419, protein_id=IPI00220301.4, Annotation=Peroxiredoxin-6**

**Variability Chart for Mean Log2(Intensity) +/- StdErr**

**Variability Gauge Rank=420, protein_id=IPI00017025.3, Annotation=PREDICTED:_similar_to_peptidylprolyl_isomerase_A_isoform_1**

**Variability Chart for Mean Log2(Intensity) +/- StdErr**

**Variability Gauge Rank=421, protein_id=IPI00106604.4, Annotation=PREDICTED:_similar_to_peptidylprolyl_isomerase_A_isoform_1**

**Variability Chart for Mean Log2(Intensity) +/- StdErr**

**Variability Gauge Rank=422, protein_id=2760897, Annotation=natural_killer_cell_inhibitory_receptor_KIR2DL3_variant_[Homo_sapiens]**

**Variability Chart for Mean Log2(Intensity) +/- StdErr**

**Variability Gauge Rank=423, protein_id=IPI00395601.1, Annotation=Synaptotagmin-10**

**Variability Chart for Mean Log2(Intensity) +/- StdErr**

**Variability Gauge Rank=424, protein_id=IPI00328840.8, Annotation=THO_complex_subunit_4**

**Variability Chart for Mean Log2(Intensity) +/- StdErr**

**Variability Gauge Rank=425, protein_id=IPI00216085.2, Annotation=Cytochrome_c_oxidase_subunit_VIb_isoform_1**

**Variability Chart for Mean Log2(Intensity) +/- StdErr**

**Variability Gauge Rank=426, protein_id=IPI00011675.1, Annotation=Isoform_Sp100-HMG_of_Nuclear_autoantigen_Sp-100**

**Variability Chart for Mean Log2(Intensity) +/- StdErr**

**Variability Gauge Rank=427, protein_id=IPI00291922.2, Annotation=Proteasome_subunit_alpha_type_5**

**Variability Chart for Mean Log2(Intensity) +/- StdErr**

**Variability Gauge Rank=428, protein_id=IPI00031556.5, Annotation=Splicing_factor_U2AF_65_kDa_subunit**

**Variability Chart for Mean Log2(Intensity) +/- StdErr**

**Variability Gauge Rank=429, protein_id=IPI00438229.1, Annotation=tripartite_motif-containing_28_protein**

**Variability Chart for Mean Log2(Intensity) +/- StdErr**

**Variability Gauge Rank=430, protein_id=IPI00216298.5, Annotation=Thioredoxin**

**Variability Chart for Mean Log2(Intensity) +/- StdErr**

**Variability Gauge Rank=431, protein_id=IPI00013826.2, Annotation=Isoform_1_of_Atrial_natriuretic_peptide_clearance_receptor_precursor**

**Variability Chart for Mean Log2(Intensity) +/- StdErr**

**Variability Gauge Rank=432, protein_id=IPI00297779.6, Annotation=T-complex_protein_1_subunit_beta**

**Variability Chart for Mean Log2(Intensity) +/- StdErr**

**Variability Gauge Rank=433, protein_id=IPI00216125.6, Annotation=SRP9_protein**

**Variability Chart for Mean Log2(Intensity) +/- StdErr**

**Variability Gauge Rank=434, protein_id=IPI00012094.1, Annotation=Isoform_Long_of_Probable_ubiquitin_carboxyl-terminal_hydrolase_FAF-Y**

**Variability Chart for Mean Log2(Intensity) +/- StdErr**

**Variability Gauge Rank=435, protein_id=IPI00020984.1, Annotation=Calnexin_precursor**

**Variability Chart for Mean Log2(Intensity) +/- StdErr**

**Variability Gauge Rank=436, protein_id=IPI00328319.7, Annotation=Histone-binding_protein_RBBP4**

**Variability Chart for Mean Log2(Intensity) +/- StdErr**

**Variability Gauge Rank=437, protein_id=IPI00006510.1, Annotation=Tubulin_beta-1_chain**

**Variability Chart for Mean Log2(Intensity) +/- StdErr**

**Variability Gauge Rank=438, protein_id=IPI00010320.1, Annotation=Chromobox_protein_homolog_1**

**Variability Chart for Mean Log2(Intensity) +/- StdErr**

**Variability Gauge Rank=439, protein_id=IPI00550689.3, Annotation=HSPC117_protein**

**Variability Chart for Mean Log2(Intensity) +/- StdErr**

**Variability Gauge Rank=440, protein_id=IPI00027165.3, Annotation=Isoform_R-type_of_Pyruvate_kinase_isozymes_R/L**

**Variability Chart for Mean Log2(Intensity) +/- StdErr**

**Variability Gauge Rank=441, protein_id=IPI00017763.4, Annotation=Nucleosome_assembly_protein_1-like_4**

**Variability Chart for Mean Log2(Intensity) +/- StdErr**

**Variability Gauge Rank=442, protein_id=IPI00029266.1, Annotation=Small_nuclear_ribonucleoprotein_E**

**Variability Chart for Mean Log2(Intensity) +/- StdErr**

**Variability Gauge Rank=443, protein_id=IPI00290770.3, Annotation=chaperonin_containing_TCP1,_subunit_3_isoform_b**

**Variability Chart for Mean Log2(Intensity) +/- StdErr**

**Variability Gauge Rank=444, protein_id=IPI00298237.7, Annotation=Isoform_1_of_Tripeptidyl-peptidase_1_precursor**

**Variability Chart for Mean Log2(Intensity) +/- StdErr**

**Variability Gauge Rank=445, protein_id=IPI00216139.2, Annotation=Isoform_I_of_Septin-6**

**Variability Chart for Mean Log2(Intensity) +/- StdErr**

**Variability Gauge Rank=446, protein_id=IPI00013769.1, Annotation=Alpha-enolase,_lung_specific**

**Variability Chart for Mean Log2(Intensity) +/- StdErr**

**Variability Gauge Rank=447, protein_id=IPI00419237.2, Annotation=leucine_aminopeptidase_3**

**Variability Chart for Mean Log2(Intensity) +/- StdErr**

**Variability Gauge Rank=448, protein_id=IPI00026202.1, Annotation=60S_ribosomal_protein_L18a**

**Variability Chart for Mean Log2(Intensity) +/- StdErr**

**Variability Gauge Rank=449, protein_id=IPI00221092.7, Annotation=40S_ribosomal_protein_S16**

**Variability Chart for Mean Log2(Intensity) +/- StdErr**

**Variability Gauge Rank=450, protein_id=IPI00017448.1, Annotation=40S_ribosomal_protein_S21**

**Variability Chart for Mean Log2(Intensity) +/- StdErr**

**Variability Gauge Rank=451, protein_id=IPI00215911.2, Annotation=DNA-(apurinic_or_apyrimidinic_site)_lyase**

**Variability Chart for Mean Log2(Intensity) +/- StdErr**

**Variability Gauge Rank=452, protein_id=IPI00052885.6, Annotation=PREDICTED:_similar_to_60S_ribosomal_protein_L29**

**Variability Chart for Mean Log2(Intensity) +/- StdErr**

**Variability Gauge Rank=453, protein_id=IPI00174347.5, Annotation=Novel_protein**

**Variability Chart for Mean Log2(Intensity) +/- StdErr**

**Variability Gauge Rank=454, protein_id=IPI00022145.5, Annotation=Nuclear_ubiquitous_casein_and_cyclin-dependent_kinases_substrate**

**Variability Chart for Mean Log2(Intensity) +/- StdErr**

**Variability Gauge Rank=455, protein_id=IPI00218539.3, Annotation=Isoform_B_of_Collagen_alpha-1(XI)_chain_precursor**

**Variability Chart for Mean Log2(Intensity) +/- StdErr**

**Variability Gauge Rank=456, protein_id=IPI00008669.2, Annotation=38_kDa_protein**

**Variability Chart for Mean Log2(Intensity) +/- StdErr**

**Variability Gauge Rank=457, protein_id=IPI00075558.8, Annotation=PREDICTED:_similar_to_60S_ribosomal_protein_L7a**

**Variability Chart for Mean Log2(Intensity) +/- StdErr**

**Variability Gauge Rank=458, protein_id=IPI00328978.2, Annotation=Cathepsin_W_precursor**

**Variability Chart for Mean Log2(Intensity) +/- StdErr**

**Variability Gauge Rank=459, protein_id=IPI00008274.5, Annotation=Adenylyl_cyclase-associated_protein_1**

**Variability Chart for Mean Log2(Intensity) +/- StdErr**

**Variability Gauge Rank=460, protein_id=IPI00026271.4, Annotation=40S_ribosomal_protein_S14**

**Variability Chart for Mean Log2(Intensity) +/- StdErr**

**Variability Gauge Rank=461, protein_id=IPI00299048.4, Annotation=IQ_motif_containing_GTPase_activating_protein_2**

**Variability Chart for Mean Log2(Intensity) +/- StdErr**

**Variability Gauge Rank=462, protein_id=IPI00023748.3, Annotation=Nascent_polypeptide-associated_complex_subunit_alpha**

**Variability Chart for Mean Log2(Intensity) +/- StdErr**

**Variability Gauge Rank=463, protein_id=IPI00006579.1, Annotation=Cytochrome_c_oxidase_subunit_4_isoform_1,_mitochondrial_precursor**

**Variability Chart for Mean Log2(Intensity) +/- StdErr**

**Variability Gauge Rank=464, protein_id=IPI00022463.1, Annotation=Serotransferrin_precursor**

**Variability Chart for Mean Log2(Intensity) +/- StdErr**

**Variability Gauge Rank=465, protein_id=IPI00465044.2, Annotation=Protein_RCC2**

**Variability Chart for Mean Log2(Intensity) +/- StdErr**

**Variability Gauge Rank=466, protein_id=IPI00103525.1, Annotation=paraspeckle_protein_1_isoform_alpha**

**Variability Chart for Mean Log2(Intensity) +/- StdErr**

**Variability Gauge Rank=467, protein_id=IPI00024053.3, Annotation=ImmunoglobulIn_superfamIly,_member_9**

**Variability Chart for Mean Log2(Intensity) +/- StdErr**

**Variability Gauge Rank=468, protein_id=IPI00219425.3, Annotation=Isoform_Beta_of_Poliovirus_receptor_precursor**

**Variability Chart for Mean Log2(Intensity) +/- StdErr**

**Variability Gauge Rank=469, protein_id=14494990, Annotation=OTTHUMP00000045529_[Homo_sapiens]**

**Variability Chart for Mean Log2(Intensity) +/- StdErr**

**Variability Gauge Rank=470, protein_id=IPI00301277.1, Annotation=Heat_shock_70_kDa_protein_1L**

**Variability Chart for Mean Log2(Intensity) +/- StdErr**

**Variability Gauge Rank=471, protein_id=IPI00005859.1, Annotation=Cytokeratin_type_II**

**Variability Chart for Mean Log2(Intensity) +/- StdErr**

**Variability Gauge Rank=472, protein_id=IPI00302592.2, Annotation=Filamin_A,_alpha**

**Variability Chart for Mean Log2(Intensity) +/- StdErr**

**Variability Gauge Rank=473, protein_id=IPI00069750.1, Annotation=fuse-binding_protein-interacting_repressor_isoform_a**

**Variability Chart for Mean Log2(Intensity) +/- StdErr**

**Variability Gauge Rank=474, protein_id=IPI00032304.1, Annotation=Plastin-1**

**Variability Chart for Mean Log2(Intensity) +/- StdErr**

**Variability Gauge Rank=475, protein_id=IPI00410693.3, Annotation=Isoform_1_of_Plasminogen_activator_inhibitor_1_RNA-binding_protein**

**Variability Chart for Mean Log2(Intensity) +/- StdErr**

**Variability Gauge Rank=476, protein_id=IPI00643041.2, Annotation=GTP-binding_nuclear_protein_Ran**

**Variability Chart for Mean Log2(Intensity) +/- StdErr**

**Variability Gauge Rank=477, protein_id=IPI00745136.2, Annotation=PREDICTED:_similar_to_peptidylprolyl_isomerase_A_isoform_1**

**Variability Chart for Mean Log2(Intensity) +/- StdErr**

**Variability Gauge Rank=478, protein_id=IPI00045837.4, Annotation=Protein_phosphatase_1,_regulatory_subunit_15B_variant**

**Variability Chart for Mean Log2(Intensity) +/- StdErr**

**Variability Gauge Rank=479, protein_id=IPI00644597.1, Annotation=Hypothetical_protein**

**Variability Chart for Mean Log2(Intensity) +/- StdErr**

**Variability Gauge Rank=480, protein_id=IPI00007144.1, Annotation=60S_ribosomal_protein_L26-like_1**

**Variability Chart for Mean Log2(Intensity) +/- StdErr**

**Variability Gauge Rank=481, protein_id=IPI00027270.1, Annotation=60S_ribosomal_protein_L26**

**Variability Chart for Mean Log2(Intensity) +/- StdErr**

**Variability Gauge Rank=482, protein_id=IPI00102300.3, Annotation=Isoform_3_of_Platelet_glycoprotein_VI_precursor**

**Variability Chart for Mean Log2(Intensity) +/- StdErr**

**Variability Gauge Rank=483, protein_id=IPI00419647.2, Annotation=Isoform_1_of_Protein_patched_homolog_2**

**Variability Chart for Mean Log2(Intensity) +/- StdErr**

**Variability Gauge Rank=484, protein_id=IPI00026174.1, Annotation=Cholecystokinins_precursor**

**Variability Chart for Mean Log2(Intensity) +/- StdErr**

**Variability Gauge Rank=485, protein_id=IPI00023283.3, Annotation=Isoform_2_of_Titin**

**Variability Chart for Mean Log2(Intensity) +/- StdErr**

**Variability Gauge Rank=486, protein_id=IPI00014186.3, Annotation=Isoform_A_of_Alpha-fetoprotein_enhancer-binding_protein**

**Variability Chart for Mean Log2(Intensity) +/- StdErr**

**Variability Gauge Rank=487, protein_id=IPI00065280.2, Annotation=Protein_C21orf129**

**Variability Chart for Mean Log2(Intensity) +/- StdErr**

**Variability Gauge Rank=488, protein_id=IPI00306239.4, Annotation=Katanin_p80_WD40-containing_subunit_B1**

**Variability Chart for Mean Log2(Intensity) +/- StdErr**

**Variability Gauge Rank=489, protein_id=IPI00217952.6, Annotation=Isoform_1_of_Glucosamine--fructose-6-phosphate_aminotransferase_[isomerizing]_1**

**Variability Chart for Mean Log2(Intensity) +/- StdErr**

**Variability Gauge Rank=490, protein_id=IPI00470682.4, Annotation=Arginyl_aminopeptidase-like_1**

**Variability Chart for Mean Log2(Intensity) +/- StdErr**

**Variability Gauge Rank=491, protein_id=IPI00292674.1, Annotation=WD_repeat_domain_21A_isoform_2**

**Variability Chart for Mean Log2(Intensity) +/- StdErr**

**Variability Gauge Rank=492, protein_id=IPI00748219.1, Annotation=Similar_to_Glyceraldehyde-3-phosphate_dehydrogenase,_liver**

**Variability Chart for Mean Log2(Intensity) +/- StdErr**

**Variability Gauge Rank=493, protein_id=7661818, Annotation=hypothetical_protein_LOC29094_[Homo_sapiens]**

**Variability Chart for Mean Log2(Intensity) +/- StdErr**

**Variability Gauge Rank=494, protein_id=IPI00087498.4, Annotation=N-acetyltransferase_ESCO2**

**Variability Chart for Mean Log2(Intensity) +/- StdErr**

**Variability Gauge Rank=495, protein_id=IPI00235481.2, Annotation=PMFBP1_protein**

**Variability Chart for Mean Log2(Intensity) +/- StdErr**

**Variability Gauge Rank=496, protein_id=IPI00292221.3, Annotation=Ribosome_production_factor_1**

**Variability Chart for Mean Log2(Intensity) +/- StdErr**

**Variability Gauge Rank=497, protein_id=IPI00328285.5, Annotation=Ubiquitin_carboxyl-terminal_hydrolase_20**

**Variability Chart for Mean Log2(Intensity) +/- StdErr**

**Variability Gauge Rank=498, protein_id=IPI00418497.1, Annotation=Isoform_2_of_Import_inner_membrane_translocase_subunit_TIM50,_mitochondrial_precursor**

**Variability Chart for Mean Log2(Intensity) +/- StdErr**

**Variability Gauge Rank=499, protein_id=IPI00219221.2, Annotation=Galectin-7**

**Variability Chart for Mean Log2(Intensity) +/- StdErr**

**Variability Gauge Rank=500, protein_id=IPI00386040.1, Annotation=Hypothetical_protein_DKFZp547D0916**

**Variability Chart for Mean Log2(Intensity) +/- StdErr**

**Variability Gauge Rank=501, protein_id=IPI00025400.1, Annotation=Granzyme_H_precursor**

**Variability Chart for Mean Log2(Intensity) +/- StdErr**

**Variability Gauge Rank=502, protein_id=IPI00154558.1, Annotation=Zinc_finger_protein_420**

**Variability Chart for Mean Log2(Intensity) +/- StdErr**

**Variability Gauge Rank=503, protein_id=IPI00376833.1, Annotation=Mitochondrial_carrier_triple_repeat_6**

**Variability Chart for Mean Log2(Intensity) +/- StdErr**

**Variability Gauge Rank=504, protein_id=IPI00178953.3, Annotation=Isoform_4_of_Zinc_finger_protein_638**

**Variability Chart for Mean Log2(Intensity) +/- StdErr**

**Variability Gauge Rank=505, protein_id=IPI00013296.2, Annotation=40S_ribosomal_protein_S18**

**Variability Chart for Mean Log2(Intensity) +/- StdErr**

**Variability Gauge Rank=506, protein_id=IPI00292734.4, Annotation=Homeobox_protein_Hox-D9**

**Variability Chart for Mean Log2(Intensity) +/- StdErr**

**Variability Gauge Rank=507, protein_id=IPI00152815.6, Annotation=VPPP1921**

**Variability Chart for Mean Log2(Intensity) +/- StdErr**

**Variability Gauge Rank=508, protein_id=IPI00479337.2, Annotation=PLSS3001**

**Variability Chart for Mean Log2(Intensity) +/- StdErr**

**Variability Gauge Rank=509, protein_id=IPI00003907.1, Annotation=Isoform_1_of_Protocadherin_gamma_C5_precursor**

**Variability Chart for Mean Log2(Intensity) +/- StdErr**

**Variability Gauge Rank=510, protein_id=IPI00457338.2, Annotation=PREDICTED:_similar_to_60S_ribosomal_protein_L6**

**Variability Chart for Mean Log2(Intensity) +/- StdErr**

**Variability Gauge Rank=511, protein_id=IPI00787593.1, Annotation=PREDICTED:_similar_to_CG33300-PA**

**Variability Chart for Mean Log2(Intensity) +/- StdErr**

**Variability Gauge Rank=512, protein_id=IPI00016763.3, Annotation=GTP-binding_protein**

**Variability Chart for Mean Log2(Intensity) +/- StdErr**

**Variability Gauge Rank=513, protein_id=IPI00443024.1, Annotation=Hypothetical_protein_FLJ26089**

**Variability Chart for Mean Log2(Intensity) +/- StdErr**

**Variability Gauge Rank=514, protein_id=IPI00556589.3, Annotation=PREDICTED:_similar_to_40S_ribosomal_protein_S28**

**Variability Chart for Mean Log2(Intensity) +/- StdErr**

**Variability Gauge Rank=515, protein_id=IPI00027280.2, Annotation=Isoform_Beta-2_of_DNA_topoisomerase_2-beta**

**Variability Chart for Mean Log2(Intensity) +/- StdErr**

**Variability Gauge Rank=516, protein_id=IPI00382989.2, Annotation=Isoform_1_of_Protein_C7orf10**

**Variability Chart for Mean Log2(Intensity) +/- StdErr**

**Variability Gauge Rank=517, protein_id=IPI00306523.3, Annotation=zinc_finger,_matrin_type_1_isoform_1**

**Variability Chart for Mean Log2(Intensity) +/- StdErr**

**Variability Gauge Rank=518, protein_id=IPI00175098.6, Annotation=PREDICTED:_similar_to_ribosomal_protein_L10**

**Variability Chart for Mean Log2(Intensity) +/- StdErr**

**Variability Gauge Rank=519, protein_id=IPI00001513.1, Annotation=Isoform_1_of_Protocadherin_alpha_10_precursor**

**Variability Chart for Mean Log2(Intensity) +/- StdErr**

**Variability Gauge Rank=520, protein_id=IPI00064892.1, Annotation=Lysozyme-like_protein_4_precursor**

**Variability Chart for Mean Log2(Intensity) +/- StdErr**

**Variability Gauge Rank=521, protein_id=IPI00018353.2, Annotation=Isoform_1_of_Cohesin_subunit_SA-3**

**Variability Chart for Mean Log2(Intensity) +/- StdErr**

**Variability Gauge Rank=522, protein_id=IPI00167941.1, Annotation=Midasin**

**Variability Chart for Mean Log2(Intensity) +/- StdErr**

**Variability Gauge Rank=523, protein_id=IPI00003391.4, Annotation=305_kDa_protein**

**Variability Chart for Mean Log2(Intensity) +/- StdErr**

**Variability Gauge Rank=524, protein_id=IPI00743314.1, Annotation=General_vesicular_transport_factor_p115**

**Variability Chart for Mean Log2(Intensity) +/- StdErr**

**Variability Gauge Rank=525, protein_id=IPI00017423.1, Annotation=Isoform_2_of_Centrosomal_protein_of_135_kDa**

**Variability Chart for Mean Log2(Intensity) +/- StdErr**

**Variability Gauge Rank=526, protein_id=IPI00748285.1, Annotation=Similar_to_Keratin,_type_II_cytoskeletal_8**

**Variability Chart for Mean Log2(Intensity) +/- StdErr**

**Variability Gauge Rank=527, protein_id=IPI00306043.1, Annotation=Isoform_1_of_YTH_domain_family_protein_2**

**Variability Chart for Mean Log2(Intensity) +/- StdErr**

**Variability Gauge Rank=528, protein_id=1877499, Annotation=polyhomeotic_1_homolog_[Homo_sapiens]**

**Variability Chart for Mean Log2(Intensity) +/- StdErr**

**Variability Gauge Rank=529, protein_id=IPI00302962.1, Annotation=Amphiphysin_I_variant_CT4_(Fragment)**

**Variability Chart for Mean Log2(Intensity) +/- StdErr**

**Variability Gauge Rank=530, protein_id=IPI00084684.8, Annotation=PREDICTED:_similar_to_Zinc_finger_protein_469**

**Variability Chart for Mean Log2(Intensity) +/- StdErr**

**Variability Gauge Rank=531, protein_id=IPI00164623.4, Annotation=187_kDa_protein**

**Variability Chart for Mean Log2(Intensity) +/- StdErr**

**Variability Gauge Rank=532, protein_id=IPI00235647.6, Annotation=PREDICTED:_similar_to_fibrillarin**

**Variability Chart for Mean Log2(Intensity) +/- StdErr**

**Variability Gauge Rank=533, protein_id=IPI00012899.1, Annotation=Cytokine_receptor_common_gamma_chain_precursor**

**Variability Chart for Mean Log2(Intensity) +/- StdErr**

**Variability Gauge Rank=534, protein_id=IPI00024502.2, Annotation=Ubiquilin-4**

**Variability Chart for Mean Log2(Intensity) +/- StdErr**

**Variability Gauge Rank=535, protein_id=IPI00186970.5, Annotation=Hypothetical_protein_RP11-293F5.5**

**Variability Chart for Mean Log2(Intensity) +/- StdErr**

**Variability Gauge Rank=536, protein_id=IPI00009032.1, Annotation=Lupus_La_protein**

**Variability Chart for Mean Log2(Intensity) +/- StdErr**

**Variability Gauge Rank=537, protein_id=IPI00215719.5, Annotation=60S_ribosomal_protein_L18**

**Variability Chart for Mean Log2(Intensity) +/- StdErr**

**Variability Gauge Rank=538, protein_id=IPI00658079.1, Annotation=Isoform_2_of_WD_repeat_protein_48**

**Variability Chart for Mean Log2(Intensity) +/- StdErr**

**Variability Gauge Rank=539, protein_id=IPI00221285.5, Annotation=ankyrin_repeat_domain_50**

**Variability Chart for Mean Log2(Intensity) +/- StdErr**

**Variability Gauge Rank=540, protein_id=IPI00000201.2, Annotation=Isoform_1_of_Low-density_lipoprotein_receptor-related_protein_3_precursor**

**Variability Chart for Mean Log2(Intensity) +/- StdErr**

**Variability Gauge Rank=541, protein_id=IPI00020749.5, Annotation=Isoform_2_of_Testis-expressed_protein_101_precursor**

**Variability Chart for Mean Log2(Intensity) +/- StdErr**

**Variability Gauge Rank=542, protein_id=IPI00017443.3, Annotation=absent_in_melanoma_1-like**

**Variability Chart for Mean Log2(Intensity) +/- StdErr**

**Variability Gauge Rank=543, protein_id=IPI00737969.2, Annotation=PREDICTED:_similar_to_Protein_MICAL-3**

**Variability Chart for Mean Log2(Intensity) +/- StdErr**

**Variability Gauge Rank=544, protein_id=IPI00427502.1, Annotation=Isoform_2_of_Protein_D11Lgp1_homolog_precursor**

**Variability Chart for Mean Log2(Intensity) +/- StdErr**

**Variability Gauge Rank=545, protein_id=IPI00337741.4, Annotation=Acylamino-acid-releasing_enzyme**

**Variability Chart for Mean Log2(Intensity) +/- StdErr**

**Variability Gauge Rank=546, protein_id=IPI00748026.1, Annotation=Conserved_hypothetical_protein**

**Variability Chart for Mean Log2(Intensity) +/- StdErr**

**Variability Gauge Rank=547, protein_id=IPI00022479.4, Annotation=guanine_nucleotide_exchange_factor_p532**

**Variability Chart for Mean Log2(Intensity) +/- StdErr**

**Variability Gauge Rank=548, protein_id=IPI00002353.1, Annotation=KIAA1318_protein_(Fragment)**

**Variability Chart for Mean Log2(Intensity) +/- StdErr**

**Variability Gauge Rank=549, protein_id=IPI00004657.1, Annotation=HLA_class_I_histocompatibility_antigen,_B-7_alpha_chain_precursor**

**Variability Chart for Mean Log2(Intensity) +/- StdErr**

**Variability Gauge Rank=550, protein_id=IPI00235842.2, Annotation=Zinc_finger_protein_483**

**Variability Chart for Mean Log2(Intensity) +/- StdErr**

**Variability Gauge Rank=551, protein_id=IPI00168180.1, Annotation=Isoform_1_of_Calcyphosin-2**

**Variability Chart for Mean Log2(Intensity) +/- StdErr**

**Variability Gauge Rank=552, protein_id=434755, Annotation=KIAA0123_[Homo_sapiens]**

**Variability Chart for Mean Log2(Intensity) +/- StdErr**

**Variability Gauge Rank=553, protein_id=219414, Annotation=arylamine_N-acetyltransferase_[Homo_sapiens]**

**Variability Chart for Mean Log2(Intensity) +/- StdErr**

**Variability Gauge Rank=554, protein_id=IPI00219740.3, Annotation=Isoform_2_of_DNA_replication_licensing_factor_MCM7**

**Variability Chart for Mean Log2(Intensity) +/- StdErr**

**Variability Gauge Rank=555, protein_id=IPI00012543.1, Annotation=Isoform_1_of_Protocadherin_gamma_A8_precursor**

**Variability Chart for Mean Log2(Intensity) +/- StdErr**

**Variability Gauge Rank=556, protein_id=IPI00304587.3, Annotation=dispatched_B**

**Variability Chart for Mean Log2(Intensity) +/- StdErr**

**Variability Gauge Rank=557, protein_id=7106878, Annotation=AF151078_1_HSPC244_[Homo_sapiens]**

**Variability Chart for Mean Log2(Intensity) +/- StdErr**

**Variability Gauge Rank=558, protein_id=IPI00303120.3, Annotation=Transmembrane_protein_143**

**Variability Chart for Mean Log2(Intensity) +/- StdErr**

**Variability Gauge Rank=559, protein_id=IPI00010433.3, Annotation=DNA-binding_protein_SATB2**

**Variability Chart for Mean Log2(Intensity) +/- StdErr**

**Variability Gauge Rank=560, protein_id=IPI00556590.1, Annotation=CDNA_FLJ40085_fis,_clone_TESTI2002993**

**Variability Chart for Mean Log2(Intensity) +/- StdErr**

**Variability Gauge Rank=561, protein_id=IPI00001727.2, Annotation=stromal_interaction_molecule_2**

**Variability Chart for Mean Log2(Intensity) +/- StdErr**

**Variability Gauge Rank=562, protein_id=IPI00644301.1, Annotation=Utrophin**

**Variability Chart for Mean Log2(Intensity) +/- StdErr**

**Variability Gauge Rank=563, protein_id=IPI00024915.2, Annotation=Isoform_Mitochondrial_of_Peroxiredoxin-5,_mitochondrial_precursor**

**Variability Chart for Mean Log2(Intensity) +/- StdErr**

**Variability Gauge Rank=564, protein_id=IPI00419662.2, Annotation=RGPG542**

**Variability Chart for Mean Log2(Intensity) +/- StdErr**

**Variability Gauge Rank=565, protein_id=IPI00009904.1, Annotation=Protein_disulfide-isomerase_A4_precursor**

**Variability Chart for Mean Log2(Intensity) +/- StdErr**

**Variability Gauge Rank=566, protein_id=IPI00165045.3, Annotation=calcium_channel,_voltage-dependent,_alpha_1E_subunit**

**Variability Chart for Mean Log2(Intensity) +/- StdErr**

**Variability Gauge Rank=567, protein_id=IPI00012048.1, Annotation=Nucleoside_diphosphate_kinase_A**

**Variability Chart for Mean Log2(Intensity) +/- StdErr**

**Variability Gauge Rank=568, protein_id=IPI00006980.1, Annotation=Protein_C14orf166**

**Variability Chart for Mean Log2(Intensity) +/- StdErr**

**Variability Gauge Rank=569, protein_id=IPI00328409.4, Annotation=Armadillo_repeat-containing_protein_4**

**Variability Chart for Mean Log2(Intensity) +/- StdErr**

**Variability Gauge Rank=570, protein_id=IPI00328350.6, Annotation=Niban_protein**

**Variability Chart for Mean Log2(Intensity) +/- StdErr**

**Variability Gauge Rank=571, protein_id=IPI00644135.1, Annotation=123_kDa_protein**

**Variability Chart for Mean Log2(Intensity) +/- StdErr**

**Variability Gauge Rank=572, protein_id=IPI00031545.2, Annotation=Isoform_Long_of_Inositol_1,4,5-trisphosphate_receptor_type_2**

**Variability Chart for Mean Log2(Intensity) +/- StdErr**

**Variability Gauge Rank=573, protein_id=IPI00218936.1, Annotation=Isoform_AML-1A_of_Runt-related_transcription_factor_1**

**Variability Chart for Mean Log2(Intensity) +/- StdErr**

**Variability Gauge Rank=574, protein_id=IPI00026314.1, Annotation=Isoform_1_of_Gelsolin_precursor**

**Variability Chart for Mean Log2(Intensity) +/- StdErr**

**Variability Gauge Rank=575, protein_id=IPI00003449.1, Annotation=Forkhead_box_protein_D1**

**Variability Chart for Mean Log2(Intensity) +/- StdErr**

**Variability Gauge Rank=576, protein_id=IPI00217030.9, Annotation=40S_ribosomal_protein_S4,_X_isoform**

**Variability Chart for Mean Log2(Intensity) +/- StdErr**

**Variability Gauge Rank=577, protein_id=IPI00002521.1, Annotation=ATP_synthase_coupling_factor_6,_mitochondrial_precursor**

**Variability Chart for Mean Log2(Intensity) +/- StdErr**

**Variability Gauge Rank=578, protein_id=IPI00026302.3, Annotation=60S_ribosomal_protein_L31**

**Variability Chart for Mean Log2(Intensity) +/- StdErr**

**Variability Gauge Rank=579, protein_id=IPI00552093.3, Annotation=Amphoterin-induced_protein_3_precursor**

**Variability Chart for Mean Log2(Intensity) +/- StdErr**

**Variability Gauge Rank=580, protein_id=IPI00025039.1, Annotation=rRNA_2'-O-methyltransferase_fibrillarin**

**Variability Chart for Mean Log2(Intensity) +/- StdErr**

**Variability Gauge Rank=581, protein_id=IPI00215914.4, Annotation=ADP-ribosylation_factor_1**

**Variability Chart for Mean Log2(Intensity) +/- StdErr**

**Variability Gauge Rank=582, protein_id=IPI00259687.3, Annotation=124_kDa_protein**

**Variability Chart for Mean Log2(Intensity) +/- StdErr**

**Variability Gauge Rank=583, protein_id=24981014, Annotation=MASP1_protein_[Homo_sapiens]**

**Variability Chart for Mean Log2(Intensity) +/- StdErr**

**Variability Gauge Rank=584, protein_id=IPI00478909.2, Annotation=Similar_to_KRAB/zinc_finger_suppressor_protein_1**

**Variability Chart for Mean Log2(Intensity) +/- StdErr**

**Variability Gauge Rank=585, protein_id=IPI00166143.2, Annotation=Isoform_4_of_Transcription_factor_SOX-5**

**Variability Chart for Mean Log2(Intensity) +/- StdErr**

**Variability Gauge Rank=586, protein_id=IPI00216920.3, Annotation=OTTHUMP00000030235**

**Variability Chart for Mean Log2(Intensity) +/- StdErr**

**Variability Gauge Rank=587, protein_id=IPI00002618.1, Annotation=ATP-binding_cassette_sub-family_D_member_2**

**Variability Chart for Mean Log2(Intensity) +/- StdErr**

**Variability Gauge Rank=588, protein_id=IPI00044583.1, Annotation=Isoform_1_of_Transcriptional-regulating_factor_1**

**Variability Chart for Mean Log2(Intensity) +/- StdErr**

**Variability Gauge Rank=589, protein_id=IPI00374686.4, Annotation=29_kDa_protein**

**Variability Chart for Mean Log2(Intensity) +/- StdErr**

**Variability Gauge Rank=590, protein_id=IPI00000846.1, Annotation=Isoform_1_of_Chromodomain_helicase-DNA-binding_protein_4**

**Variability Chart for Mean Log2(Intensity) +/- StdErr**

**Variability Gauge Rank=591, protein_id=IPI00328932.5, Annotation=Isoform_1_of_Vacuolar_protein_sorting_protein_36**

**Variability Chart for Mean Log2(Intensity) +/- StdErr**

**Variability Gauge Rank=592, protein_id=IPI00146329.3, Annotation=Isoform_1_of_Uncharacterized_protein_C9orf90**

**Variability Chart for Mean Log2(Intensity) +/- StdErr**

**Variability Gauge Rank=593, protein_id=IPI00288941.1, Annotation=Nuclear_receptor_coactivator_5**

**Variability Chart for Mean Log2(Intensity) +/- StdErr**

**Variability Gauge Rank=594, protein_id=IPI00178743.4, Annotation=ALMS1**

**Variability Chart for Mean Log2(Intensity) +/- StdErr**

**Variability Gauge Rank=595, protein_id=IPI00010270.1, Annotation=Ras-related_C3_botulinum_toxin_substrate_2_precursor**

**Variability Chart for Mean Log2(Intensity) +/- StdErr**

**Variability Gauge Rank=596, protein_id=IPI00045496.1, Annotation=Isoform_1_of_Ubiquitin_carboxyl-terminal_hydrolase_28**

**Variability Chart for Mean Log2(Intensity) +/- StdErr**

**Variability Gauge Rank=597, protein_id=IPI00184296.4, Annotation=32_kDa_protein**

**Variability Chart for Mean Log2(Intensity) +/- StdErr**

**Variability Gauge Rank=598, protein_id=IPI00289116.2, Annotation=Hypothetical_protein_FLJ23960**

**Variability Chart for Mean Log2(Intensity) +/- StdErr**

**Variability Gauge Rank=599, protein_id=IPI00021131.3, Annotation=Leucine-rich_repeat-containing_G-protein_coupled_receptor_5_precursor**

**Variability Chart for Mean Log2(Intensity) +/- StdErr**

**Variability Gauge Rank=600, protein_id=IPI00217537.2, Annotation=Isoform_1_of_Putative_Polycomb_group_protein_ASXL1**

**Variability Chart for Mean Log2(Intensity) +/- StdErr**

**Variability Gauge Rank=601, protein_id=IPI00029731.8, Annotation=60S_ribosomal_protein_L35a**

**Variability Chart for Mean Log2(Intensity) +/- StdErr**

**Variability Gauge Rank=602, protein_id=IPI00025418.1, Annotation=Collagen_alpha-1(VII)_chain_precursor**

**Variability Chart for Mean Log2(Intensity) +/- StdErr**

**Variability Gauge Rank=603, protein_id=IPI00017608.1, Annotation=DnaJ_homolog_subfamily_B_member_9**

**Variability Chart for Mean Log2(Intensity) +/- StdErr**

**Variability Gauge Rank=604, protein_id=7022010, Annotation=unnamed_protein_product_[Homo_sapiens]**

**Variability Chart for Mean Log2(Intensity) +/- StdErr**

**Variability Gauge Rank=605, protein_id=IPI00292892.3, Annotation=KIAA1403_protein_(Fragment)**

**Variability Chart for Mean Log2(Intensity) +/- StdErr**

**Variability Gauge Rank=606, protein_id=126376, Annotation=LAMP1_HUMAN_Lysosome-associated_membrane_glycoprotein_1_precursor_(LAMP-1)_(CD107a_antigen)**

**Variability Chart for Mean Log2(Intensity) +/- StdErr**

**Variability Gauge Rank=607, protein_id=IPI00024082.1, Annotation=Myocyte-specific_enhancer_factor_2B**

**Variability Chart for Mean Log2(Intensity) +/- StdErr**

**Variability Gauge Rank=608, protein_id=IPI00100630.5, Annotation=Protein_ENL**

**Variability Chart for Mean Log2(Intensity) +/- StdErr**

**Variability Gauge Rank=609, protein_id=IPI00398272.4, Annotation=Collagen_alpha-1(XVII)_chain**

**Variability Chart for Mean Log2(Intensity) +/- StdErr**

**Variability Gauge Rank=610, protein_id=IPI00643731.1, Annotation=Collagen,_type_XVII,_alpha_1**

**Variability Chart for Mean Log2(Intensity) +/- StdErr**

**Variability Gauge Rank=611, protein_id=IPI00027831.1, Annotation=Glutamate-rich_WD_repeat-containing_protein_1**

**Variability Chart for Mean Log2(Intensity) +/- StdErr**

**Variability Gauge Rank=612, protein_id=IPI00217473.4, Annotation=Hemoglobin_subunit_zeta**

**Variability Chart for Mean Log2(Intensity) +/- StdErr**

**Variability Gauge Rank=613, protein_id=IPI00021812.1, Annotation=Neuroblast_differentiation-associated_protein_AHNAK_(Fragment)**

**Variability Chart for Mean Log2(Intensity) +/- StdErr**

**Variability Gauge Rank=614, protein_id=IPI00253835.5, Annotation=Isoform_1_of_Protein_C6orf170**

**Variability Chart for Mean Log2(Intensity) +/- StdErr**

**Variability Gauge Rank=615, protein_id=IPI00061376.3, Annotation=ELP4_protein**

**Variability Chart for Mean Log2(Intensity) +/- StdErr**

**Variability Gauge Rank=616, protein_id=IPI00445401.2, Annotation=Isoform_2_of_HECT,_UBA_and_WWE_domain-containing_protein_1**

**Variability Chart for Mean Log2(Intensity) +/- StdErr**

**Variability Gauge Rank=617, protein_id=IPI00032957.1, Annotation=SUMO-1-conjugating_enzyme_UBC9**

**Variability Chart for Mean Log2(Intensity) +/- StdErr**

**Variability Gauge Rank=618, protein_id=IPI00152739.3, Annotation=ATH1,_acid_trehalase-like_1**

**Variability Chart for Mean Log2(Intensity) +/- StdErr**

**Variability Gauge Rank=619, protein_id=IPI00022431.1, Annotation=Alpha-2-HS-glycoprotein_precursor**

**Variability Chart for Mean Log2(Intensity) +/- StdErr**

**Variability Gauge Rank=620, protein_id=IPI00021250.3, Annotation=Death-associated_protein_kinase_1**

**Variability Chart for Mean Log2(Intensity) +/- StdErr**

**Variability Gauge Rank=621, protein_id=IPI00020692.3, Annotation=Isoform_1_of_Sodium_channel_protein_type_3_subunit_alpha**

**Variability Chart for Mean Log2(Intensity) +/- StdErr**

**Variability Gauge Rank=622, protein_id=IPI00004121.1, Annotation=Isoform_2_of_Protein_Wnt-2b_precursor**

**Variability Chart for Mean Log2(Intensity) +/- StdErr**

**Variability Gauge Rank=623, protein_id=IPI00096899.6, Annotation=DNA_replication_factor_Cdt1**

**Variability Chart for Mean Log2(Intensity) +/- StdErr**

**Variability Gauge Rank=624, protein_id=IPI00446743.1, Annotation=CDNA_FLJ41197_fis,_clone_BRACE2045947**

**Variability Chart for Mean Log2(Intensity) +/- StdErr**

**Variability Gauge Rank=625, protein_id=IPI00183706.3, Annotation=KIF19_protein_(Fragment)**

**Variability Chart for Mean Log2(Intensity) +/- StdErr**

**Variability Gauge Rank=626, protein_id=IPI00025366.4, Annotation=Citrate_synthase,_mitochondrial_precursor**

**Variability Chart for Mean Log2(Intensity) +/- StdErr**

**Variability Gauge Rank=627, protein_id=IPI00289907.5, Annotation=forty-two-three_domain_containing_1_isoform_1**

**Variability Chart for Mean Log2(Intensity) +/- StdErr**

**Variability Gauge Rank=628, protein_id=IPI00398922.4, Annotation=Similar_to_Protein_phosphatase_1_regulatory_subunit_14B**

**Variability Chart for Mean Log2(Intensity) +/- StdErr**

**Variability Gauge Rank=629, protein_id=IPI00020985.3, Annotation=E1A-associated_protein_p300**

**Variability Chart for Mean Log2(Intensity) +/- StdErr**

**Variability Gauge Rank=630, protein_id=IPI00384507.1, Annotation=WUGSC:H_248O15.1_protein_(Fragment)**

**Variability Chart for Mean Log2(Intensity) +/- StdErr**

**Variability Gauge Rank=631, protein_id=IPI00059240.1, Annotation=Insulin_gene_enhancer_protein_ISL-2**

**Variability Chart for Mean Log2(Intensity) +/- StdErr**

**Variability Gauge Rank=632, protein_id=IPI00032525.4, Annotation=Conserved_hypothetical_protein**

**Variability Chart for Mean Log2(Intensity) +/- StdErr**

**Variability Gauge Rank=633, protein_id=IPI00259856.4, Annotation=Alpha-2,8-sialyltransferase_8F**

**Variability Chart for Mean Log2(Intensity) +/- StdErr**

**Variability Gauge Rank=634, protein_id=IPI00297578.4, Annotation=downregulated_in_ovarian_cancer_1_isoform_1**

**Variability Chart for Mean Log2(Intensity) +/- StdErr**

**Variability Gauge Rank=635, protein_id=IPI00307259.10, Annotation=DnaJ_homolog_subfamily_C_member_13**

**Variability Chart for Mean Log2(Intensity) +/- StdErr**

**Variability Gauge Rank=636, protein_id=IPI00395632.4, Annotation=ALK_tyrosine_kinase_receptor_precursor**

**Variability Chart for Mean Log2(Intensity) +/- StdErr**

**Variability Gauge Rank=637, protein_id=IPI00009771.5, Annotation=Lamin_B2**

**Variability Chart for Mean Log2(Intensity) +/- StdErr**

**Variability Gauge Rank=638, protein_id=IPI00056494.4, Annotation=60S_ribosomal_protein_L36a-like**

**Variability Chart for Mean Log2(Intensity) +/- StdErr**

**Variability Gauge Rank=639, protein_id=IPI00445315.2, Annotation=Family_with_sequence_similarity_47,_member_C**

**Variability Chart for Mean Log2(Intensity) +/- StdErr**

**Variability Gauge Rank=640, protein_id=IPI00374095.1, Annotation=66_kDa_protein**

**Variability Chart for Mean Log2(Intensity) +/- StdErr**

**Variability Gauge Rank=641, protein_id=IPI00168047.1, Annotation=CDNA_FLJ35093_fis,_clone_PLACE6006137**

**Variability Chart for Mean Log2(Intensity) +/- StdErr**

**Variability Gauge Rank=642, protein_id=IPI00012750.3, Annotation=40S_ribosomal_protein_S25**

**Variability Chart for Mean Log2(Intensity) +/- StdErr**

**Variability Gauge Rank=643, protein_id=IPI00005668.4, Annotation=Aldo-keto_reductase_family_1_member_C2**

**Variability Chart for Mean Log2(Intensity) +/- StdErr**

**Variability Gauge Rank=644, protein_id=IPI00009521.1, Annotation=Macrophage_receptor_MARCO**

**Variability Chart for Mean Log2(Intensity) +/- StdErr**

**Variability Gauge Rank=645, protein_id=IPI00217340.2, Annotation=Isoform_1_of_WW_domain-containing_protein_1**

**Variability Chart for Mean Log2(Intensity) +/- StdErr**

**Variability Gauge Rank=646, protein_id=IPI00030144.1, Annotation=Peptidyl-prolyl_cis-trans_isomerase**

**Variability Chart for Mean Log2(Intensity) +/- StdErr**
