# Supplementary material for: Genomic and Proteomic Analysis of the Impact of Mitotic Quiescence on the Engraftment of Human CD34+ Cells
Source: PLoS One. 2011 Mar 7;6(3):e17498. doi: 10.1371/journal.pone.0017498 (PMC3049784; doi:10.1371/journal.pone.0017498)
Supplement: Table S5 — Differentially expressed proteins between G0 and G1 cells of BM, MPB, and UCB. (A): Differentially expressed proteins between G0 and G1 cells of BM. (B): Differentially expressed proteins between G0 and G1 cells of MPB. (C): Differentially expressed proteins between G0 and G1 cells of UCB. (DOC) [file pone.0017498.s006.doc]

**Table S5.** Differentially expressed proteins between G0 and G1 cells of BM, MPB, and UCB.

**(A):** Differentially expressed proteins between G0 and G1 cells of BM.

| **P** | **Protein_ID** | **Annotation** | **Best_Sequence** | **FC*** |
| --- | --- | --- | --- | --- |
| 1 | 3659957 | The solution structure of reduced monomeric superoxide dismutase, Nmr | GDGPVQGIINFEQK | -1.5 |
| 1 | 5822065 | Superoxide dismutase mutant, Chain A | GDGPVQGIINFEQK | -1.5 |
| 1 | IPI00007244.1 | Isoform H17 of myeloperoxidase precursor | VGPLLACIIGTQFR | 1.5 |
| 1 | IPI00025491.1 | Eukaryotic initiation factor 4A-I | YGFEKPSAIQQR | 1.5 |
| 1 | IPI00006690.1 | Eosinophil peroxidase precursor | IPCFLAGDTR | 1.5 |
| 2 | IPI00000606.4**^c^** | Tetratricopeptide repeat protein 4 | HLKAIIR | -2.1 |
| 2 | IPI00016387.4**^c^** | Pre-mRNA cleavage complex II protein Pcf11 | HLKNAIR | -2.1 |
| 2 | IPI00787038.1**^c^** | Predicted: family with sequence similarity 92, member A3 | HLLQALR | -1.9 |
| 2 | IPI00005715.5**^a^** | Isoform 1 of ubiquitin conjugation factor E4 B | RYIRRLR | -1.9 |
| 2 | IPI00479962.3**^c^** | Myosin-5B | HLRRIR | -1.8 |
| 2 | IPI00024046.1 | Cadherin-13 precursor | NITAVGKTLFVHAR | -1.7 |
| 2 | IPI00013214.1 | DNA replication licensing factor MCM3 | TVDLQDAEEAVELVQYAYFK | -1.6 |
| 2 | IPI00027255.1**^c^** | Myosin light polypeptide 6B | ILYSQCGDVMR | -1.6 |
| 2 | IPI00007858.1 | Myosin-13 | QIIQANPLLEAFGNAK | -1.5 |
| 2 | IPI00216318.5 | Tyrosine 3-monooxygenase / tryptophan 5-monooxygenase activation protein, beta polypeptide | NLLSVAYK | -1.5 |
| 2 | IPI00021836.1**^c^** | Isoform 1 of prorelaxin H2 precursor | NKDTETINMMSEFVANL | -1.5 |
| 2 | IPI00022314.1**^c^** | Superoxide dismutase [Mn], mitochondrial precursor | AIWNVINWENVTER | -1.5 |
| 2 | 2760897 | Natural killer cell inhibitory receptor KIR2DL3 variant | EGKFKDTLR | 1.5 |
| 2 | 49457412 | RPLP1 | ALILHDDEVTVTEDKINALIK | 1.6 |
| 2 | IPI00008527.3 | 60S acidic ribosomal protein P1 | ALILHDDEVTVTEDKINALIK | 1.6 |
| 2 | IPI00299584.3 | Isoform 1 of tripartite motif-containing protein 15 | RCEMKTFVSPE | 1.6 |
| 2 | IPI00290770.3**^be^** | Chaperonin containing TCP1, subunit 3 isoform b | GISDLAQHYLMR | 1.8 |

**Table S5.** Continued.

| **P** | **Protein_ID** | **Annotation** | **Best_Sequence** | **FC*** |
| --- | --- | --- | --- | --- |
| 2 | IPI00167706.4 | cDNA FLJ37464 fis, clone BRAWH2011795, weakly similar to liver carboxylesterase | MRFLQLNFQR | 2.1 |
| 2 | IPI00411985.2 | Carboxylesterase | MRFLQLNFQR | 2.1 |
| 3 | IPI00644597.1 | Hypothetical protein | GAPSGGCRP | -1.5 |
| 4 | IPI00024568.1**^a^** | Glioma tumor suppressor candidate region gene 1 protein | QLQFPPSQ | -3.6 |
| 4 | IPI00217952.6**^c^** | Isoform 1 of glucosamine--fructose-6-phosphate aminotransferase [isomerizing] 1 | RTAGDHPGR | -2.4 |
| 4 | IPI00644301.1 | Utrophin | MAVVRTSL | -2.2 |
| 4 | IPI00146329.3**^be^** | Isoform 1 of uncharacterized protein C9orf90 | SRIALNSAK | -2.0 |
| 4 | IPI00216920.3**^be^** | OTTHUMP00000030235 | VEKANEAAK | -1.9 |
| 4 | IPI00306043.1**^c^** | Isoform 1 of YTH domain family protein 2 | NGIAGSSLPPPPIK | -1.9 |
| 4 | IPI00012386.1**^c^** | Cochlin precursor | LLLPGPAGSE | -1.7 |
| 4 | IPI00013297.1**^c^** | 28 kDa heat- and acid-stable phosphoprotein | EGGDGAAGDPK | -1.7 |
| 4 | IPI00001727.2 | Stromal interaction molecule 2 | HITIEDLWK | -1.7 |
| 4 | IPI00438271.1**^c^** | Uromodulin-like 1 protein variant 7 (Fragment) | EVISVQMSMSVSMRSSMPAL | -1.7 |
| 4 | IPI00186970.5 | Hypothetical protein RP11-293F5.5 | MVVSAGPWSGEK | -1.6 |
| 4 | IPI00302962.1 | Amphiphysin I variant CT4 (Fragment) | TLKINEVETR | -1.6 |
| 4 | IPI00084684.8 | Predicted: similar to zinc finger protein 469 | RGSSPILSEGSL | -1.6 |
| 4 | IPI00014186.3**^a^** | Isoform A of alpha-fetoprotein enhancer-binding protein | EPLPADLAQ | -1.6 |
| 4 | IPI00065280.2**^a^** | Protein C21orf129 | LLIPRWR | -1.6 |
| 4 | IPI00017443.3 | Absent in melanoma 1-like | HPGVGLTSGSSR | -1.5 |
| 4 | IPI00419662.2 | RGPG542 | LLLGLLLAAG | -1.5 |
| 4 | IPI00100630.5**^c^** | Protein ENL | EPSQNHSQGPLR | -1.5 |
| 4 | IPI00178953.3 | Isoform 4 of zinc finger protein 638 | WMNLLLWMR | -1.5 |
| 4 | IPI00018353.2 | Isoform 1 of cohesin subunit SA-3 | LLAGFCK | -1.5 |
| 4 | IPI00328350.6**^c^** | Niban protein | LESVQPFLASILEELMGP | -1.5 |
| 4 | IPI00000201.2**^be^** | Isoform 1 of low-density lipoprotein receptor-related protein 3 precursor | GLIPP | 1.5 |
| 4 | IPI00470682.4 | Arginyl aminopeptidase-like 1 | FTSVVAQDLLDSFLSFFPEL | 1.5 |
| 4 | IPI00003391.4 | 305 kDa protein | SPAHK | 1.5 |
| 4 | IPI00218936.1 | Isoform AML-1A of Runt-related transcription factor 1 | HSNSPTNMGGASCSRQA | 1.5 |

**Table S5.** Continued.

| **P** | **Protein_ID** | **Annotation** | **Best_Sequence** | **FC*** |
| --- | --- | --- | --- | --- |
| 4 | IPI00445401.2 | Isoform 2 of HECT, UBA and WWE domain-containing protein 1 | SSDPLGDTASNLGSAVDELMR | 1.5 |
| 4 | IPI00016763.3 | GTP-binding protein | MPLHRSNLK | 1.5 |
| 4 | 1877499**^a^** | Polyhomeotic 1 homolog | GMPGTVQSGQALK | 1.6 |
| 4 | IPI00022043.1**^c^** | Hamartin | ILELESHLAK | 1.6 |
| 4 | IPI00008711.3**^a^** | Wolframin | KAALVMYWK | 1.7 |
| 4 | IPI00102575.2**^c^** | ATP(GTP)-binding protein | DVVDLSESLPLAEELNLLK | 1.8 |
| 4 | IPI00304587.3**^a^** | Dispatched B | RLLLALHRR | 1.8 |
| 4 | IPI00292221.3 | Ribosome production factor 1 | VGIQELGPR | 1.8 |
| 4 | IPI00418497.1 | Isoform 2 of Import inner membrane translocase subunit TIM50, mitochondrial precursor | RGGGALQGPR | 1.8 |
| 4 | IPI00221285.5 | Ankyrin repeat domain 50 | NMSLTLEDFQR | 1.9 |
| 4 | IPI00442305.1 | CDNA FLJ16182 fis, clone BRTHA2002133 | QEKEIKDIQIGK | 3.0 |

**(B):** Differentially expressed proteins between G0 and G1 cells of MPB.

| 1 | IPI00010779.3 | Tropomyosin 4 | HIAEEADRKYEEVAR | 1.5 |
| --- | --- | --- | --- | --- |
| 1 | IPI00014581.1 | Isoform 1 of Tropomyosin 1 alpha chain | LATALQKLEEAEKAADESERGMK | 1.7 |
| 2 | IPI00176678.9 | Predicted: similar to alcohol dehydrogenase class 3 chi chain | IIGVDINKDKFAR | -2.0 |
| 2 | IPI00005715.5**^a^** | Isoform 1 of ubiquitin conjugation factor E4 B | RYIRRLR | -1.6 |
| 2 | IPI00298237.7 | Isoform 1 of tripeptidyl-peptidase 1 precursor | LFGGNFAHQASVAR | -1.6 |
| 2 | 16974825 | Solution structure of calcium-calmodulin N-terminal domain, Chain A | XDQLTEEQIAEFKEAFSLFDKDGDGTITTK | -1.5 |
| 2 | IPI00027202.2 | Zinc finger Y-chromosomal protein | AVAAAAAAVHEQQIDED | -1.5 |
| 2 | IPI00742127.2**^d^** | Pseudogene candidate | GFGFVTYATVEEVDAAMNARPHKVDGR | 1.5 |
| 2 | IPI00021107.4**^d^** | Xylulokinase homolog | TKILATGGASHNR | 1.6 |
| 2 | IPI00000230.5 | Tropomyosin1 α-chain isoform 2 | LATALQKLEEAEKAADESERGMK | 1.8 |
| 2 | IPI00328587.4 | ENO1P protein | KLNVTEQEKIDKLMIEMDGTENKSK | 1.9 |
| 2 | IPI00290770.3**^be^** | Chaperonin containing TCP1, subunit 3 isoform b | GISDLAQHYLMR | 1.9 |
| 4 | IPI00259856.4 | Alpha-2,8-sialyltransferase 8F | TLRSPATAVPR | -1.8 |

**Table S5.** Continued.

| **P** | **Protein_ID** | **Annotation** | **Best_Sequence** | **FC*** |
| --- | --- | --- | --- | --- |
| 4 | 126376**^d^** | Lysosome-associated membrane glycoprotein 1 precursor (LAMP-1) (CD107a antigen) | MAPRSAR | -1.6 |
| 4 | IPI00006980.1 | Protein C14orf166 | EGLPVALDKHILGFDTGDAVLNEAAQILR | -1.6 |
| 4 | IPI00014186.3**^a^** | Isoform A of alpha-fetoprotein enhancer-binding protein | EPLPADLAQ | -1.6 |
| 4 | IPI00065280.2**^a^** | Protein C21orf129 | LLIPRWR | -1.6 |
| 4 | IPI00000201.2**^be^** | Isoform 1 of low-density lipoprotein receptor-related protein 3 precursor | GLIPP | -1.5 |
| 4 | IPI00328932.5 | Isoform 1 of vacuolar protein sorting protein 36 | LSKLMIKAKEMVELS | 1.5 |
| 4 | IPI00146329.3**^be^** | Isoform 1 of uncharacterized protein C9orf90 | SRIALNSAK | 1.5 |
| 4 | IPI00004657.1 | HLA class I histocompatibility antigen, B-7 alpha chain precursor | SSGGKG | 1.7 |
| 4 | IPI00216920.3**^be^** | OTTHUMP00000030235 | VEKANEAAK | 1.7 |
| 4 | IPI00292674.1 | WD repeat domain 21A isoform 2 | DSHILLCLMGLAETPGCATLLP | 1.7 |
| 4 | IPI00017608.1 | DnaJ homolog subfamily B member 9 | NKSPDAEAK | 1.9 |
| 4 | IPI00024568.1**^a^** | Glioma tumor suppressor candidate region gene 1 protein | QLQFPPSQ | 2.0 |
| 4 | IPI00304587.3**^a^** | Dispatched B | RLLLALHRR | 2.2 |
| 4 | 1877499**^a^** | Polyhomeotic 1 homolog | GMPGTVQSGQALK | 3.1 |
| 4 | IPI00008711.3**^a^** | Wolframin | KAALVMYWK | 4.1 |

**(C):** Differentially expressed proteins between G0 and G1 cells of UCB.

| 1 | IPI00296291.2 | HP1-BP74 | SSAVDPEPQVKLEDVLPLAFTR | -1.5 |
| --- | --- | --- | --- | --- |
| 2 | IPI00005715.5**^a^** | Isoform 1 of ubiquitin conjugation factor E4 B | RYIRRLR | -5.4 |
| 2 | IPI00021107.4**^d^** | Xylulokinase homolog | TKILATGGASHNR | -2.2 |
| 2 | IPI00027255.1**^c^** | Myosin light polypeptide 6B | ILYSQCGDVMR | -1.8 |
| 2 | IPI00027252.6 | Prohibitin-2 | IYLTADNLVLNLQDESFTR | -1.8 |
| 2 | IPI00016387.4**^c^** | Pre-mRNA cleavage complex II protein Pcf11 | HLKNAIR | -1.8 |
| 2 | IPI00479962.3**^c^** | Myosin-5B | HLRRIR | -1.8 |
| 2 | IPI00787038.1**^c^** | Predicted: family with sequence similarity 92, member A3 | HLLQALR | -1.8 |
| 2 | IPI00000606.4**^c^** | Tetratricopeptide repeat protein 4 | HLKAIIR | -1.7 |
| 2 | IPI00021836.1**^c^** | Isoform 1 of Prorelaxin H2 precursor | NKDTETINMMSEFVANL | -1.6 |

**Table S5.** Continued.

| **P** | **Protein_ID** | **Annotation** | **Best_Sequence** | **FC*** |
| --- | --- | --- | --- | --- |
| 2 | IPI00304692.1 | Heterogeneous nuclear ribonucleoprotein G | AIKVEQATKPSFESGR | -1.6 |
| 2 | IPI00022314.1**^c^** | Superoxide dismutase [Mn], mitochondrial precursor | AIWNVINWENVTER | -1.5 |
| 2 | IPI00742127.2**^d^** | Pseudogene candidate | GFGFVTYATVEEVDAAMNARPHKVDGR | -1.5 |
| 2 | IPI00183913.2 | Bone specific CMF608 | SNGPQSYQYLIASNGSFIISKTTR | -1.5 |
| 2 | IPI00174347.5 | Novel protein | AIYPNEGFLK | -1.5 |
| 2 | IPI00005613.2 | Splicing factor U2AF 35 kDa subunit | GGFCNFMHLKPISR | -1.5 |
| 2 | IPI00215790.5 | 60S ribosomal protein L38 | KIEEIKDFLLTAR | -1.5 |
| 2 | IPI00001453.2 | Alpha-internexin | HLREYQDLLNVK | 1.5 |
| 2 | IPI00030985.1 | Protein FAM96A | VLWLSGLSEPGAARQPR | 1.5 |
| 2 | IPI00024114.3 | Putative GTP-binding protein 5 | VASYPFTTLKPHVG | 1.6 |
| 2 | IPI00291005.7 | Malate dehydrogenase, cytoplasmic | SAPSIPKENFSCLTR | 1.7 |
| 2 | IPI00002135.1 | Transforming acidic coiled-coil-containing protein 3 | ENVPPKNLAK | 4.2 |
| 3 | IPI00026174.1 | Cholecystokinins precursor | HLGALLAR | -1.5 |
| 3 | IPI00102300.3 | Isoform 3 of Platelet glycoprotein VI precursor | GCDPNNPGGVSGR | 1.5 |
| 4 | 126376**^d^** | Lysosome-associated membrane glycoprotein 1 precursor (LAMP-1) (CD107a antigen) | MAPRSAR | -3.3 |
| 4 | IPI00024568.1**^a^** | Glioma tumor suppressor candidate region gene 1 protein | QLQFPPSQ | -2.8 |
| 4 | IPI00219221.2 | Galectin-7 | SSLPEGIRPGTVLR | -2.7 |
| 4 | IPI00025683.2 | Isoform 1 of transforming acidic coiled-coil-containing protein 1 | ESMDPFKPTT | -2.3 |
| 4 | IPI00306043.1**^c^** | Isoform 1 of YTH domain family protein 2 | NGIAGSSLPPPPIK | -2.2 |
| 4 | IPI00012386.1**^c^** | Cochlin precursor | LLLPGPAGSE | -2.0 |
| 4 | IPI00253835.5 | Isoform 1 of protein C6orf170 | HIAGILAR | -2.0 |
| 4 | IPI00014186.3**^a^** | Isoform A of alpha-fetoprotein enhancer-binding protein | EPLPADLAQ | -1.9 |
| 4 | IPI00065280.2**^a^** | Protein C21orf129 | LLIPRWR | -1.9 |
| 4 | IPI00001513.1 | Isoform 1 of Protocadherin alpha 10 precursor | EMMYSFSSLVPPTIRRK | -1.7 |
| 4 | IPI00013297.1**^c^** | 28 kDa heat- and acid-stable phosphoprotein | EGGDGAAGDPK | -1.7 |
| 4 | IPI00100630.5**^c^** | Protein ENL | EPSQNHSQGPLR | -1.7 |
| 4 | IPI00438271.1**^c^** | Uromodulin-like 1 protein variant 7 (Fragment) | EVISVQMSMSVSMRSSMPAL | -1.7 |

**Table S5.** Continued.

| **P** | **Protein_ID** | **Annotation** | | **Best_Sequence** | | **FC*** |
| --- | --- | --- | --- | --- | --- | --- |
| 4 | IPI00217952.6**^c^** | Isoform 1 of glucosamine--fructose-6-phosphate aminotransferase [isomerizing] 1 | | RTAGDHPGR | | -1.6 |
| 4 | IPI00003907.1 | Isoform 1 of Protocadherin gamma C5 precursor | | EENGRYFSLSLMSGALAVNQ | | -1.6 |
| 4 | IPI00457338.2 | Predicted: similar to 60S ribosomal protein L6 | | MPTYYLTEDVLSKLLSQGK | | -1.6 |
| 4 | IPI00328350.6**^c^** | Niban protein | | LESVQPFLASILEELMGP | | -1.6 |
| 4 | IPI00328285.5 | Ubiquitin carboxyl-terminal hydrolase 20 | | RGLTGMKNLGNSCYMNAALQ | | -1.6 |
| 4 | IPI00061078.5 | CDNA FLJ30638 fis, clone CTONG2002721, weakly similar to vacuolar protein sorting-associated protein VPS13. | | MPWPFSESIKKRACRYL | | -1.5 |
| 4 | IPI00022043.1**^c^** | Hamartin | | ILELESHLAK | | 1.5 |
| 4 | IPI00004121.1 | Isoform 2 of protein Wnt-2b precursor | | APDGS | | 1.6 |
| 4 | IPI00102575.2**^c^** | ATP(GTP)-binding protein | | DVVDLSESLPLAEELNLLK | | 1.8 |
| 4 | 1877499**^a^** | Polyhomeotic 1 homolog | | GMPGTVQSGQALK | | 1.8 |
| 4 | IPI00008711.3**^a^** | Wolframin | | KAALVMYWK | | 3.1 |
| 4 | IPI00304587.3**^a^** | Dispatched B | | RLLLALHRR | | 4.5 |
|  | **^a^** = common to BM, MPB, and UCB  **^b^** = common to BM and MPB  **^c^** = common to BM and UCB  **^d^** = common to MPB and UCB | | **^e^** = common to BM, MPB, and not UCB  FC* = Fold Change (G0/G1)  P = Priority | |  | |
